# Four-layer folding framework: design, GAP synthesis, and aggregation-induced emission

**DOI:** 10.3389/fchem.2023.1259609

**Published:** 2023-08-10

**Authors:** Sai Zhang, Daixiang Chen, Jia-Yin Wang, Shenghu Yan, Guigen Li

**Affiliations:** ^1^ Continuous Flow Engineering Laboratory of National Petroleum and Chemical Industry, Changzhou University, Changzhou, Jiangsu, China; ^2^ Department of Chemistry and Biochemistry, Texas Tech University, Lubbock, TX, United States

**Keywords:** multilayer folding molecules, Suzuki–Miyaura coupling, GAP chemistry, phosphine oxides, AIE

## Abstract

The design and synthesis of a type of [1 + 4 + 2] four-layer framework have been conducted by taking advantage of Suzuki–Miyaura cross-coupling and group-assisted purification (GAP) chemistry. The optimized coupling of double-layer diboronic esters with 1-bromo-naphth-2-yl phosphine oxides resulted in a series of multilayer folding targets, showing a broad scope of substrates and moderate to excellent yields. The final products were purified using group-assisted purification chemistry/technology, achieved simply by washing crude products with 95% EtOH without the use of chromatography and recrystallization. The structures were fully characterized and assigned by performing X-ray crystallographic analysis. UV–vis absorption, photoluminescence (PL), and aggregation-induced emission (AIE) were studied for the resulting multilayer folding products.

## 1 Introduction

The layered organic structures, including chiral structures, play an important role in biological and material sciences (Moser et al., 1987; Gellman, 1998; Oh et al., 2001; Nakano, 2010; Knouse et al., 2018). The design of these targets is highly demanded to search for desired chemical, physical, and biological properties. This is particularly applicable to the research on multilayer monomers, oligomers, and polymers, which exhibit photoelectronic properties (Wu et al., 2019; Tang et al., 2022a; Tang et al., 2022b; Wang et al., 2022; Xia et al., 2023). For example, a through-space transfer through singlet fission (SF) was proven to involve the absorption of photons by two electronically interacting chromophores to generate a singlet exciton state, which is followed by the rapid formation of two triplet excitons (Chen et al., 2018). Meanwhile, charge-transfer pathways for hybridizing σ and π, and through-space interactions have been proven to be feasible by designing monomeric structures for poly- or copolymerizations (Shen and Chen, 2012; Chen and Shen, 2016; Kawashima et al., 2020; Stará and Starý, 2020; Fujise et al., 2021).

On the other hand, organophosphorus compounds, such as phosphine oxides, are widely applied in a wide range of fields, including medicinal chemistry (Alexandre et al., 2011; Dang et al., 2011), natural products (Kumar et al., 2010), biochemistry (George and Veis, 2008; Chen et al., 2012), catalysis (as catalysts and ligands) (Ackermann et al., 2005; Wang and Wan, 2011), and functional materials (Baumgartner and Réau, 2006; Kirumakki et al., 2009; Baumgartner, 2014). Considering these diverse applications, various methods have been developed for synthesizing these phosphorus-containing compounds (Yin and Buchwald, 2000; Murray et al., 2014; Zhou et al., 2014; Ji et al., 2020; Qian et al., 2020). Innovations in producing organophosphorus compounds, especially those associated with phosphine-containing axial skeletons, have become an attractive topic in chemical synthesis and industry.

**SCHEME 1:**
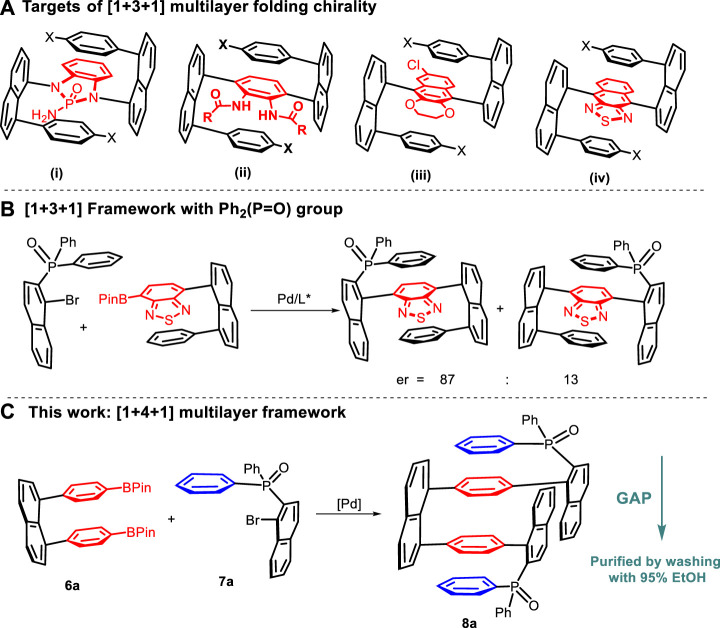
**(A–C)** Multilayer folding frameworks and their assembly.

In the past several years, our group has reported new multi-layer folding chirality of a series of molecules, including oligomers and polymers with structural flexibility, displaying physical properties on UV/Vis absorption, fluorescence, electrochemical performance, aggregation-induced emission (AIE) (Wu et al., 2019; Wu et al., 2019; Liu et al., 2020; Wu et al., 2020; Wu et al., 2021a; Jin et al., 2022a; Tang et al., 2022a; Tang et al., 2022b; Wang et al., 2022), and aggregation-induced polarization (AIP) (Tang et al., 2022c; Tang et al., 2022d). Among them are three-layer compounds (Scheme 1), in which electron-rich (Tang et al., 2022b) and electron-deficient (Jin et al., 2022a) bridges showed distinct impacts on UV–vis absorption and fluorescence behaviors. It is worth noting that many of these compounds showed fluorescence not only in solutions but also in solid states. Very recently, we have established the asymmetric catalytic approach to a [1 + 3+1] type of multi-layer 3D chirality containing the phosphine oxide moiety (Wu et al., 2021a) via chiral amide-phosphine ligands for Suzuki–Miyaura cross-couplings, in which a single asymmetric C–C bond formation led to the efficient control of three-layer chirality.

After achieving the synthesis of three-layer folding chiral targets, our attention is now focused on the design and assembly of four-layered compounds, starting from their racemic counterparts. In the new molecular framework, there are three planar units, including one naphthyl ring, four packed phenyl rings, and two parallel naphthyl rings, which are categorized as a type of [1 + 4 + 2] framework. This is inspired by our early work on the [1 + 3 + 1] framework, in which one packed plane is provided by the (P=O)Ph_2_ group (Wu et al., 2021a). Herein, we report our preliminary results on this endeavor based on new designs and modifications to reaction conditions (Scheme 1).

## 2 Results and discussion

### 2.1 Retro-synthetic analysis (RSA)

**FIGURE 1:**
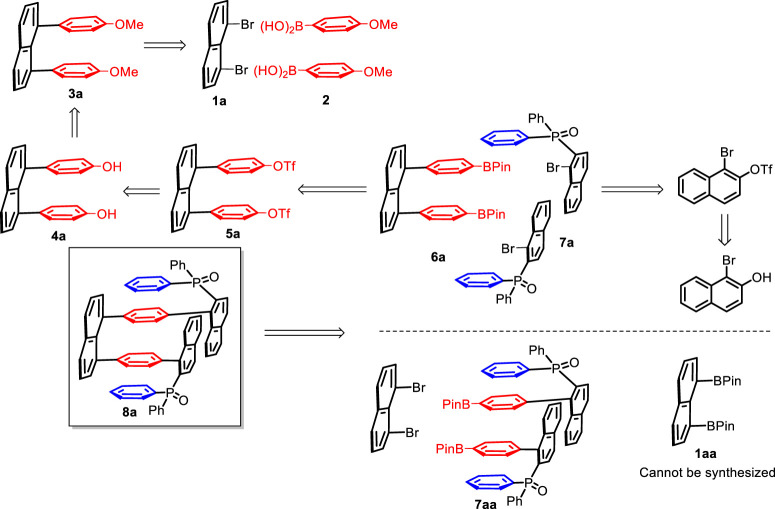
Retro-synthetic analysis of the four-layer framework **8a**.

Retro-synthetic analysis (Corey and Cheng, 2009) revealed that there are several strategies to assemble the four-layer *3D* molecular framework. These strategies are mainly based on utilizing dual Suzuki–Miyaura cross-couplings (Miyaura and Suzuki, 1995) as the key steps, as represented by the case of target 8a, in which two fragments (diboronic ester **1aa** and bromide **1a**) would be joined (Figure 1). In our previous synthesis, boronic esters proved to be more effective than boronic acids in multilayer synthesis via Suzuki–Miyaura couplings. Therefore, they were selected for the present assembly. We made many efforts to synthesizing diboronic ester 1aa for this purpose, but we failed. Similarly, low chemical yields were encountered during the synthesis of diphenyl(1-(4-(4,4,5,5-tetramethyl-1,3,2-dioxaborolan-2-yl)phenyl)naphthalen-2-yl)phosphine oxide 8a. For this reason, they are excluded from our RSA design. Two key precursors, 1,8-bis(4-(4,4,5,5-tetramethyl-1,3,2-dioxaborolan-2- yl)phenyl)naphthalene 6a and (1-bromonaphthalen-2-yl)diphenylphosphine oxide **7a,** can be conveniently obtained, making us choose it as the major route (the top part of Figure 1) for this work. The precursor **6a** was readily derived from the carbon–boron coupling of naphthalene-1,8-diylbis (4,1-phenylene) bis(trifluoromethanesulfonate) **5a**, which originated from the dual Suzuki–Miyaura cross-couplings of 1,8-dibromonaphthalene **1a** with (4-methoxyphenyl)boronic acid **2**, both of which are commercially available.

### 2.2 Synthesis of four-layer targets

**SCHEME 2:**
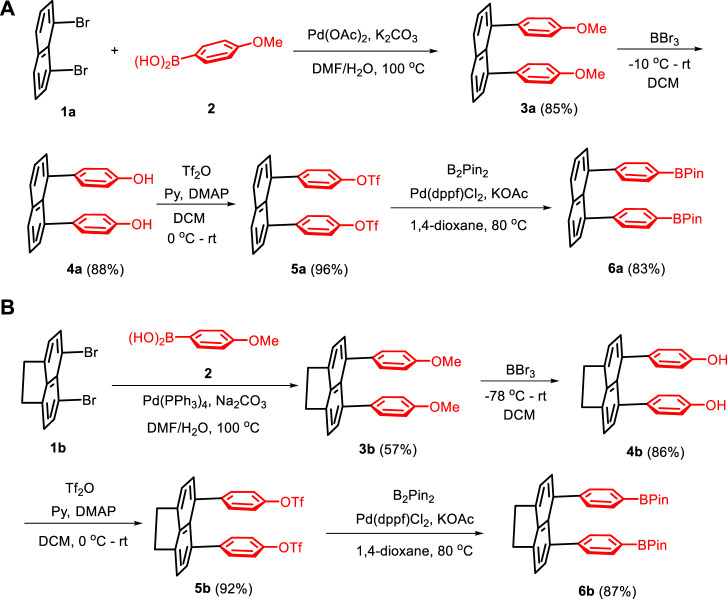
Synthesis of double-layer precursors **6a** and **6b**.

The assembly was represented by the synthesis of targets **6a** and **6b,** in which different conditions are explored for two steps to achieve higher efficiencies (Scheme 2). The synthesis of the building block 6a was started from Suzuki–Miyaura coupling of 1,8-dibromonaphthalene **1a** with (4-methoxyphenyl)boronic acid **2** by employing Pd(OAc)_2_ as a catalyst and K_2_CO_3_ as a base in the DMF/H_2_O co-solvent at 100°C, leading to the formation of 1,8-bis(4-methoxyphenyl)naphthalene **3a** in an 85% yield. The precursor 3a was transformed into 4,4'-(naphthalene-1,8-diyl)diphenol **4a** via demethylation in the presence of BBr_3_ in DCM by changing the temperature from −10°C to room temperature to afford an 88% yield. The precursor **4a** was allowed to react with excess trifluoromethanesulfonic anhydride (Tf_2_O) by using pyridine and 4-dimethylaminopyridine (DMAP) as bases to yield naphthalene-1,8-diylbis (4,1-phenylene) bis(trifluoromethanesulfonate) **5a** in a 96% yield. The reaction between **5a** and bis(pinacolato)diboron (B_2_Pin_2_) in *in situ* catalytic systems involve using KOAc as a base additive and (1,1-bis(diphenylphosphino)ferrocene) dichloropalladium (II) as a catalyst in 1,4-dioxane at 80°C to afford 1,8-bis(4-(4,4,5,5-tetramethyl-1,3,2-dioxaborolan-2-yl)phenyl)naphthalene **6a** as double-layer reactants (Scheme 2A). The synthesis of the building block of 5,6-bis(4-(4,4,5,5-tetramethyl-1,3,2-dioxaborolan-2-yl)phenyl)-1,2-dihydroacenaphthylene **6b** was also started from Suzuki–Miyaura coupling by treating 5,6-dibromo-1,2-dihydroacenaphthylene **1b** with (4-methoxyphenyl)boronic acid 2 by using Pd(PPh_3_)_4_ as the catalyst and Na_2_CO_3_ as the base in DMF/H_2_O as a mixed solvent at 100°C, to yield 5,6-bis(4-methoxyphenyl)-1,2-dihydroacenaphthylene **3b** in a 57% yield. The precursor **3b** was converted into 4,4'-(1,2-dihydroacenaphthylene-5,6-diyl)diphenol **4b** via demethylation in the presence of BBr_3_ in DCM by gradually changing temperature from −78°C to room temperature to afford an 86% yield. The two steps shown in Scheme 2B were performed under the same conditions as the aforementioned synthetic route to yield (1,2-dihydroacenaphthylene-5,6-diyl)bis (4,1-phenylene) bis(trifluoromethanesulfonate) **5b** and 5,6-bis(4-(4,4,5,5-tetramethyl-1,3,2-dioxaborolan-2-yl)phenyl)-1,2-dihydroacenaphthylene **6b** chemical yields of 92% and 87%, respectively.

The synthesis of another key precursor is represented by the generation of (1-bromonaphthalen-2-yl)diarylphosphine oxides **7** by the following literature procedures (Wu et al., 2021a). It was started with the protection of 1-bromo-2-naphthol with Tf_2_O to yield 1-bromonaphthalen-2-yl trifluoromethanesulfonate in the presence of pyridine. The second step was conducted through the C-P coupling with diaryl phosphine oxide by taking advantage of Pd_2_ (dba)_3_ and 1,3-bis(diphenylphosphino)propane (DPPP) as the catalytic combination (Ji et al., 2020). In addition, substrates **7b**–**7o** were synthesized starting with the nucleophilic substitution of diethyl phosphite with arylmagnesium bromide to yield bisaryl phosphine oxides, followed by subjecting to the catalytic coupling with 1-bromonaphthalen-2-yl trifluoromethanesulfonate.

The final step was to assemble the four-layer targets by treating 1,8-bis(4-(4,4,5,5-tetramethyl-1,3,2-dioxaborolan-2-yl)phenyl)naphthalene **6a** with (1-bromonaphthalen-2-yl)diarylphosphine oxides **7a** in the presence of a Pd(PPh_3_)_4_ catalyst as the key step, delivering various four-layered folding phosphine oxides **8a** in good yields (Scheme 1). At this step, it is necessary to optimize the conditions since Suzuki–Miyaura coupling between the double-layer diboronic ester **6a** and bromide **7a** did not result in ideal yields under the aforementioned catalytic systems. Different catalysts, solvents, and bases were screened, and the results are shown in Table 1 (entries 1–9). In the beginning, the reaction of **6a** and **7a** in a 1:2.5 mol ratio was carried out in the presence of 10 mol% Pd(PPh_3_)_4_ and 3.0 equiv of K_2_CO_3_ in THF/H_2_O (5:1, v/v) at 90 °C for 48 h, and the desired [1 + 4+1] multilayered *3D* product **8a** was obtained in 86% yield through dual Suzuki–Miyaura couplings (entry 1). Other Pd catalysts, including Pd_2_ (dba)_3_, PdCl_2_, and Pd(OAc)_2_, were then examined together with K_2_CO_3_ in this transformation, but all yielded unsatisfactory results (entries 2–4). Similarly, experimentation with various solvent systems, such as toluene/H_2_O, DME/H_2_O, and 1,4-dioxane/H_2_O did not produce satisfactory results either (entries 5–7). We next attempted to optimize conditions by exploiting K_3_PO_4_ and Cs_2_CO_3_ as bases and found that both attempts did not show poor chemical yields of 37% and 40%, respectively (entries 8 and 9).

**TABLE 1:** Optimization of the reaction conditions^a^.

Entry	[Pd] cat	Base	Solvent	Product (%)^b^
1	Pd(PPh_3_)_4_	K_2_CO_3_	THF/H_2_O	86
2	Pd_2_ (dba)_3_	K_2_CO_3_	THF/H_2_O	51
3	PdCl_2_	K_2_CO_3_	THF/H_2_O	60
4	Pd(OAc)_2_	K_2_CO_3_	THF/H_2_O	67
5	Pd(PPh_3_)_4_	K_2_CO_3_	Toluene/H_2_O	ND
6	Pd(PPh_3_)_4_	K_2_CO_3_	DME/H_2_O	NR
7	Pd(PPh_3_)_4_	K_2_CO_3_	1,4-Dioxane/H_2_O	23
8	Pd(PPh_3_)_4_	K_3_PO_4_	THF/H_2_O	37
9	Pd(PPh_3_)_4_	Cs_2_CO_3_	THF/H_2_O	40

^a^ Reaction conditions: **6a** (0.1 mmol), **7a** (0.25 mmol), [Pd] cat. (10 mol%) and base (6.0 equiv), solvent/H_2_O = 5 mL/1 mL, 48 h, under Ar conditions.

^b^ Isolated yield based on **6a**.

Since there are two polar -POPh_2_ groups existing in the products, the purification of resulting crude products at this step can be readily obtained through the group-assisted purification (GAP) (Kaur et al., 2010; Kaur et al., 2011; An et al., 2015) chemistry/technology, eliminating the need for chromatography and recrystallization. The pure product **8a** and its derivatives **8b—8p** were conveniently obtained by simply washing the crude products with 95% EtOH, making this synthesis much greener and environmentally friendly.

**SCHEME 3:**
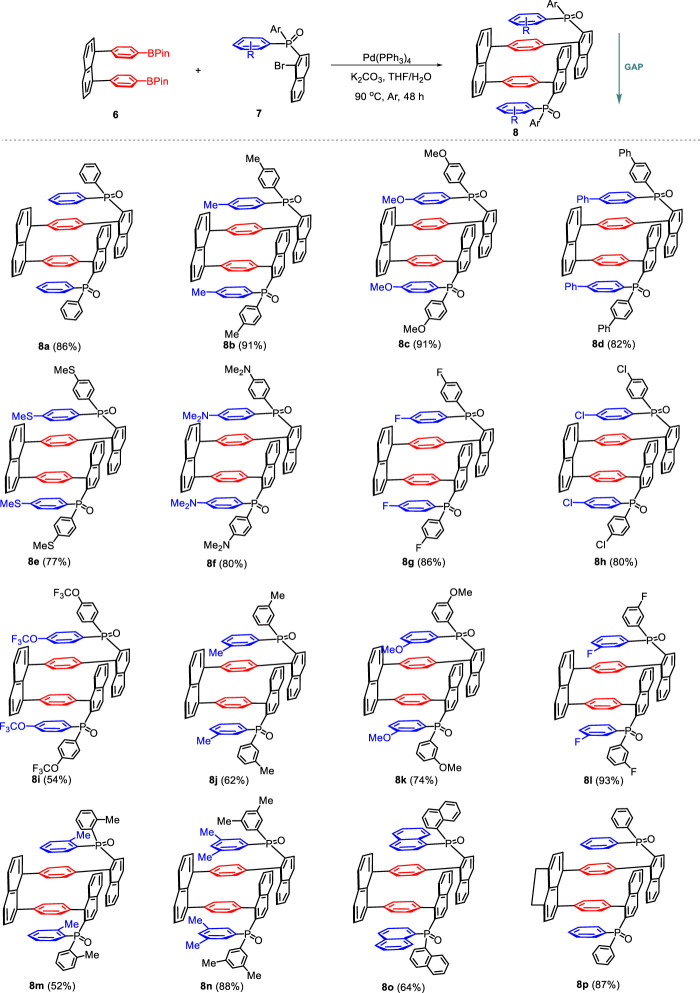
Substrate scope for forming products **8**.

Having established the optimal reaction conditions, we next investigated the scope of the double Suzuki–Miyaura cross-coupling reaction by using a variety of preformed 2-diarylphosphinyl-1-naphthyl bromide **7**. As shown in Scheme 3, the influence of substituents in the aryl moiety of **7** was first evaluated. The reactions of 2-diarylphosphinyl-1-naphthyl bromide **7** with either electron-rich groups (Me **7b**, OMe **7c**, Ph **7d**, SMe **7e,** and NMe2 **7f**) or electron-poor groups (F **7g**, Cl **7h**, and OCF_3_ **7i**) at the para position of the aryl moiety of **7** could tolerate this reaction system, leading to the corresponding products **8b–8i** in 54%–91% yield. Similarly, the meta-substituent of the aryl unit of **7** (Me **7j**, OMe **7k**, and F **7**l) still showed a high reactivity profile, providing access to the corresponding multilayered 3D products **7j–7**l in 62%–93% yield. It is noteworthy that the ortho-methyl substituted analog **7m** was a suitable surrogate for this coupling reaction, which could work smoothly to deliver the product **8m** in 52% yield. To our delight, both 3,5-dimethyl-substituted arylphosphine oxide **7n** and 1-naphthyl-substituted phosphine oxide **7o** were adopted to demonstrate the compatibility of this protocol and furnished the target products **8n**–**8o** in 88% and 64% yields, respectively. Furthermore, the dihydroacenaphthylene-derived double-layer diboronic ester **6b** was then allowed to react with 2-diphenylphosphinyl-1-naphthyl bromide **7a** under standard conditions. As anticipated, the reactions proceeded smoothly, enabling the Pd-catalyzed coupling to yield the corresponding product **8p** in 87% yield. The structures of all products were fully characterized by carbon and proton NMR spectroscopic and HRMS analyses.

**FIGURE 2:**
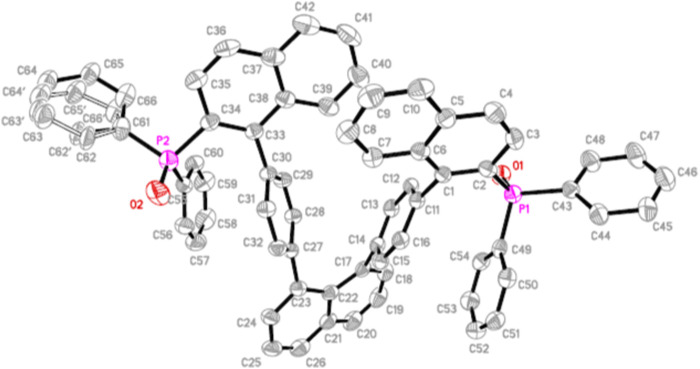
ORTEP drawing of **8a** (CCDC 2245941).

Furthermore, the resulting multilayer framework has been unambiguously assigned by X-ray structural analysis of one of the products, **8a** (Figure 2). This structure clearly presents two groups of nearly parallel units: four phenyl rings in the middle and two naphthyl rings at one end. These two planar units plus a single naphthyl ring anchor consist of a [1 + 4+2] multilayer framework, which added one more layer in the middle, as compared with our previous [1 + 3+1] type of multi-layer counterparts in the middle columns of their structures*.* It should be noted that our preliminary results on assembling a maximum of five-layer counterparts show promising results, leading to a [2 + 5+2] multilayer framework.

### 2.3 UV-vis absorption, PL, and AIE determinations

**FIGURE 3:**
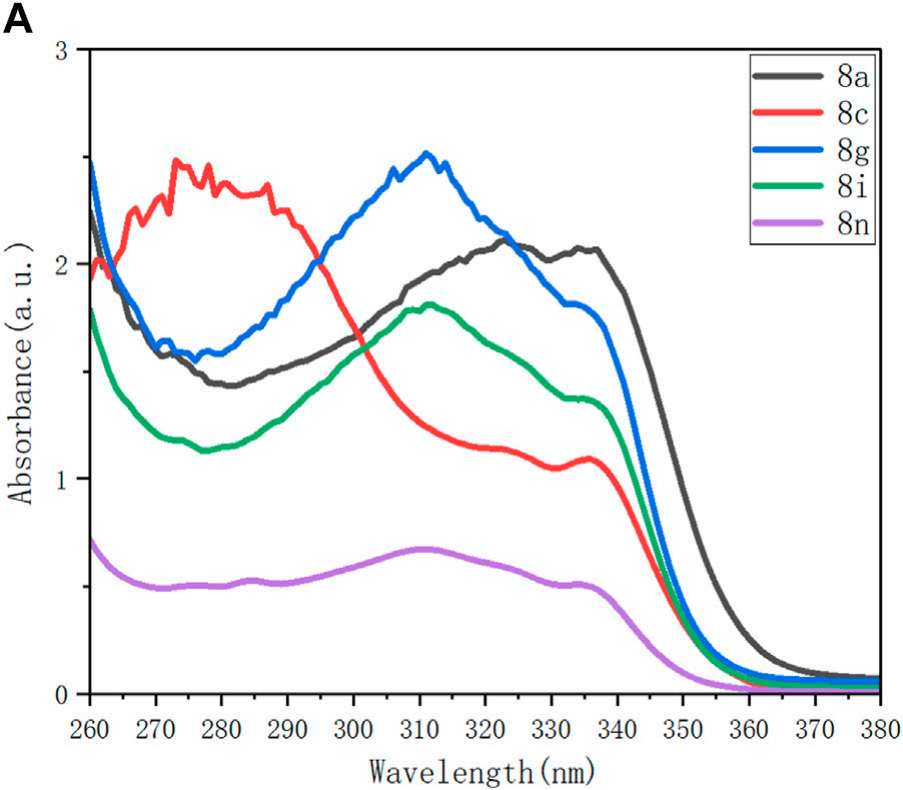
**(A)** UV–vis absorbance of **8a**, **8c**, **8g**, **8i,** and **8n** (0.1 mM) in THF; c = 0.1 mM. **(B)** Photoluminescence (PL) spectra of aforementioned five samples in THF; *c* = 0.1 mM; *λ*_ex_ (**8a**, **8c**, **8i,** and **8g**) **=** 344 nm; *λ*_ex_ (**8n**) **=** 352 nm.

**FIGURE 4:**
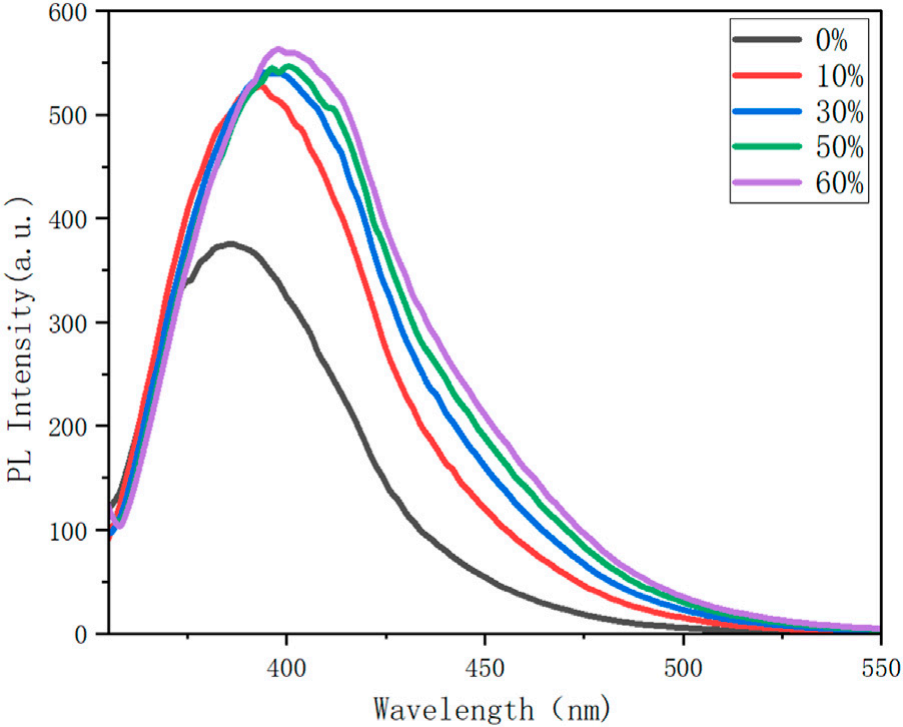
Photoluminescence (PL) spectra of **8n** in cosolvents of THF/H_2_O with different fractions (*f*_w_); *c* = 0.1 mM; *λ*_ex_ (**8n**) = 352 nm; inset: fluorescence photographs of **8n** in the THF/water system.

Among the products listed in Figure 3 and Figure 4, several representatives, **8a**, **8c**, **8g**, **8i,** and **8n,** were examined for their behaviors on UV–vis absorption, photoluminescence (PL), and aggregation-induced emission (AIE). As shown in Figure 3A, UV-vis absorption spectra were recorded for these compounds with the same concentration in THF (Figure 3A). The highest absorptions of four samples (**8a**, **8g**, **8i,** and **8n**) displayed wide absorption between 280 nm and 360 nm, except for molecule **8c**. The wide absorption of **8c** was observed to be between 260 and 210 nm; the highest position appeared at 280 nm, and the second highest position was around 340 nm.

The photoluminescence spectra of these compounds upon excitation exhibit bands at slightly different curves with regard to emission strengths and wavelengths (Figure 3B). Excitation wavelengths at 344 nm for **8a**, **8c**, **8i,** and **8g** and at 352 nm for **8n** were utilized for measuring their photoluminescence. As shown in the spectrum, **8c** displays its maximum emission at 400 nm. As compared with 8c, 8a displays its maximum emission at 430 nm shifted downfield and **8a**, **8g**, and **8i** at 344 nm shifted upfield, respectively. The PL performance seems complicated, being attributed to solvent–target interactions and electronic and conformational steric effects of aromatic rings attached to the phosphorus center. Compared with **8a,** which has no functional group on its two phenyl rings of the P=O center, the presence of both the electron-donating (OMe in **8c**) and electron-withdrawing groups (CF_3_O and F in **8g** and **8i**, respectively) and the steric effect (two methyl groups in **8n**) all resulted in upfield emission.

Fluorescence spectroscopic analysis was conducted using **8n** as a representative for aggregation-induced emission (AIE). As shown in Figure X, the water fractions (f_w_) were increased from 0% to 60%, resulting in a steady emission enhancement from 352 nm to 555 nm. This emission change is attributed to the intermolecular packing of the molecular matrix, indicating the existence of aggregation-induced emission (AIE) by this four-layer compound. Although an obvious GAP exists between emission in *f*_*w*_ = 0% (in pure THF) and the other four *fw* slots, the emission at the later four curves (10%–60%) does not display obvious differences.

As usual, the intermolecular aggregation largely suppresses the rotational motions of aromatic rings so that the exciton energy cannot be depleted by the radiation-less decay, thus making the present AIE observation possible. The intermolecular packing process would have an impact on the intramolecularly layered framework, but in a diluted environment, the movements between molecules would diminish as the poor solvent (water) became more prevalent. The intramolecular stacking would become more regular and predominantly controlled by suppressing the whole framework while water was added to the solvent mixture as soon as the acceptable saturation value of *f*_*w*_ = 60% was reached. The partial AIE activities could also exist because of this compressed packing model’s contribution to more efficient through-space interactions. This result is in accordance with our earlier research on multilayer molecules (Wu et al., 2021a; Wu et al., 2021b; Wang et al., 2022).

An interesting shape of emission appeared in pure and transparent THF, which indicates some degrees of molecular aggregation exist in this system. This observation would benefit organic synthesis during condition modifications by taking advantage of aggregates. It should be noted that THF is among the most common solvents in organic synthesis, particularly in asymmetric synthesis and catalysis. Our laboratory has recently proven that chiral aggregates can enhance asymmetric control and can even switch the stereo configuration of resulting chiral products (Rouh et al., 2022; Tang et al., 2023). Chiral aggregates were directly confirmed by AIE, AIP (aggregation-induced polarization) (Tang et al., 2022c; Tang et al., 2022d), and dynamic light scattering (DLS) experiments in THF-water and THF-ethanol co-solvents. Both stoichiometric and catalytic asymmetric reactions have been carried out successfully, defined as aggregation-induced asymmetric synthesis (AIAS) (Tang et al., 2023) and aggregation-induced asymmetric catalysis (AIAC) (Jin et al., 2022b).

## 3 Summary

In summary, a new [1 + 4+2] framework of multilayer targets has been successfully designed and synthesized. Starting from commercial starting materials, more than 40 steps were performed for generating 16 multilayer folding products bearing various phosphine oxides. The synthesis takes advantage of modified dual Suzuki–Miyaura cross-couplings and GAP chemistry/technology simply by washing with 95% EtOH without the use of chromatography and recrystallization. The structures were fully characterized by spectroscopic analysis and assigned by X-ray crystallographic determination. The physical properties of UV–vis absorption, photoluminescence (PL), and aggregation-induced emission (AIE) were studied for the resulting multilayer folding products. Further research on the asymmetric synthesis and catalysis for generating the chiral [1 + 4+2] framework of four-layer counterparts and its attachment onto orientational chirality (Jin et al., 2022b; Jin et al., 2022c) is currently being conducted in our laboratories, and the results will be reported in due course.

## Data Availability

The original contributions presented in the study are included in the article/Supplementary Material; further inquiries can be directed to the corresponding authors.
