# Supplementary material for: Four-layer folding framework: design, GAP synthesis, and aggregation-induced emission
Source: Front Chem. 2023 Aug 10;11:1259609. doi: 10.3389/fchem.2023.1259609 (PMC10450629; doi:10.3389/fchem.2023.1259609)
Supplement: Supplementary file 1 [file DataSheet1.PDF]

Supplementary Material for

Four-Layer Folding Framework: Design, GAP Synthesis and Aggregation-Induced Emission (AIE)

Sai Zhang[1,2], Daixiang Chen[1], Jia-Yin Wang[1]*, Shenghu Yan[1], Guigen Li[2]*

[1]Continuous Flow Engineering Laboratory of National Petroleum and Chemical Industry, Changzhou University, Changzhou, Jiangsu 213164, China,

[2] Department of Chemistry and Biochemistry, Texas Tech University, Lubbock, Texas 79409, USA

*** Correspondence:**

Jia-Yin Wang; wjychem@cczu.edu.cn

Guigen Li; guigen.li@ttu.edu

Table of Contents

S1. General Information 3

S2. General Procedure for the Synthesis of compound **6a**. 3

S3. General Procedure for the Synthesis of compound **6b** 4

S4. General Procedure for the Synthesis of compound **8**. 5

S5. Characterization Data of Compound **8** 5

S6.Copies of ^{1}H, ^{31}P, ^{19}F and ^{13}C NMR Spectra for Compounds **6** and **8** 14

S1. General Information

^{1}H NMR (^{13}C NMR) spectra were measured on a Bruker DPX 400 MHz spectrometer in $CDCl_3$ (DMSO-d_6) with chemical shift (δ) given in ppm relative to TMS as internal standard [(s = singlet, d = doublet, t = triplet, brs = broad singlet, m = multiplet), coupling constant (Hz)]. HRMS (ESI) was determined by using microTOF-QII HRMS/MS instrument (BRUKER). The melting points were measured with digital melting point detector. PE refers to petroleum ether (bp 60-90 °C), and EA refers to ethyl acetate. Other reagents, unless otherwise noted, were purchased from commercial vendors and used without further purification.

S2. General Procedure for the Synthesis of compound 6a.

Synthesis of 3a

Under Ar condition, to a stirred suspension of 1,8-dibromonaphthalene **1a** (20.0 mmol), (4-methoxyphenyl)boronic acid **2** (2.4 equiv., 48 mmol) and Cs_2CO_3(5 equiv., 100.0 mmol) in DMF/H_2O (95 mL:5 mL), was added the $Pd(OAc)_2$ (1 mol%, 0.2 mmol). The mixture was stirred at 100°C overnight. Diethyl ether (20 mL) and H_2O (20 mL) were then added and the aqueous phase was extracted with diethyl ether (2×10 mL). The combined organic layers were dried ($MgSO_4$), filtered, and concentrated under vacuum. The crude product was purified by flash chromatography (PE/EA = 95:5) on silica gel to give the corresponding product **3a** as a white solid.

Synthesis of 4a

Under argon atmosphere, to a stirring solution of **3a** (10.0 mmol) in DCM (40 mL) at -10 ^{o}C was added dropwise the solution of BBr_3 (1 M solution in DCM, 3.0 equiv.) Then, the reaction mixture was warmed to room temperature and stirred for 2 h. After the completion of the reaction which was indicated by TLC, water (50 mL) was added to the reaction mixture in an ice bath, and the aqueous layer was extracted three times with AcOEt. The combined organic layers were washed with brine, dried over anhydrous Na_2SO_4, and concentrated in vacuo to give a residue, which was purified by recrystallization to afford compound **4a** as white solid.

Synthesis of 5a

To a solution of **4a** (10.0 mmol) in 50.0 mL DCM was added pyridine (5.0 equiv.) and DMAP (20 mol %). Then, Tf_2O (3.0 equiv) dropwise at 0 ^{o}C. After that, the mixture was warmed to room temperature and stirred 12 h. The mixture was then quenched with HCl (1.0 mol/L) and extracted with DCM, washed with saturated $NaHCO_3$ and saturated brine. The organic layer was dried over anhydrous $MgSO_4$ and filtered. The filtrate was concentrated under reduced pressure, and the crude product was purified by flash column chromatography on silica gel (PE/EA = 50:1) to give the corresponding product **5a** as white solid.

Synthesis of 6a

Under Ar condition, $Pd(dppf)Cl_2$ (20 mol %), KOAc (5.0 equiv), **5a** (5.0 mmol), B_2pin_2 (3.0 equiv) and 1,4-dioxane (50 mL) were added to a Schlenk tube. The mixture was stirred at 80 ^{o}C for 8 hours.

The reaction mixture was cooled to room temperature and extracted with EA, washed with saturated brine. The organic layer was dried over anhydrous $MgSO_4$ and filtered. The filtrate was concentrated under reduced pressure, and the crude product was purified by column chromatography using PE/EtOAc (100:1) as an eluent to furnish pure product **6a** as white solid.

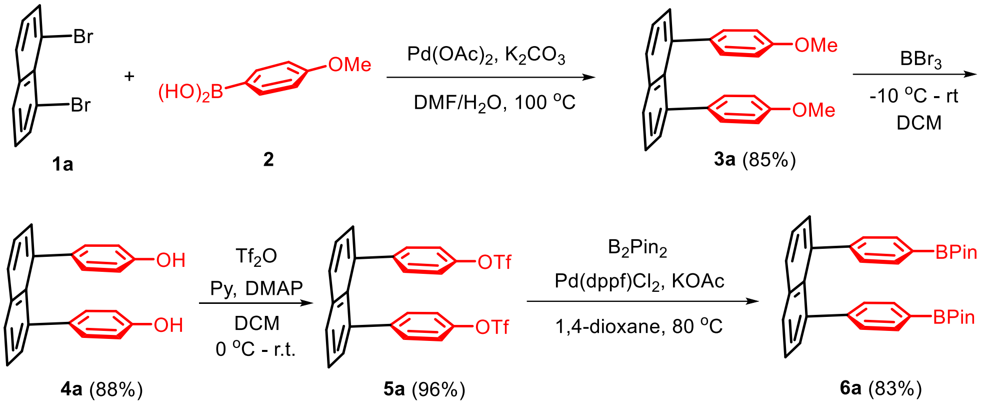

S3. General Procedure for the Synthesis of compound 6b

A round bottomed flask was charged with boronic acid **2** (3.0 equiv., 30.0 mmol), 5,6-dibromoacenaphthene **1b** (10.0 mmol), Na_2CO_3 (4.0 equiv., 40.0 mmol), DMF (70 mL) and H_2O (30 mL). The mixture was thoroughly degassed before the addition of $Pd(PPh_3)_4$ (5.0 mol%, 0.5 mmol) under argon. The resulting mixture was heated to 100 °C for 24 h before quenching with dilute hydrochloric acid. The mixture was extracted with dichloromethane and the organic phase washed with dilute hydrochloric acid, dried ($MgSO_4$) and evaporated to provide a crude oil. Purification by silica gel column chromatography(PE/EA = 50:1) gave compound **3b** as a colourless crystalline solid.

The next three steps are similar to the synthesis of compound **6a.**

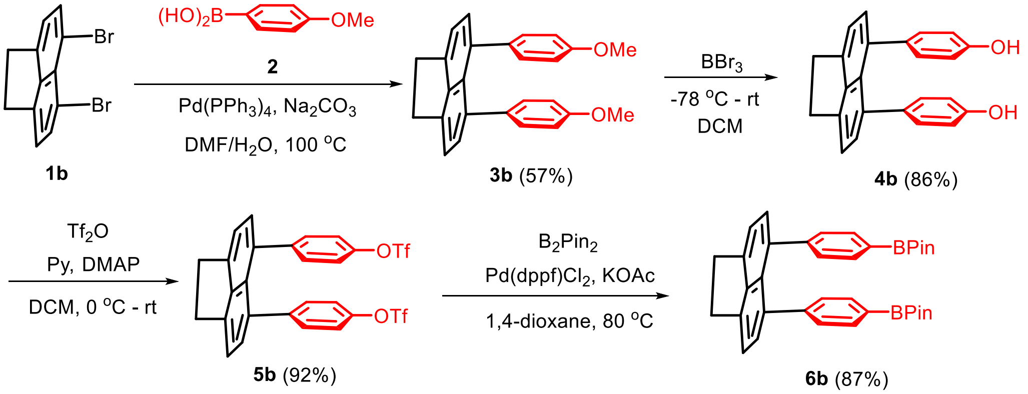

1,8-bis(4-(4,4,5,5-tetramethyl-1,3,2-dioxaborolan-2-yl)phenyl)naphthalene (6a)

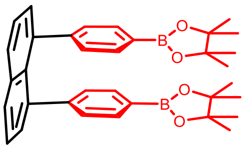

White solid, ^{1}H NMR (400 MHz, $CDCl_3$) (δ, ppm): 7.97-7.95 (m, 2H), 7.59-7.55 (m, 2H), 7.44-7.39 (m, 6H), 7.04 (d, *J* = 8.0 Hz, 4H), 1.33 (s, 24H); ^{13}C NMR (100 MHz, $CDCl_3$) (δ, ppm): 146.1, 140.5, 135.4, 133.8, 131.0, 129.1, 129.1, 128.5, 125.2, 83.4, 24.8; HRMS (ESI) m/z: calcd for $C_{34}H_{39}B_2O_4$ $[M+H]^+$ 533.3034, found 533.3006.

5,6-bis(4-(4,4,5,5-tetramethyl-1,3,2-dioxaborolan-2-yl)phenyl)-1,2-dihydroacenaphthylene (6b)

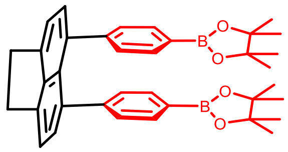

White solid, ^{1}H NMR (400 MHz, $CDCl_3$) (δ, ppm): 7.42-7.38 (m, 8H), 7.03 (d, *J* = 7.6 Hz, 4H), 3.51 (s, 4H), 1.33 (s, 24H); ^{13}C NMR (100 MHz, $CDCl_3$) (δ, ppm): 146.1, 145.3, 140.6, 136.2, 133.6, 132.4, 128.9, 119.3, 83.4, 30.2, 24.8. HRMS (ESI) m/z: calcd for $C_{36}H_{41}B_2O_4$ $[M+H]^+$ 559.3191, found 559.3178.

S4. General Procedure for the Synthesis of compound 8.

Example for the synthesis of **8a**:

Under Ar condition, to a stirred suspension of **6a** (0.1 mmol), **7a** (2.5 equiv., 0.25 mmol) and K_2CO_3(6.0 equiv., 0.6 mmol) in THF/H_2O (5 mL:1 mL), was added the $Pd(PPh_3)_3$ (10 mol%, 0.01 mmol). The mixture was stirred at 90 °C for 48 hours. ethyl acetate (5 mL) and H_2O (10 mL) were then added and the aqueous phase was extracted with ethyl acetate (2×10 mL). The combined organic layers were dried with $MgSO_4$, filtered, and concentrated under vacuum. The crude product was purified by washing with 95% EtOH to give the corresponding product **8a** as a white solid.

S5. Characterization Data of Compound 8

((Naphthalene-1,8-diylbis(4,1-phenylene))bis(naphthalene-1,2-diyl))bis(diphenylphosphine oxide) (8a)

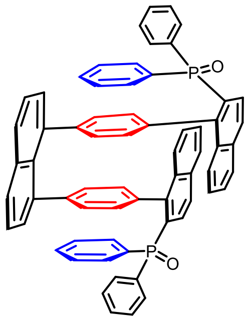

White solid could be obtained by washing the crude products with 95% EtOH; 86% yield. mp: 287-289 °C; ^{1}H NMR (400 MHz, $CDCl_3$) (δ, ppm): 8.01 (dd, *J* = 8.3, 1.3 Hz, 1H), 7.66-7.54 (m, 6H), 7.44-7.33 (m, 4H), 7.29-7.22 (m, 5H), 7.11 (d, *J* = 8.6 Hz, 1H), 7.01 (d, *J* = 7.7 Hz, 2H), 6.88-6.78

(m, 3H), 6.36-6.29 (m, 1H). ^{31}P NMR (162 MHz, $CDCl_3$) (δ, ppm): 26.78. ^{13}C NMR (100 MHz, $CDCl_3$) (δ, ppm): 146.8, 146.7, 142.5, 140.3, 135.5, 134.4, 134.3(4), 134.3(0), 134.2, 134.1, 133.3, 132.0, 131.9, 131.4, 131.3, 131.2, 131.0, 130.8, 130.7, 129.6, 129.2, 128.5(3), 128.5(5), 128.4, 128.3, 128.2, 128.0, 127.8, 127.7, 127.1, 126.7, 126.6, 126.5, 125.7, 125.1. HRMS (ESI) m/z: calcd for $C_{66}H_{47}O_2P_2$ $[M+H]^+$ 933.3051, found 933.3035.

((Naphthalene-1,8-diylbis(4,1-phenylene))bis(naphthalene-1,2-diyl))bis(di-p-tolylphosphine oxide) (8b)

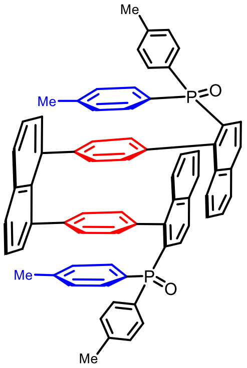

White solid could be obtained by washing the crude products with 95% EtOH; 91% yield. mp: 299-302 °C; ^{1}H NMR (400 MHz, $CDCl_3$) (δ, ppm): 8.01 (dd, J = 8.2, 1.3 Hz, 1H), 7.68-7.57 (m, 2H), 7.49-7.36 (m, 7H), 7.23 (dd, J = 7.0, 1.3 Hz, 1H), 7.12 (d, J = 8.6 Hz, 1H), 7.07-7.00 (m, 5H), 6.87-6.77 (m, 3H), 6.30 (ddd, J = 8.4, 6.8, 1.2 Hz, 1H), 2.31 (s, 6H). ^{31}P NMR (162 MHz, $CDCl_3$) (δ, ppm): 27.39. ^{13}C NMR (100 MHz, $CDCl_3$) (δ, ppm): 146.5, 146.4, 142.5, 140.9(9), 140.9(6), 140.4, 135.5, 135.1(2), 135.1(5), 134.4, 134.3(4), 134.3(0), 134.3(8), 132.0, 131.9, 131.4, 131.3(3), 131.3(0), 131.2, 131.1, 130.3, 129.8, 129.1, 128.8, 128.7, 128.6, 128.5, 128.4, 128.3, 128.1, 127.7, 127.0, 126.7, 126.6, 126.4, 125.6, 125.0, 21.5. HRMS (ESI) m/z: calcd for $C_{70}H_{55}O_2P_2$ $[M+H]^+$ 989.3677, found 989.3657.

((Naphthalene-1,8-diylbis(4,1-phenylene))bis(naphthalene-1,2-diyl))bis(bis(4-methoxyphenyl)phosphine oxide) (8c)

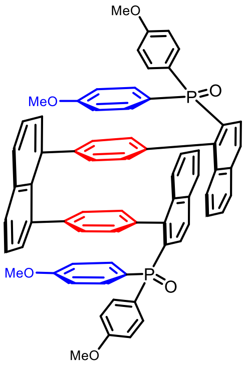

White solid could be obtained by washing the crude products with 95% EtOH; 91% yield. mp: 289-291 °C; ^{1}H NMR (400 MHz, $CDCl_3$) (δ, ppm): 8.00 (dd, *J* = 8.2, 1.3 Hz, 1H), 7.63 (dt, *J* = 8.7, 6.6 Hz, 2H), 7.49-7.40 (m, 7H), 7.32 (dd, *J* = 7.0, 1.3 Hz, 1H), 7.09 (d, *J* = 8.6 Hz, 1H), 7.02-6.97 (m, 2H), 6.85-6.82 (m, 2H), 6.76 (dd, *J* = 8.8, 2.3 Hz, 4H), 6.30 (ddd, *J* = 8.4, 6.8, 1.3 Hz, 1H), 3.73 (s, 6H). 31P NMR (162 MHz, $CDCl_3$) (δ, ppm): 27.09. ^{13}C NMR (100 MHz, $CDCl_3$) (δ, ppm): 161.5(0), 161.5(7), 146.3, 146.2, 142.4, 140.5, 135.5, 135.1(2), 135.1(5), 134.5(4), 134.5(8), 134.3, 134.1, 134.0, 133.1, 133.0, 132.1, 132.0, 131.1, 131.0, 130.5, 130.2, 129.3, 129.2, 128.4, 128.3, 128.1, 127.8, 127.0, 126.7, 126.6, 126.5, 125.9, 125.6, 125.1, 124.8, 113.6, 113.4, 55.2. HRMS (ESI) m/z: calcd for $C_{70}H_{55}O_6P_2$ $[M+H]^+$ 1053.3474, found 1053.3461.

((Naphthalene-1,8-diylbis(4,1-phenylene))bis(naphthalene-1,2-diyl))bis(di([1,1'-biphenyl]-4-yl)phosphine oxide) (8d)

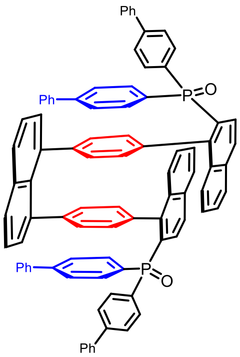

White solid could be obtained by washing the crude products with 95% EtOH; 82% yield. mp: 307-309 °C; ^{1}H NMR (400 MHz, $CDCl_3$) (δ, ppm): 7.99 (d, *J* = 8.1 Hz, 1H), 7.67 (dd, *J* = 11.0, 8.0 Hz, 5H), 7.57 (dd, *J* = 12.2, 8.6 Hz, 1H), 7.50-7.33 (m, 16H), 7.23 (d, *J* = 6.9 Hz, 1H), 7.07 (dd, *J* = 28.2, 8.2 Hz, 3H), 6.88 (t, *J* = 7.5 Hz, 1H), 6.80 (d, *J* = 7.8 Hz, 2H), 6.35 (t, *J* = 7.7 Hz, 1H). ^{31}P NMR (162 MHz, $CDCl_3$) (δ, ppm): 26.49. ^{13}C NMR (100 MHz, $CDCl_3$) (δ, ppm): 143.5, 142.7, 140.3, 140.1, 135.6, 134.5, 133.0, 131.9, 131.8, 131.2(2), 131.2(7), 128.8, 128.4, 128.1, 127.9, 127.8, 127.3, 127.1, 126.8, 126.7, 126.6, 125.8, 125.2. HRMS (ESI) m/z: calcd for $C_{90}H_{63}O_2P_2$ $[M+H]^+$ 1237.4303, found 1237.4276.

((Naphthalene-1,8-diylbis(4,1-phenylene))bis(naphthalene-1,2-diyl))bis(bis(4-(methylthio)phenyl)phosphine oxide) (8e)

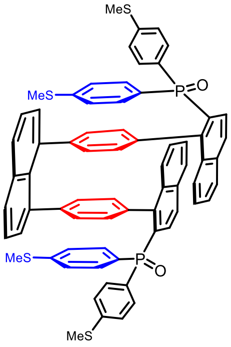

White solid could be obtained by washing the crude products with 95% EtOH; 77% yield. mp: 326-328 °C; ^{1}H NMR (400 MHz, $CDCl_3$) (δ, ppm): 8.01 (dd, *J* = 8.2, 1.3 Hz, 1H), 7.68-7.59 (m, 2H), 7.48-7.32 (m, 7H), 7.07 (ddd, *J* = 21.5, 19.2, 8.2 Hz, 7H), 6.84 (d, *J* = 7.7 Hz, 3H), 6.29 (ddd, *J* = 8.3, 6.7, 1.2 Hz, 1H), 2.40 (s, 6H). ^{31}P NMR (162 MHz, $CDCl_3$) (δ, ppm): 26.57. ^{13}C NMR (100 MHz, $CDCl_3$) δ 146.5(4), 146.5(5), 142.8, 142.7, 140.2, 135.5, 134.3(4), 134.3(9), 131.9, 131.8, 131.6, 131.5, 131.4, 131.1, 130.1, 129.5, 129.1, 129.0, 128.6, 128.5, 128.4, 128.3, 128.2, 127.6, 127.1, 126.7, 126.6, 125.7, 125.2, 124.9, 124.8, 14.7. HRMS (ESI) m/z: calcd for $C_{70}H_{55}O_2P_2S_4$ $[M+H]^+$ 1117.2560, found 1117.2548.

((Naphthalene-1,8-diylbis(4,1-phenylene))bis(naphthalene-1,2-diyl))bis(bis(4-(dimethylamino)phenyl)phosphine oxide) (8f)

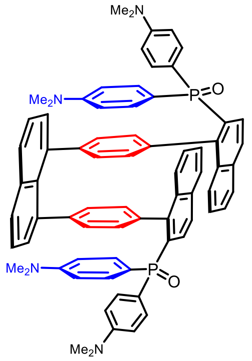

White solid could be obtained by washing the crude products with 95% EtOH; 80% yield. mp: 339-341 °C; ^{1}H NMR (400 MHz, $CDCl_3$) (δ, ppm): 7.96 (dd, *J* = 8.2, 1.4 Hz, 1H), 7.63-7.55 (m, 3H), 7.42-7.30 (m, 6H), 7.07 (d, *J* = 8.6 Hz, 1H), 6.98 (d, *J* = 7.7 Hz, 2H), 6.81 (d, *J* = 7.8 Hz, 3H), 6.51 (dd, *J* = 8.8, 2.3 Hz, 4H), 6.32-6.23 (m, 1H), 2.88 (s, 12H). ^{31}P NMR (162 MHz, $CDCl_3$) (δ, ppm): 28.49. ^{13}C NMR (100 MHz, $CDCl_3$) (δ, ppm): 151.6, 146.0, 145.9, 142.1, 141.1, 135.4, 134.9(4), 134.9(8), 134.2, 132.6, 132.5, 132.2, 132.1, 131.0, 130.8, 129.6, 128.8, 128.7, 128.3, 128.0, 126.7, 126.6, 126.2, 126.1, 125.5, 125.0, 111.1, 111.0, 40.0. HRMS (ESI) m/z: calcd for $C_{74}H_{67}N_4O_2P_2$ $[M+H]^+$ 1105.4739, found 1105.4738.

((Naphthalene-1,8-diylbis(4,1-phenylene))bis(naphthalene-1,2-diyl))bis(bis(4-fluorophenyl)phosphine oxide) (8g)

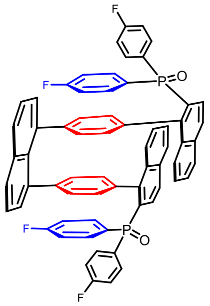

White solid could be obtained by washing the crude products with 95% EtOH; 86% yield. mp: 277-279 °C; ^{1}H NMR (400 MHz, $CDCl_3$) (δ, ppm): 8.04 (dd, *J* = 8.3, 1.3 Hz, 1H), 7.68 (dd, *J* = 8.2, 7.0 Hz, 1H), 7.63-7.50 (m, 5H), 7.40-7.34 (m, 2H), 7.26 (dd, *J* = 7.0, 1.3 Hz, 1H), 7.13 (d, *J* = 8.7 Hz, 1H), 7.04-6.93 (m, 6H), 6.89-6.81 (m, 3H), 6.31 (ddd, *J* = 8.3, 6.8, 1.3 Hz, 1H). ^{31}P NMR (162 MHz, $CDCl_3$) (δ, ppm): 25.72. ^{13}C NMR (100 MHz, $CDCl_3$) (δ, ppm): 165.7, 163.2, 163.1, 146.7, 146.6, 142.8, 139.8, 135.6, 135.1(2), 135.1(6), 134.4(1), 134.4(9), 134.1(1), 134.1(5), 133.8, 133.7(9), 133.7(7), 133.6, 131.9, 131.8, 131.3, 131.2, 130.1(0), 130.1(6), 129.0(3), 129.0(0), 129.0(6), 128.9, 128.8, 128.6, 128.0(3), 128.0(7), 127.9, 127.5, 127.3, 126.9, 126.8, 126.7, 125.8, 125.3, 115.5, 115.4, 115.3, 115.2. ^{19}F NMR (376 MHz, $CDCl_3$) (δ, ppm): -107.89. HRMS (ESI) m/z: calcd for $C_{66}H_{43}F_4O_2P_2$ $[M+H]^+$ 1005.2674, found 1005.2619.

((Naphthalene-1,8-diylbis(4,1-phenylene))bis(naphthalene-1,2-diyl))bis(bis(4-chlorophenyl)phosphine oxide) (8h)

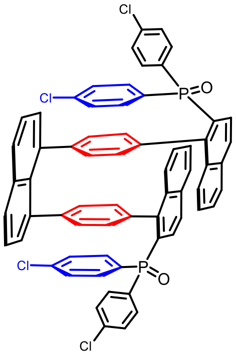

White solid could be obtained by washing the crude products with 95% EtOH; 80% yield. mp: 327-329 °C; ^{1}H NMR (400 MHz, $CDCl_3$) (δ, ppm): 8.06 (dd, *J* = 8.2, 1.4 Hz, 1H), 7.70 (dd, *J* = 8.2, 7.1 Hz, 1H), 7.60 (dd, *J* = 8.7, 2.6 Hz, 1H), 7.50-7.44 (m, 4H), 7.40-7.34 (m, 3H), 7.25 (ddd, *J* = 8.5, 6.5, 1.9 Hz, 5H), 7.15 (d, *J* = 8.7 Hz, 1H), 7.05-7.00 (m, 2H), 6.87-6.84 (m, 2H), 6.30 (ddd, *J* = 8.4, 6.8, 1.3 Hz, 1H). ^{31}P NMR (162 MHz, $CDCl_3$) (δ, ppm): 25.56. ^{13}C NMR (100 MHz, $CDCl_3$) (δ, ppm): 146.7(4), 146.7(5), 143.0, 139.5, 137.5, 137.4, 135.6, 134.4(2), 134.4(0), 134.0(3), 134.0(7), 132.7, 132.6, 131.8, 131.7, 131.6, 131.2, 129.0, 128.7, 128.6, 128.4, 128.3(3), 128.3(8), 128.0, 127.9, 127.4, 127.0, 126.8, 125.8, 125.3. HRMS (ESI) m/z: calcd for $C_{66}H_{43}Cl_4O_2P_2$ $[M+H]^+$ 1071.1463, found 1071.1422.

((Naphthalene-1,8-diylbis(4,1-phenylene))bis(naphthalene-1,2-diyl))bis(bis(4-(trifluoromethoxy)phenyl)phosphine oxide) (8i)

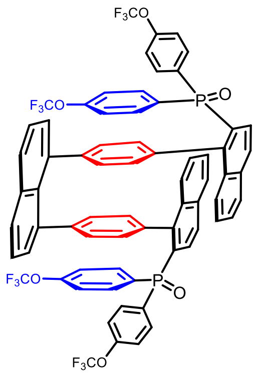

White solid could be obtained by washing the crude products with 95% EtOH; 54% yield. mp: 253-255 °C; ^{1}H NMR (400 MHz, $CDCl_3$) (δ, ppm): 8.04 (d, *J* = 8.2 Hz, 1H), 7.62 (ddd, *J* = 17.5, 13.1, 8.1 Hz, 6H), 7.43-7.32 (m, 2H), 7.24 (d, *J* = 7.0 Hz, 1H), 7.12 (d, *J* = 8.4 Hz, 5H), 7.04 (d, *J* = 7.8 Hz, 2H), 6.87 (d, *J* = 7.6 Hz, 3H), 6.34 (t, *J* = 7.7 Hz, 1H). ^{31}P NMR (162 MHz, $CDCl_3$) (δ, ppm): 24.88. ^{13}C NMR (100 MHz, $CDCl_3$) (δ, ppm): 151.3(7), 151.3(5), 151.2, 147.0, 146.9, 143.1, 139.6, 135.6, 134.5(9), 134.5(7), 133.9, 133.8, 133.3, 133.2, 132.6, 131.9, 131.8, 131.5, 131.3, 128.9, 128.8, 128.5(2), 128.5(6), 127.9, 127.7, 127.4, 127.0, 126.9, 125.8, 125.2, 124.1, 121.5, 120.1, 119.9, 119.0, 116.4. ^{19}F NMR (376 MHz, $CDCl_3$) (δ, ppm): -57.48. HRMS (ESI) m/z: calcd for $C_{70}H_{43}F_{12}O_6P_2$ $[M+H]^+$ 1269.2343, found 1269.2275.

((Naphthalene-1,8-diylbis(4,1-phenylene))bis(naphthalene-1,2-diyl))bis(di-m-tolylphosphine oxide) (8j)

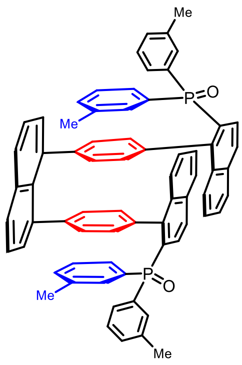

White solid could be obtained by washing the crude products with 95% EtOH; 62% yield. mp: 267-269 °C; ^{1}H NMR (400 MHz, $CDCl_3$) (δ, ppm): 8.00 (d, *J* = 8.1 Hz, 1H), 7.64-7.58 (m, 2H), 7.47-7.37 (m, 5H), 7.33-7.30 (m, 1H), 7.26 (d, *J* = 7.1 Hz, 1H), 7.17-7.09 (m, 5H), 7.04 (d, *J* = 7.7 Hz, 2H), 6.83 (d, *J* = 7.7 Hz, 3H), 6.33 (t, *J* = 7.7 Hz, 1H), 2.24 (s, 6H). ^{31}P NMR (162 MHz, $CDCl_3$) (δ, ppm): 27.36. ^{13}C NMR (100 MHz, $CDCl_3$) (δ, ppm): 146.6, 146.5, 142.4, 140.3, 137.7, 137.6, 135.5, 135.1(2), 135.1(5), 134.3(4), 134.3(2), 134.3, 134.1, 133.1, 132.0, 131.9, 131.5(0), 131.5(7), 131.3, 131.0, 130.5, 129.6, 129.2, 128.6, 128.5, 128.4, 128.3, 128.1, 128.0, 127.8(2), 127.8(5), 127.7, 127.1, 126.7, 126.6, 126.5, 125.7, 125.1, 21.4. HRMS (ESI) m/z: calcd for $C_{70}H_{55}O_2P_2$ $[M+H]^+$ 989.3677, found 989.3626.

((Naphthalene-1,8-diylbis(4,1-phenylene))bis(naphthalene-1,2-diyl))bis(bis(3-methoxyphenyl)phosphine oxide) (8k)

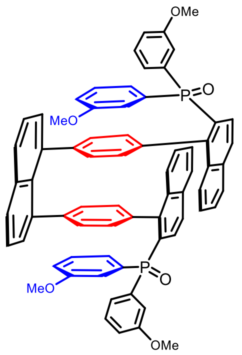

White solid could be obtained by washing the crude products with 95% EtOH; 74% yield. mp: 249-251 °C; ^{1}H NMR (400 MHz, $CDCl_3$) (δ, ppm): 8.01 (dd, *J* = 8.3, 1.3 Hz, 1H), 7.67-7.58 (m, 2H), 7.44-7.36 (m, 3H), 7.32 (dd, *J* = 7.1, 1.3 Hz, 1H), 7.21-7.11 (m, 5H), 7.05 (dd, *J* = 12.0, 4.5 Hz, 3H), 6.91 (dd, *J* = 8.2, 2.6 Hz, 2H), 6.87-6.79 (m, 3H), 6.28 (ddd, *J* = 8.3, 6.8, 1.3 Hz, 1H), 3.66 (s, 6H). ^{31}P NMR (162 MHz, $CDCl_3$) (δ, ppm): 26.86. ^{13}C NMR (100 MHz, $CDCl_3$) (δ, ppm): 159.1, 159.0, 146.8(4), 146.8(5), 142.5, 140.1, 135.6, 135.4, 135.1, 134.5, 134.4, 134.1, 134.1, 132.0, 131.9, 131.4, 131.3, 129.6, 129.1, 129.0, 128.6, 128.4, 128.3, 128.2, 127.6, 127.1, 126.7, 126.6, 126.5, 125.6, 125.1, 123.6, 123.5, 117.2, 116.3, 116.2, 55.2. HRMS (ESI) m/z: calcd for $C_{70}H_{55}O_6P_2$ $[M+H]^+$ 1053.3474, found 1053.3414.

((Naphthalene-1,8-diylbis(4,1-phenylene))bis(naphthalene-1,2-diyl))bis(bis(3-fluorophenyl)phosphine oxide) (8l)

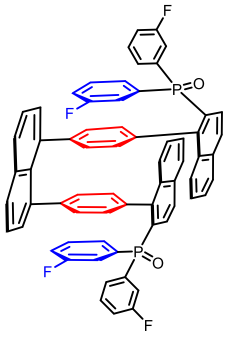

White solid could be obtained by washing the crude products with 95% EtOH; 93% yield. mp: 285-287 °C; ^{1}H NMR (400 MHz, $CDCl_3$) (δ, ppm): 8.02 (d, *J* = 8.1 Hz, 1H), 7.68-7.61 (m, 2H), 7.41 (d, *J* = 8.7 Hz, 2H), 7.31 (dtd, *J* = 23.0, 10.0, 9.4, 3.3 Hz, 7H), 7.16-7.03 (m, 5H), 6.88 (dd, *J* = 7.8, 3.8 Hz, 3H), 6.36 (t, *J* = 7.7 Hz, 1H). ^{31}P NMR (162 MHz, $CDCl_3$) δ 24.96, 24.92, 24.89. ^{13}C NMR (100 MHz, $CDCl_3$) (δ, ppm): 163.5, 163.3, 161.0, 160.8, 147.0, 146.9, 143.1, 139.8, 136.6(4), 136.6(9), 135.6(1), 135.6, 135.5(6), 134.5(2), 134.5(0), 133.9(3), 133.9(7), 131.9, 131.8, 131.3, 131.1, 130.0(2), 130.0(5), 129.9, 129.8, 129.1, 128.6(2), 128.6(0), 128.3, 128.0, 127.8, 127.6, 127.4, 127.2, 127.0, 126.9(3), 126.9(9), 125.9, 125.2, 118.5, 118.4, 118.3(8), 118.3(5), 118.2, 118.1. HRMS (ESI) m/z: calcd for $C_{66}H_{43}F_4O_2P_2$ $[M+H]^+$ 1005.2674, found 1005.2608.

((Naphthalene-1,8-diylbis(4,1-phenylene))bis(naphthalene-1,2-diyl))bis(di-o-tolylphosphine oxide) (8m)

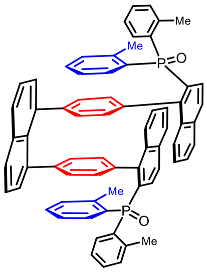

White solid could be obtained by washing the crude products with 95% EtOH; 52% yield. mp: 217-219 °C; ^{1}H NMR (400 MHz, $CDCl_3$) (δ, ppm): 7.99 (d, *J* = 8.1 Hz, 1H), 7.75-7.67 (m, 2H), 7.62 (t, *J* = 7.6 Hz, 1H), 7.47 (d, *J* = 8.2 Hz, 1H), 7.32 (t, *J* = 6.8 Hz, 3H), 7.22-7.13 (m, 3H), 7.08-6.96 (m, 4H), 6.94-6.82 (m, 3H), 6.77 (d, *J* = 7.8 Hz, 2H), 6.32 (t, *J* = 7.7 Hz, 1H), 2.40 (s, 6H). ^{31}P NMR (162 MHz, $CDCl_3$) (δ, ppm): 34.27. ^{13}C NMR (101 MHz, $CDCl_3$) δ 145.8(4), 145.8(6), 142.8, 142.4, 140.3, 135.5, 134.3, 134.3, 134.2, 134.1, 133.2, 133.1, 132.5, 132.4, 131.6, 131.5, 131.4, 131.3, 131.2(1), 131.2(8), 130.4, 130.2, 129.7, 129.2, 128.7, 128.5, 128.3, 128.1, 127.9, 127.8, 127.2, 127.1, 126.9, 126.8, 125.3, 125.2, 125.1, 125.0, 22.3(1), 22.3(7). HRMS (ESI) m/z: calcd for $C_{70}H_{55}O_2P_2$ $[M+H]^+$ 989.3677, found 989.3618.

((Naphthalene-1,8-diylbis(4,1-phenylene))bis(naphthalene-1,2-diyl))bis(bis(3,5-dimethylphenyl)phosphine oxide) (8n)

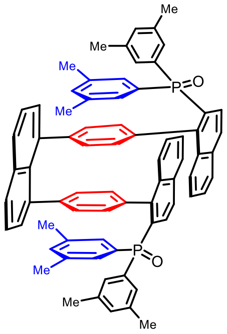

White solid could be obtained by washing the crude products with 95% EtOH; 88% yield. mp: 191-193 °C; ^{1}H NMR (400 MHz, $CDCl_3$) (δ, ppm): 7.99 (dd, *J* = 8.2, 1.4 Hz, 1H), 7.64-7.57 (m, 2H), 7.52-7.31 (m, 3H), 7.24-7.06 (m, 8H), 6.96 (s, 2H), 6.84 (d, *J* = 7.4 Hz, 3H), 6.35 (ddd, *J* = 8.3, 6.8, 1.3 Hz, 1H), 2.20 (s, 12H). ^{31}P NMR (162 MHz, $CDCl_3$) (δ, ppm): 28.06. ^{13}C NMR (100 MHz, $CDCl_3$) δ 146.5, 146.4, 142.4, 140.3, 137.5, 137.4, 135.4, 135.2, 134.4(4), 134.4(9), 134.3(2), 134.3(0), 133.9, 132.9, 132.5, 132.4, 132.0, 131.9, 131.3, 131.0, 129.6, 129.2, 129.1, 129.0, 128.6, 128.5, 128.4, 128.2, 127.8, 127.0, 126.7, 126.6, 126.4, 125.7, 125.0, 21.3. HRMS (ESI) m/z: calcd for $C_{74}H_{63}O_2P_2$ $[M+H]^+$ 1045.4303, found 1045.4236.

((Naphthalene-1,8-diylbis(4,1-phenylene))bis(naphthalene-1,2-diyl))bis(di(naphthalen-1-yl)phosphine oxide) (8o)

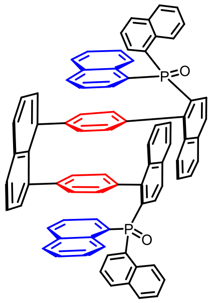

White solid could be obtained by washing the crude products with 95% EtOH; 64% yield. mp: 228-230 °C; ^{1}H NMR (400 MHz, $CDCl_3$) (δ, ppm): 8.69-7.83 (m, 8H), 7.81-7.29 (m, 10H), 7.22-6.85 (m, 6H), 6.46 (d, J = 8.0 Hz, 2H), 6.28 (t, J = 7.7 Hz, 1H). ^{31}P NMR (162 MHz, $CDCl_3$) (δ, ppm): 33.67. ^{13}C NMR (100 MHz, $CDCl_3$) (δ, ppm): 145.8, 145.7, 142.1, 140.1, 135.5, 134.4(0), 134.4(8), 134.0, 133.9, 133.7, 133.6, 132.5, 132.4, 132.3, 131.1, 130.0, 129.7, 129.2, 129.1, 128.7, 128.6, 128.4, 128.2, 128.1, 127.9, 127.5, 127.4, 127.1, 126.9, 126.1, 125.3, 125.0, 124.5, 124.3. HRMS (ESI) m/z: calcd for $C_{82}H_{55}O_2P_2$ $[M+H]^+$ 1133.3677, found 1133.3616.

(((1,2-dihydroacenaphthylene-5,6-diyl)bis(4,1-phenylene))bis(naphthalene-1,2-diyl))bis(diphenylphosphine oxide) (8p)

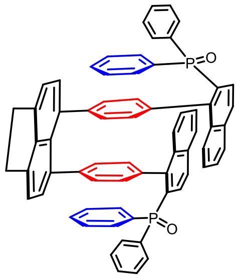

White solid could be obtained by washing the crude products with 95% EtOH; 64% yield. mp: 230-232 °C; ^{1}H NMR (400 MHz, $CDCl_3$) (δ, ppm):7.60-7.52 (m, 5H), 7.49-7.39 (m, 4H), 7.36-7.26 (m, 5H), 7.22 (d, J = 7.0 Hz, 1H), 7.14 (d, J = 8.6 Hz, 1H), 6.94 (d, J = 8.0 Hz, 2H), 6.83-6.73 (m, 3H), 6.29 (ddd, J = 8.4, 6.8, 1.3 Hz, 1H), 3.58 (s, 2H). ^{31}P NMR (162 MHz, $CDCl_3$) (δ, ppm): 26.69. ^{13}C NMR (100 MHz, $CDCl_3$) (δ, ppm): 146.9, 146.8, 146.2, 141.4, 140.6, 135.9, 134.4, 134.2, 134.0, 133.9, 133.4, 132.3, 132.0, 131.9, 131.5, 131.4, 131.1, 130.7(0), 130.7(7), 129.4, 128.4, 128.2, 128.1, 128.0, 127.9, 127.5, 127.0(4), 127.0(8), 126.7, 126.6, 126.4, 125.5, 119.3, 30.2. HRMS (ESI) m/z: calcd for $C_{68}H_{49}O_2P_2$ $[M+H]^+$ 959.3208, found 959.3140.

S6.Copies of 1H, 31P and 13C NMR Spectra for Compounds 6 and 8

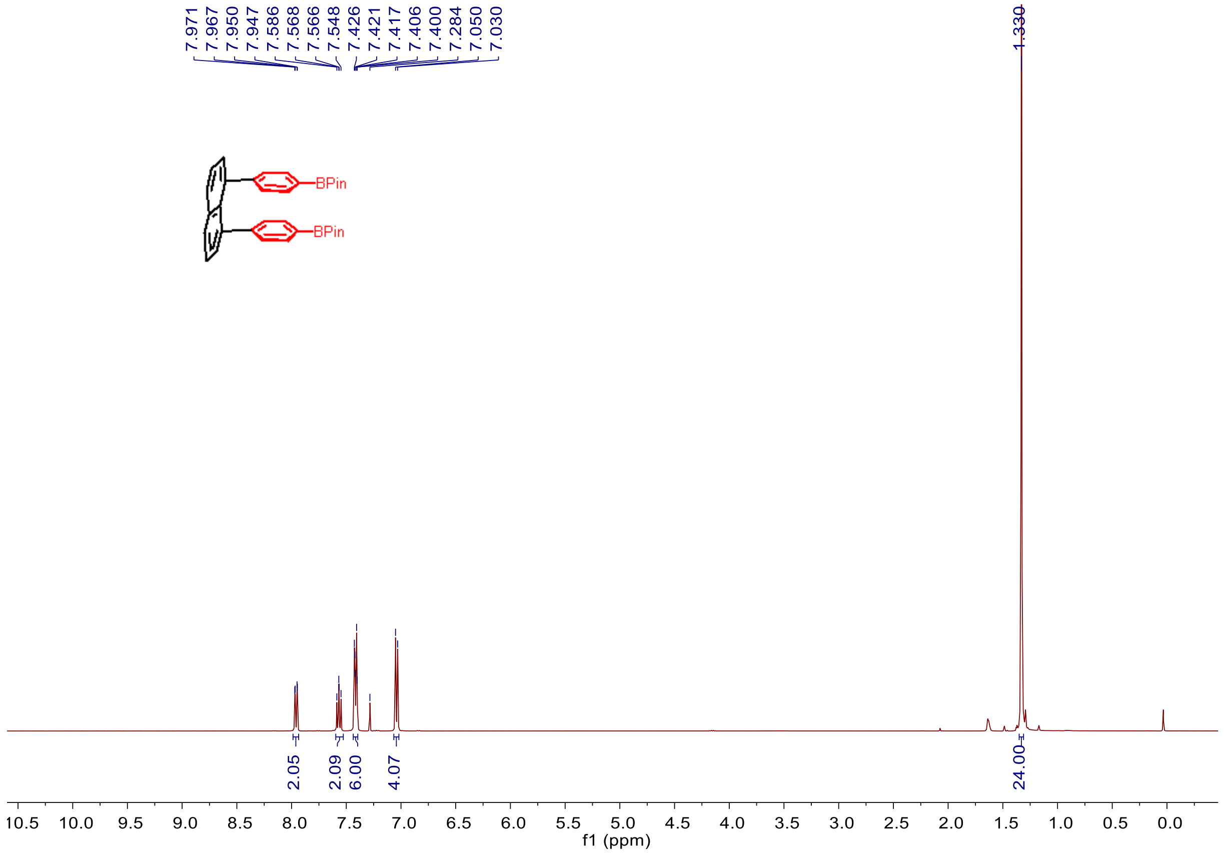

^{1}H NMR Spectrum of Compound 6a

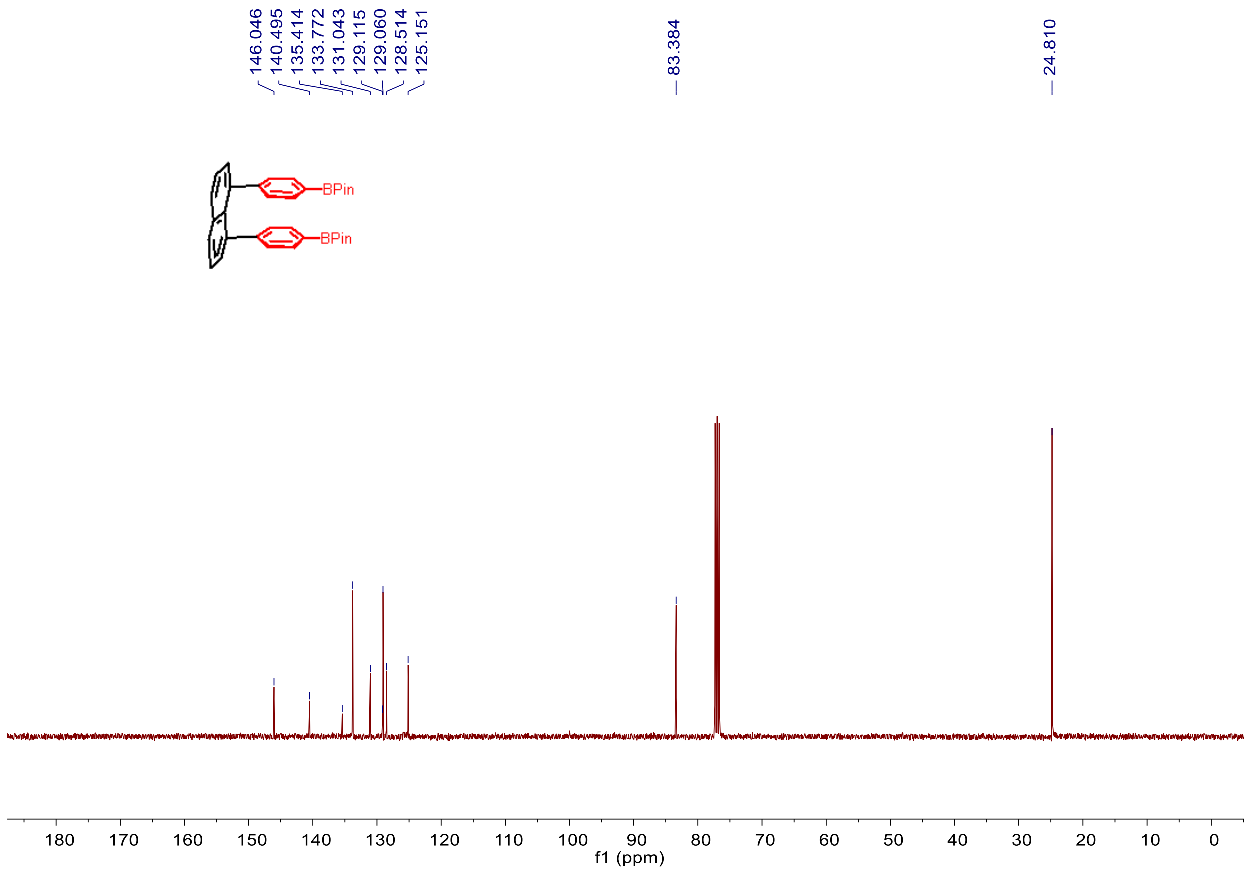

^{13}C NMR Spectrum of Compound 6a

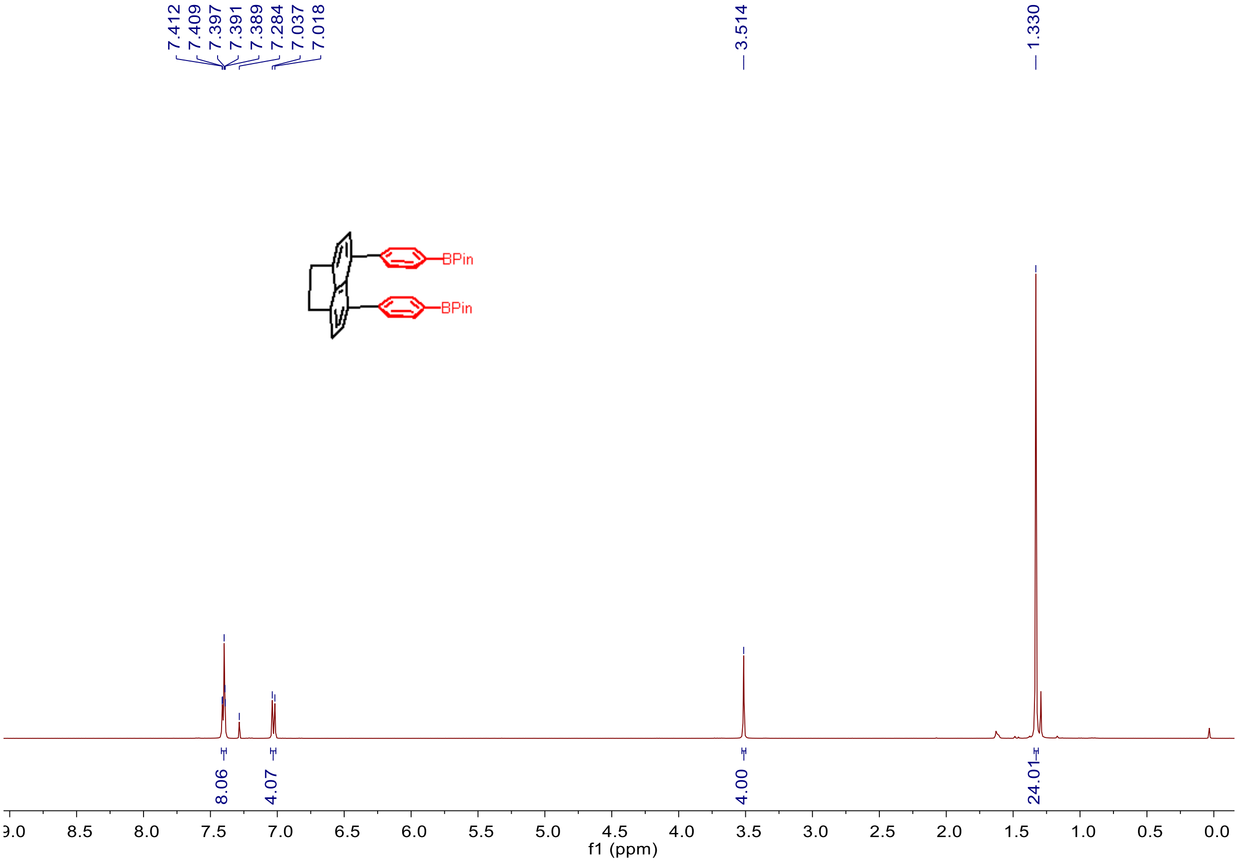

^{1}H NMR Spectrum of Compound 6b

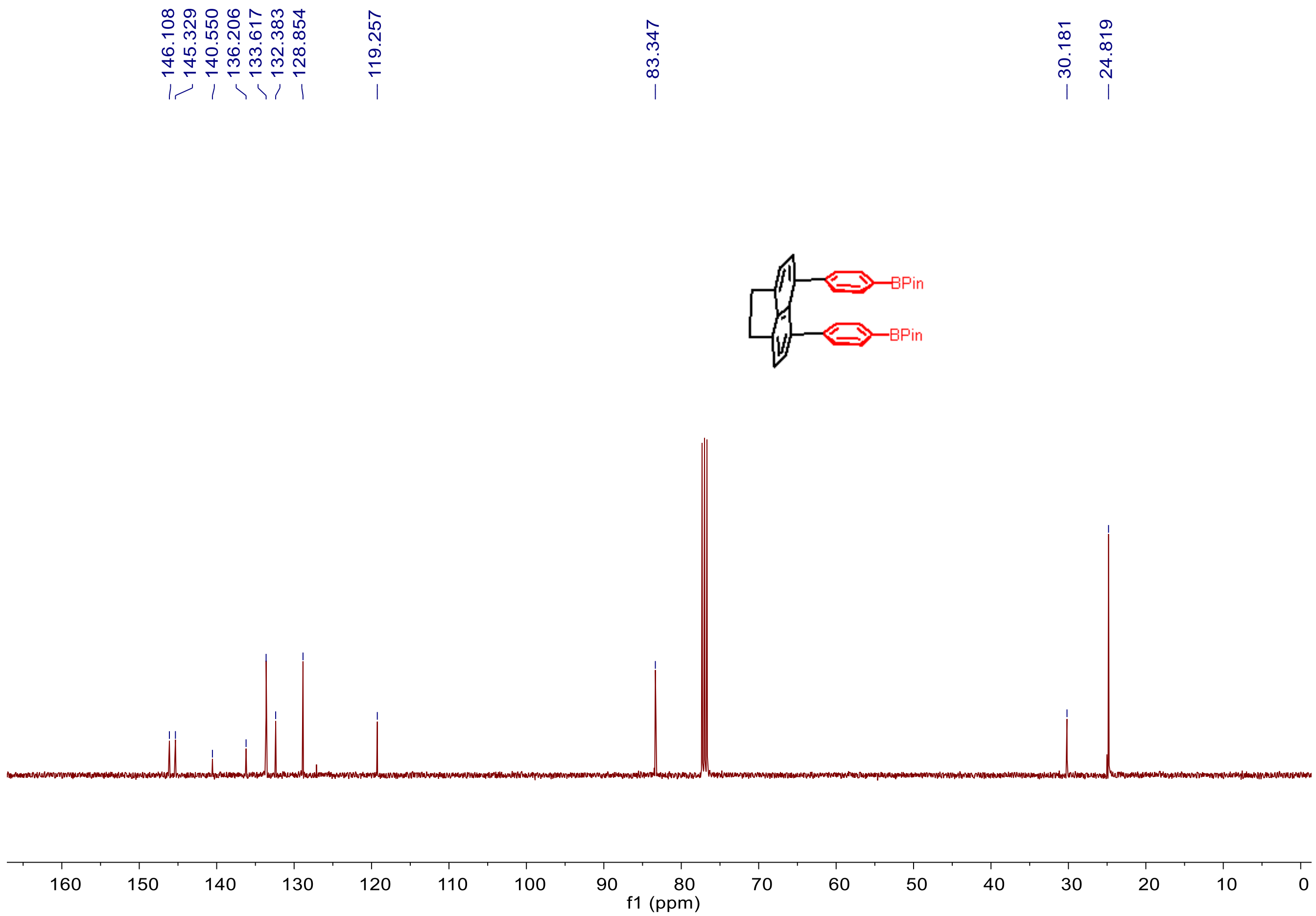

^{13}C NMR Spectrum of Compound 6b

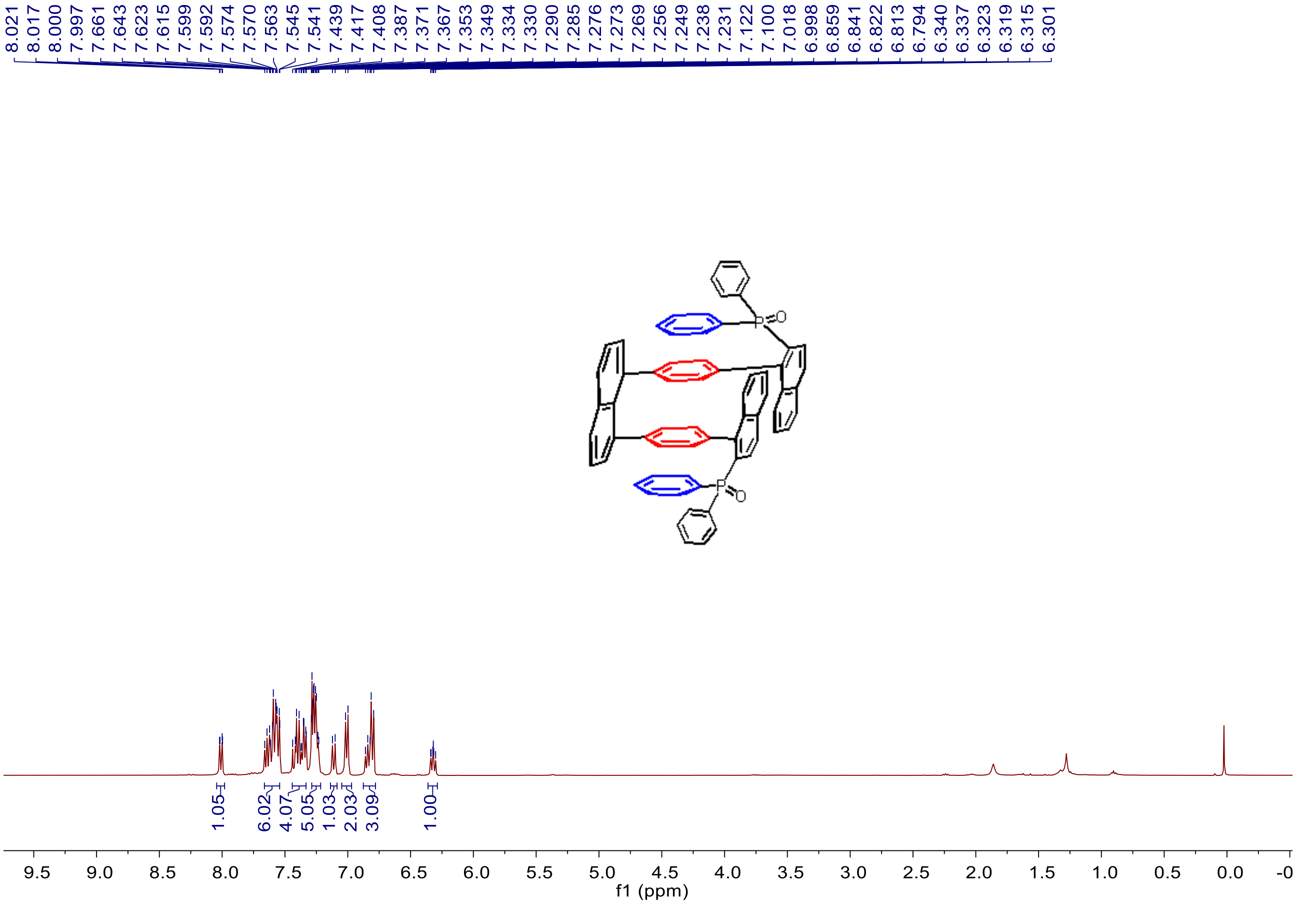

^{1}H NMR Spectrum of Compound 8a

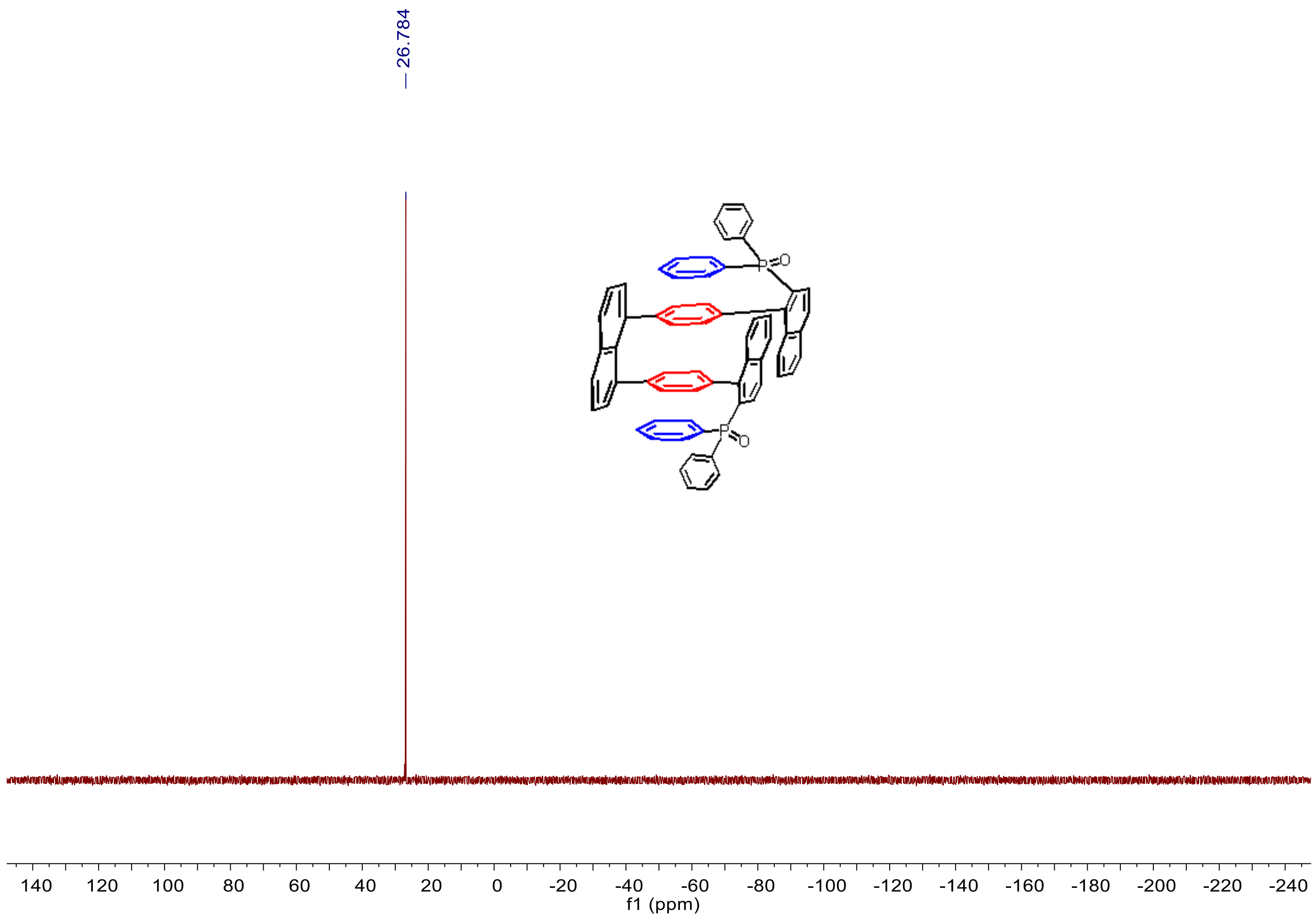

^{31}P NMR Spectrum of Compound 8a

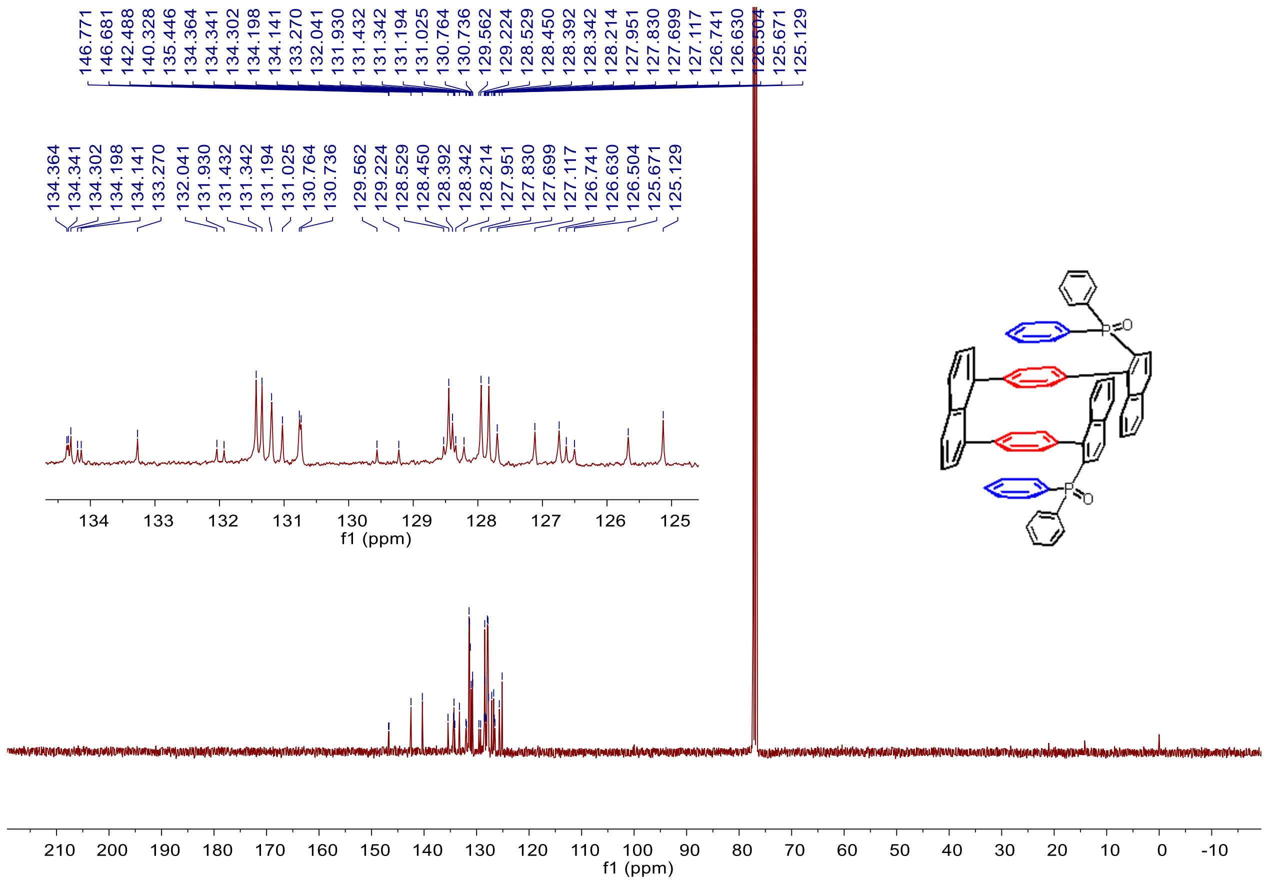

^{13}C NMR Spectrum of Compound 8a

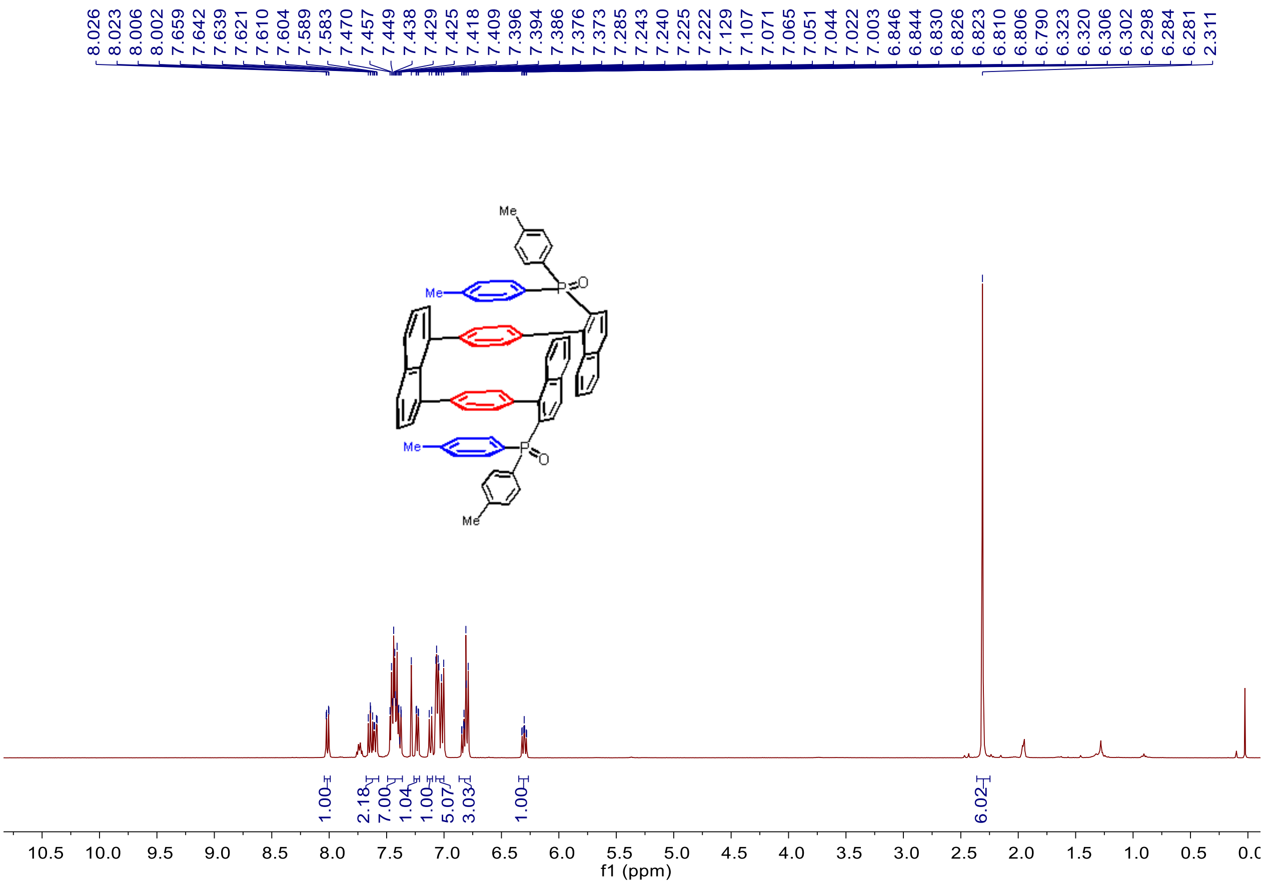

^{1}H NMR Spectrum of Compound 8b

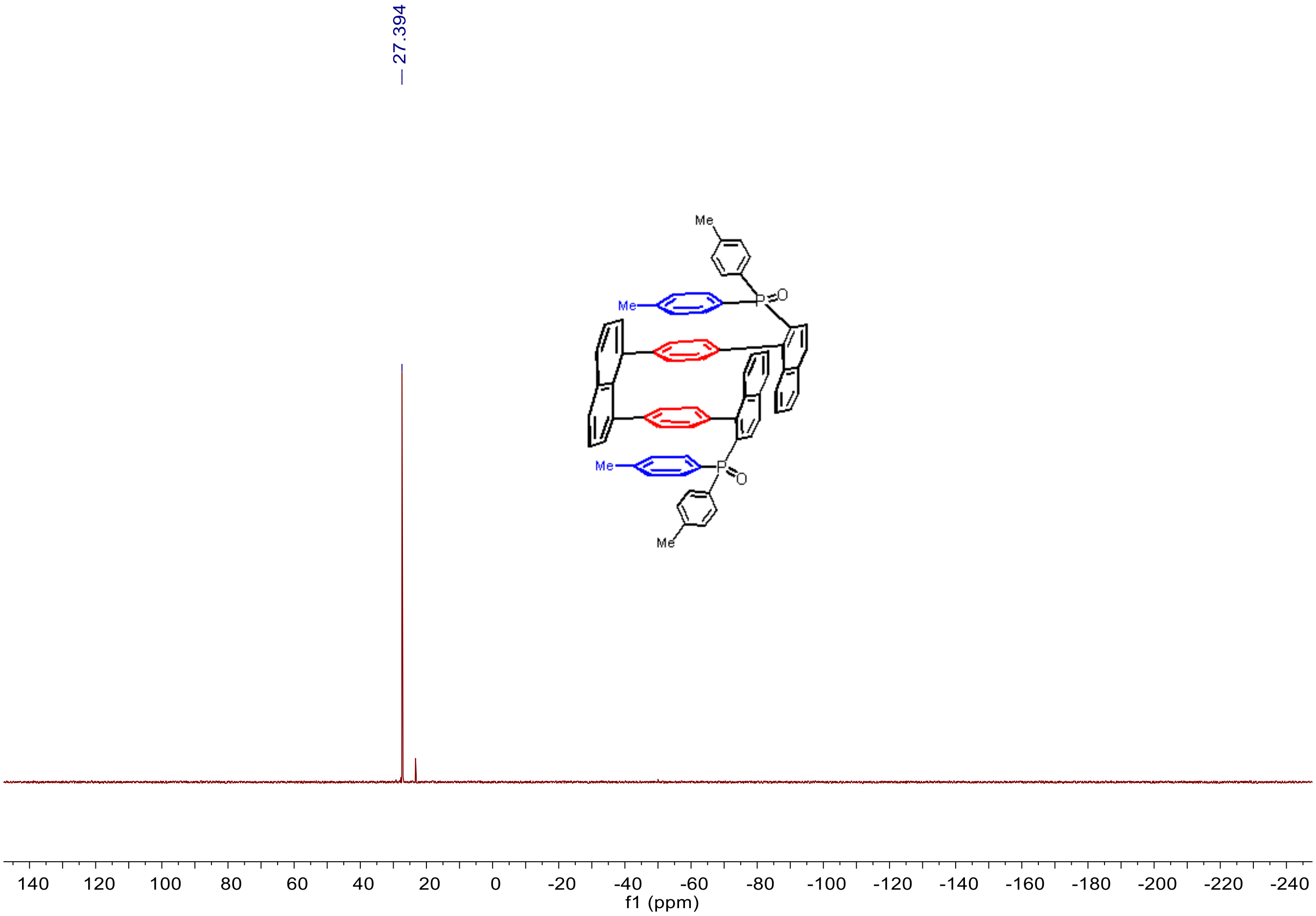

^{31}P NMR Spectrum of Compound 8b

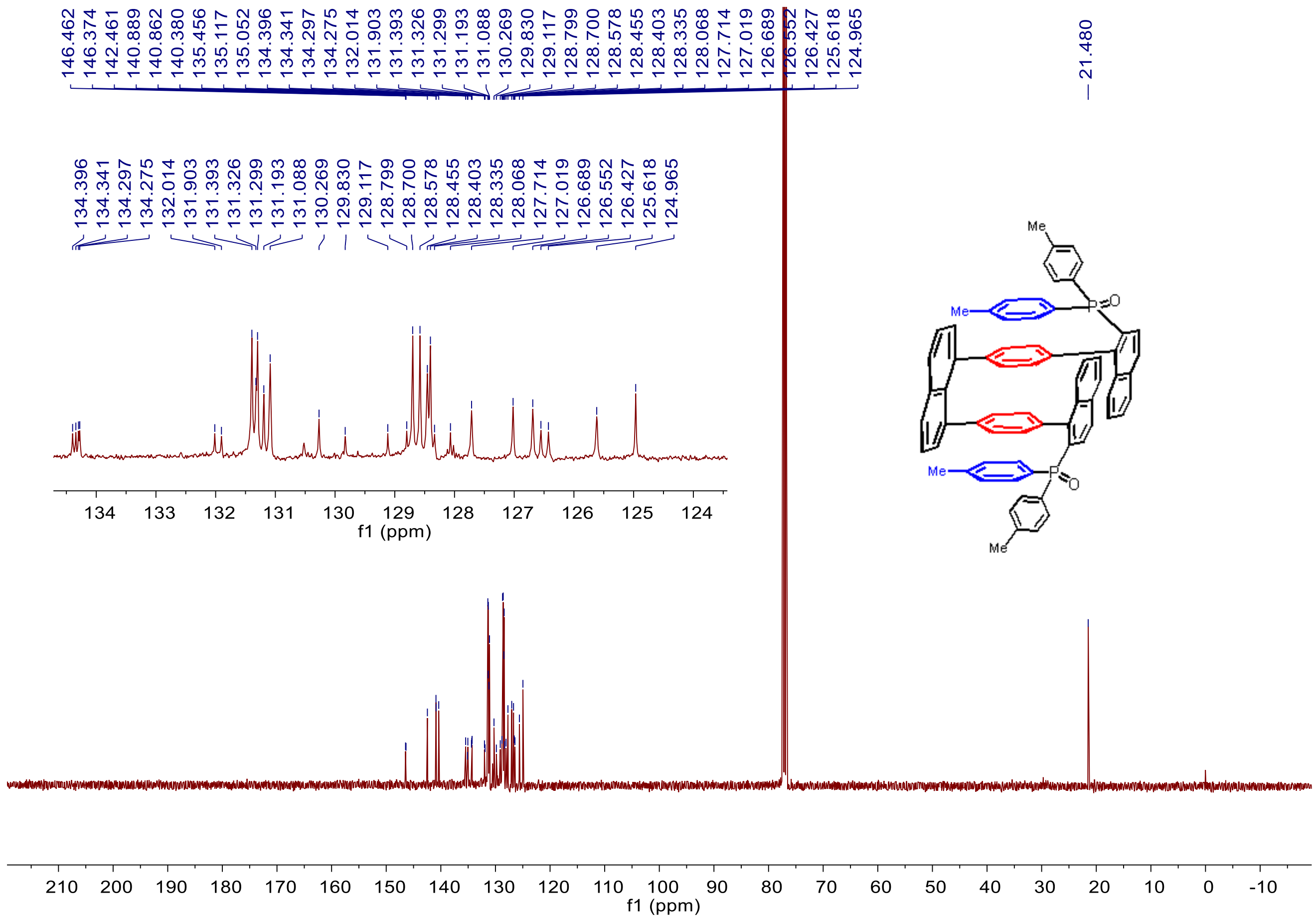

^{13}C NMR Spectrum of Compound 8b

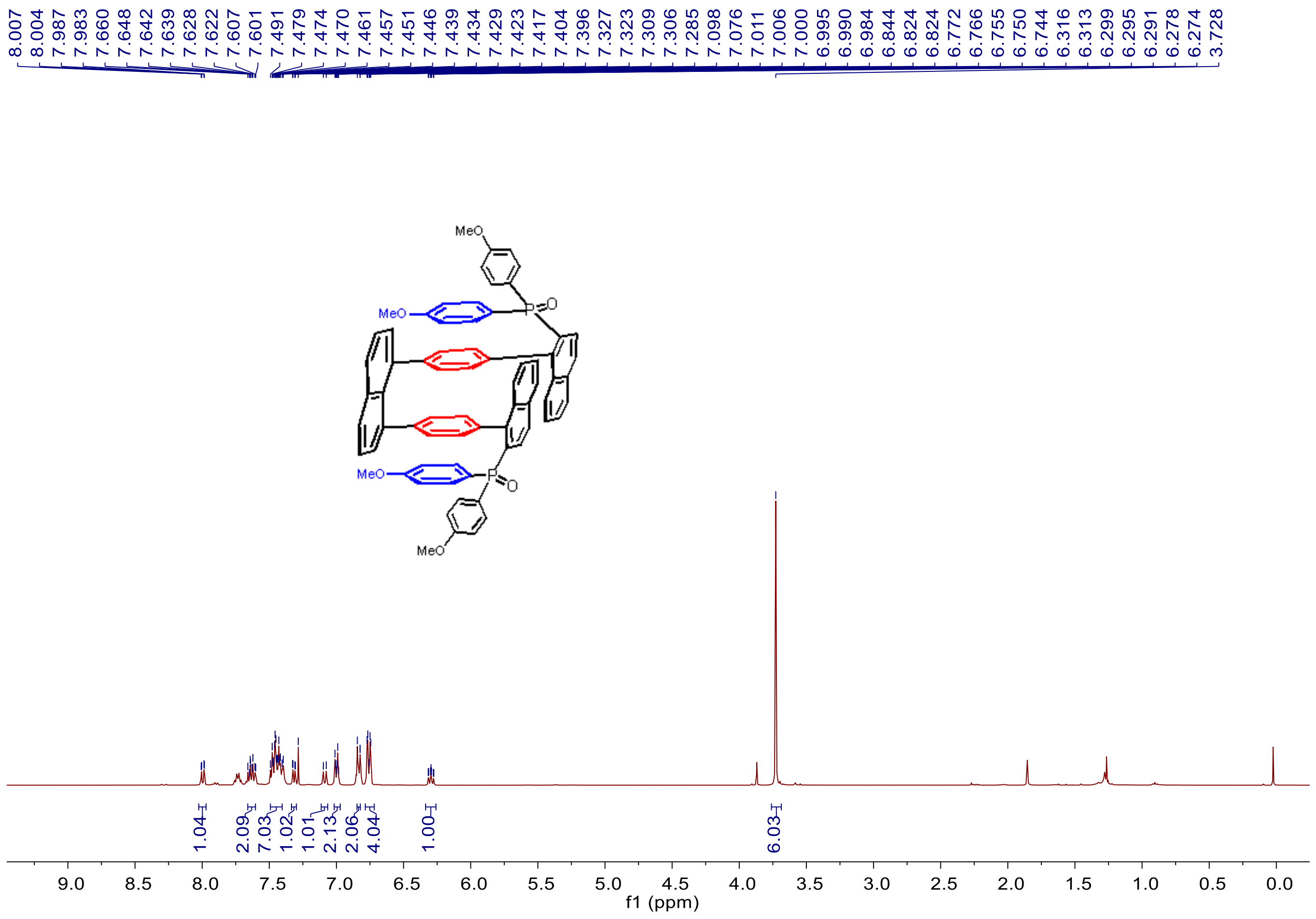

^{1}H NMR Spectrum of Compound 8c

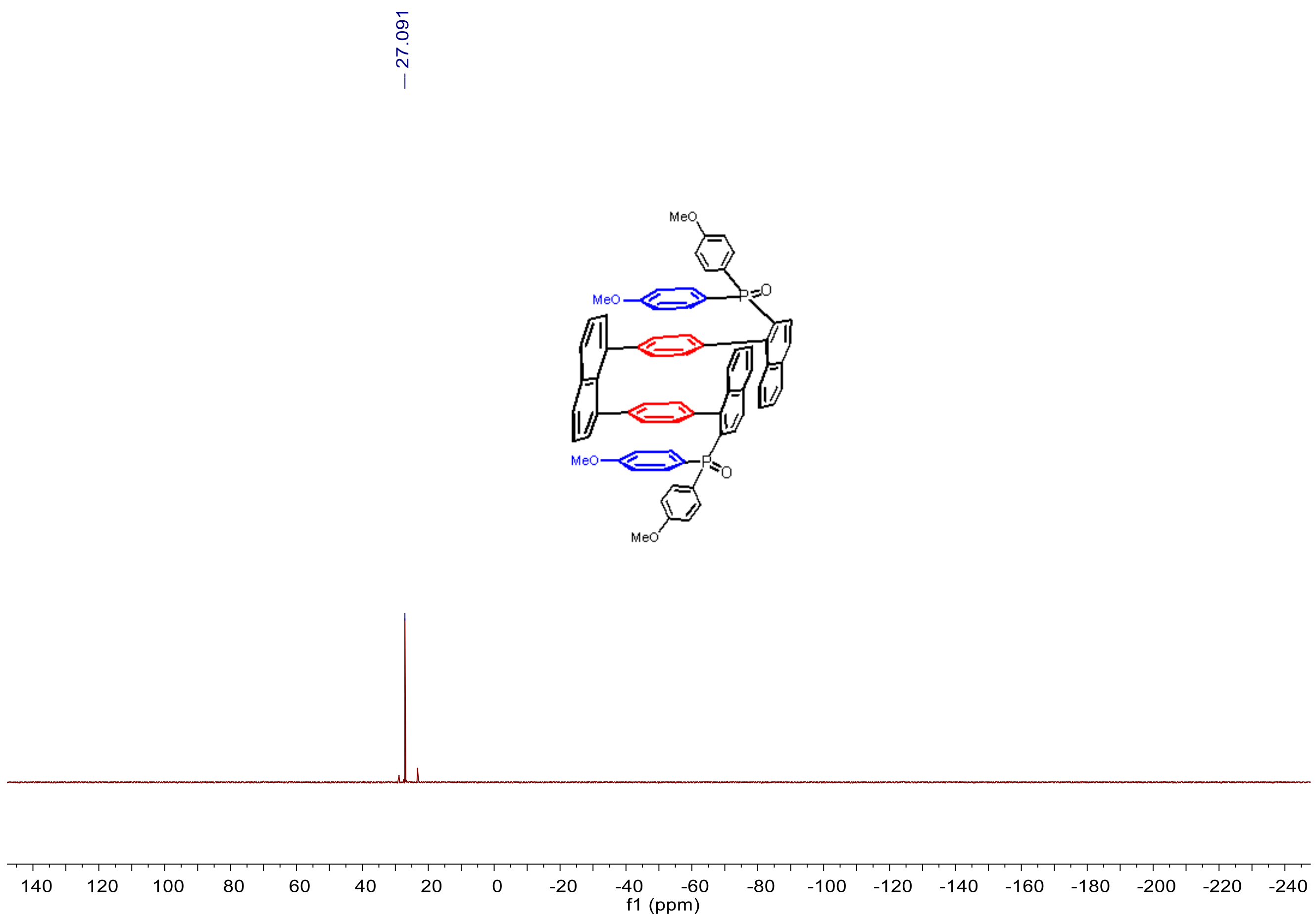

^{31}P NMR Spectrum of Compound 8c

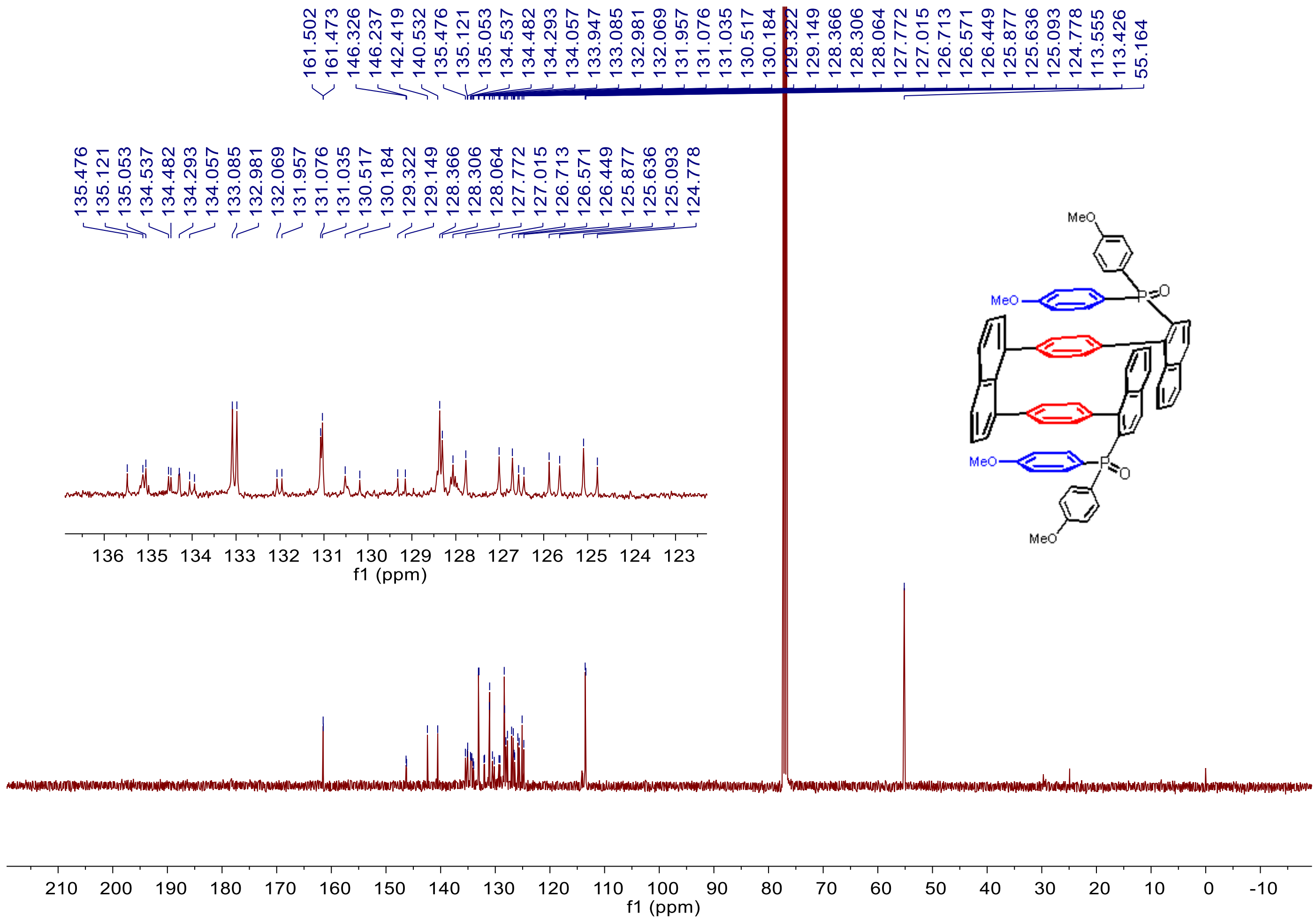

^{13}C NMR Spectrum of Compound 8c

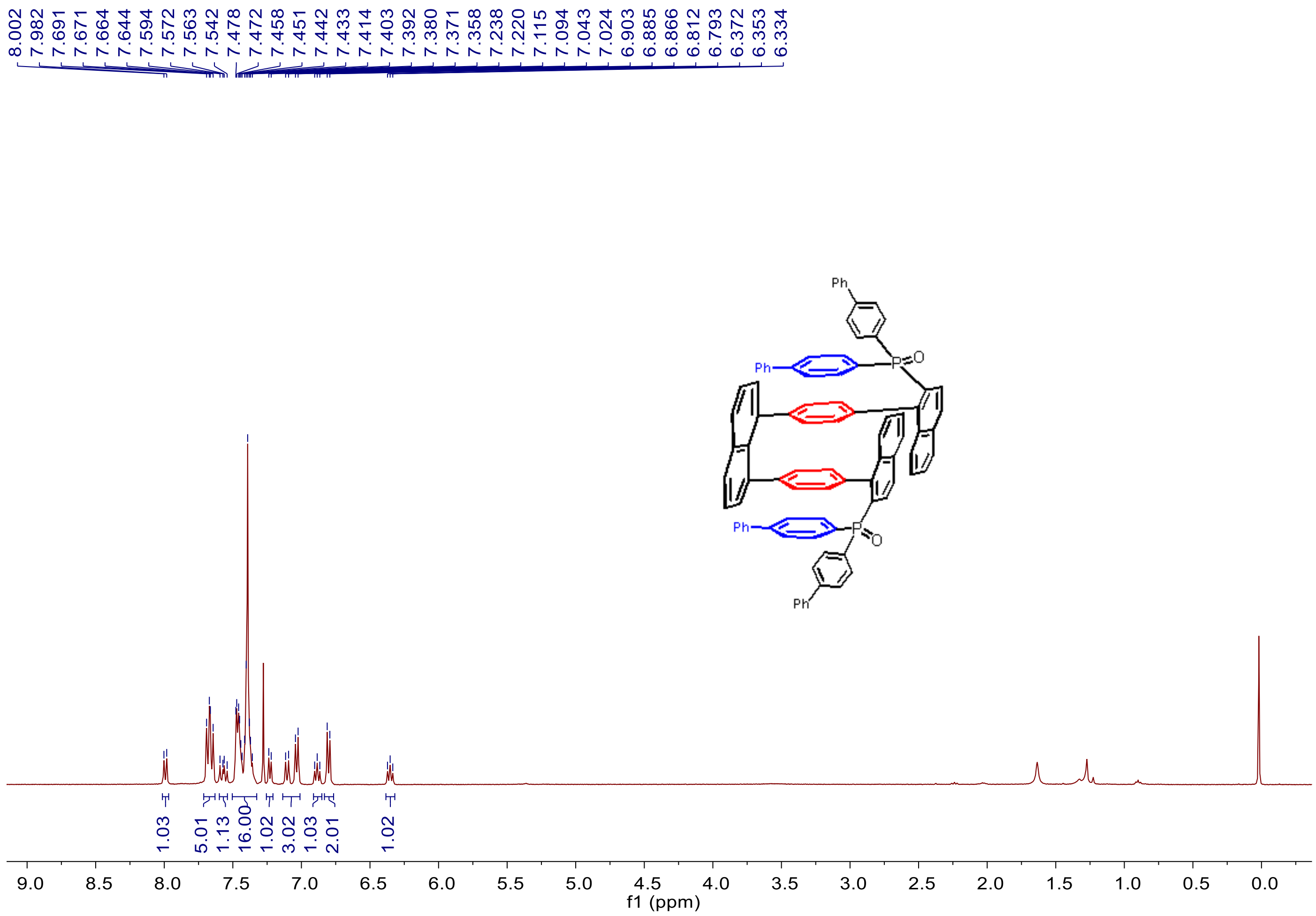

^{1}H NMR Spectrum of Compound 8d

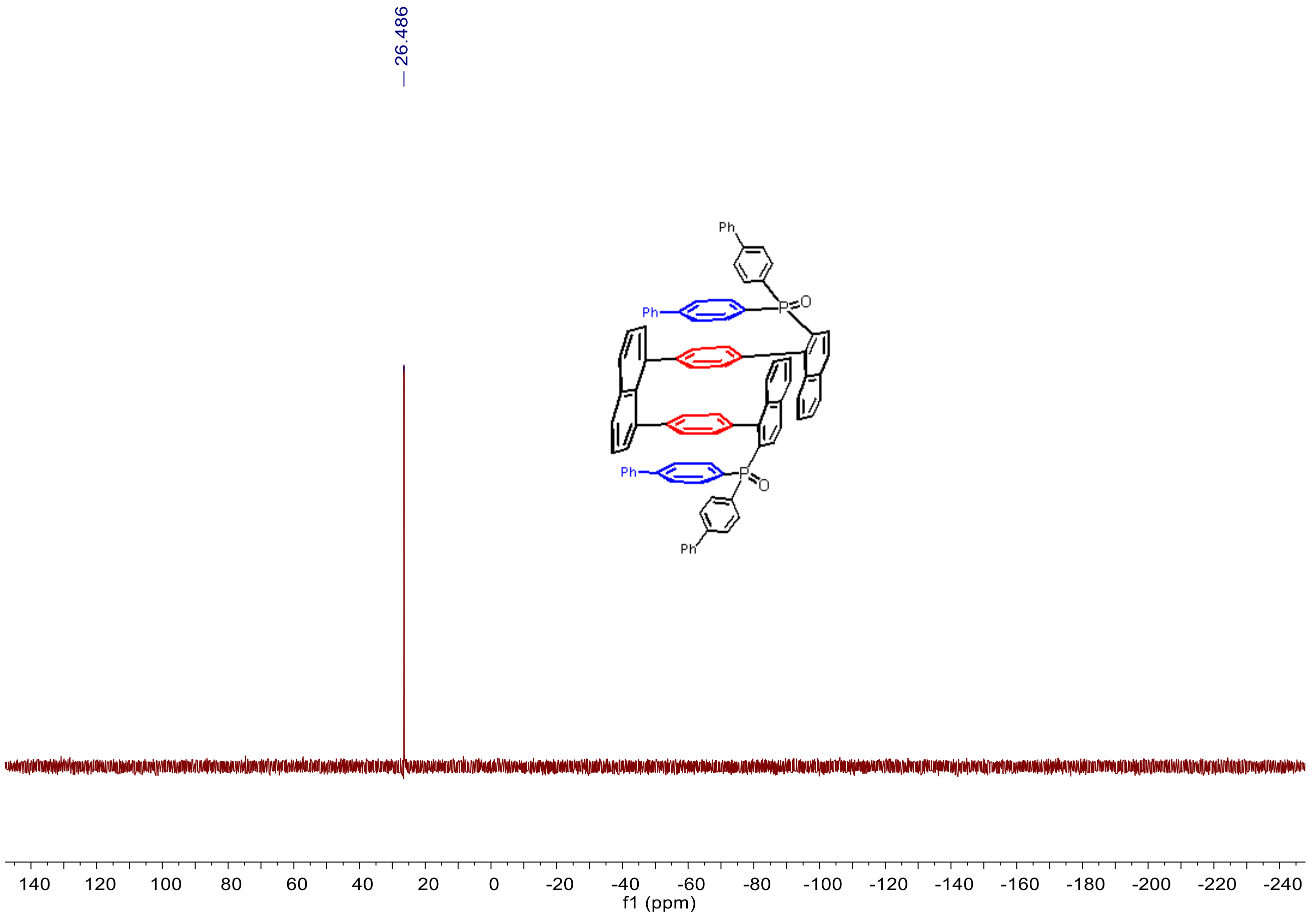

^{31}P NMR Spectrum of Compound 8d

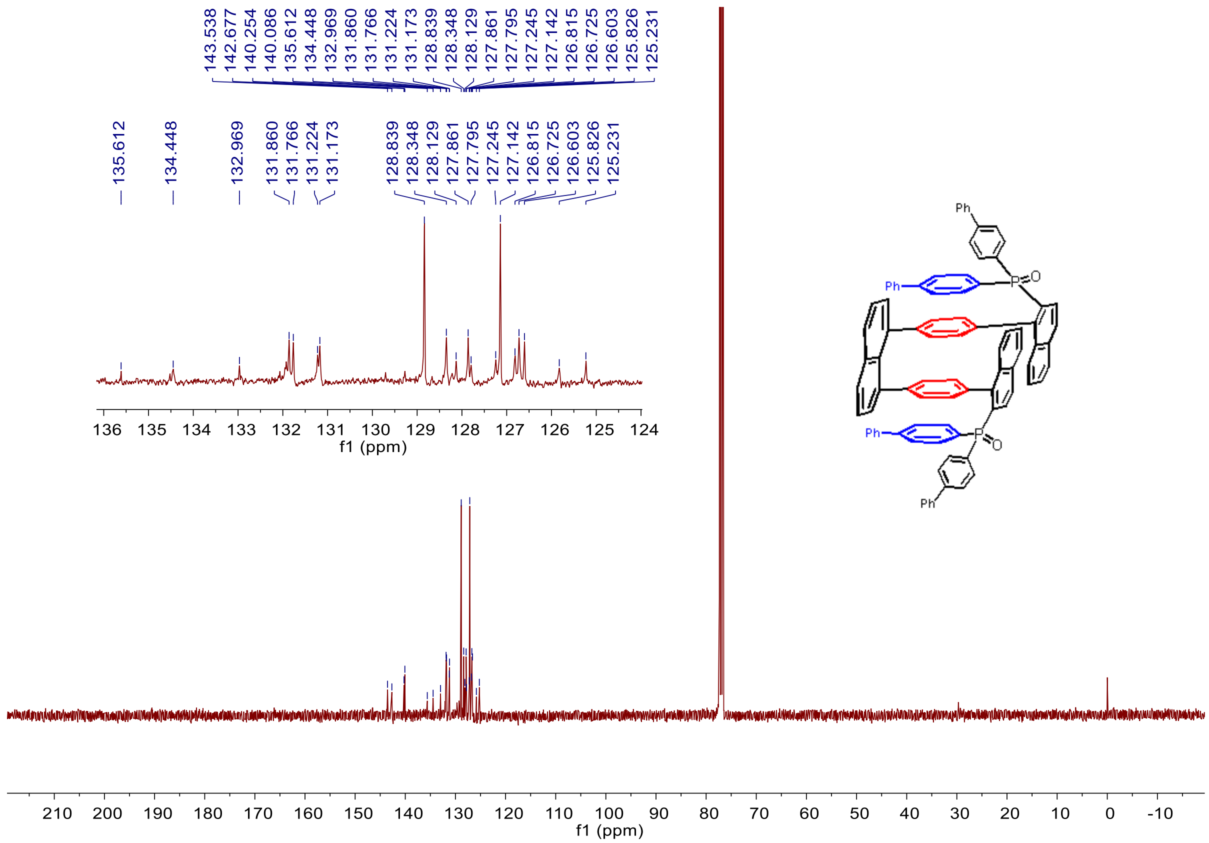

^{13}C NMR Spectrum of Compound 8d

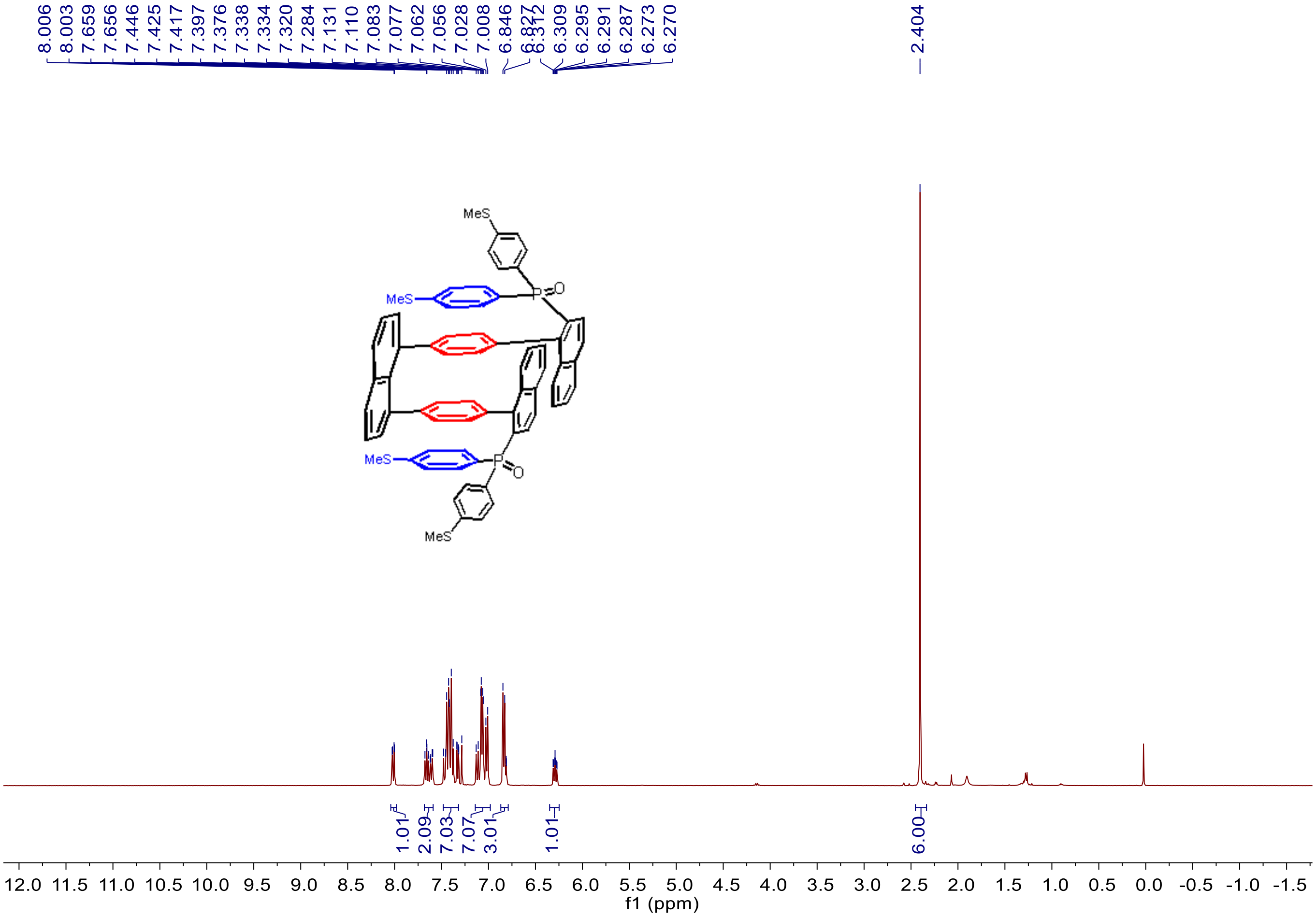

^{1}H NMR Spectrum of Compound 8e

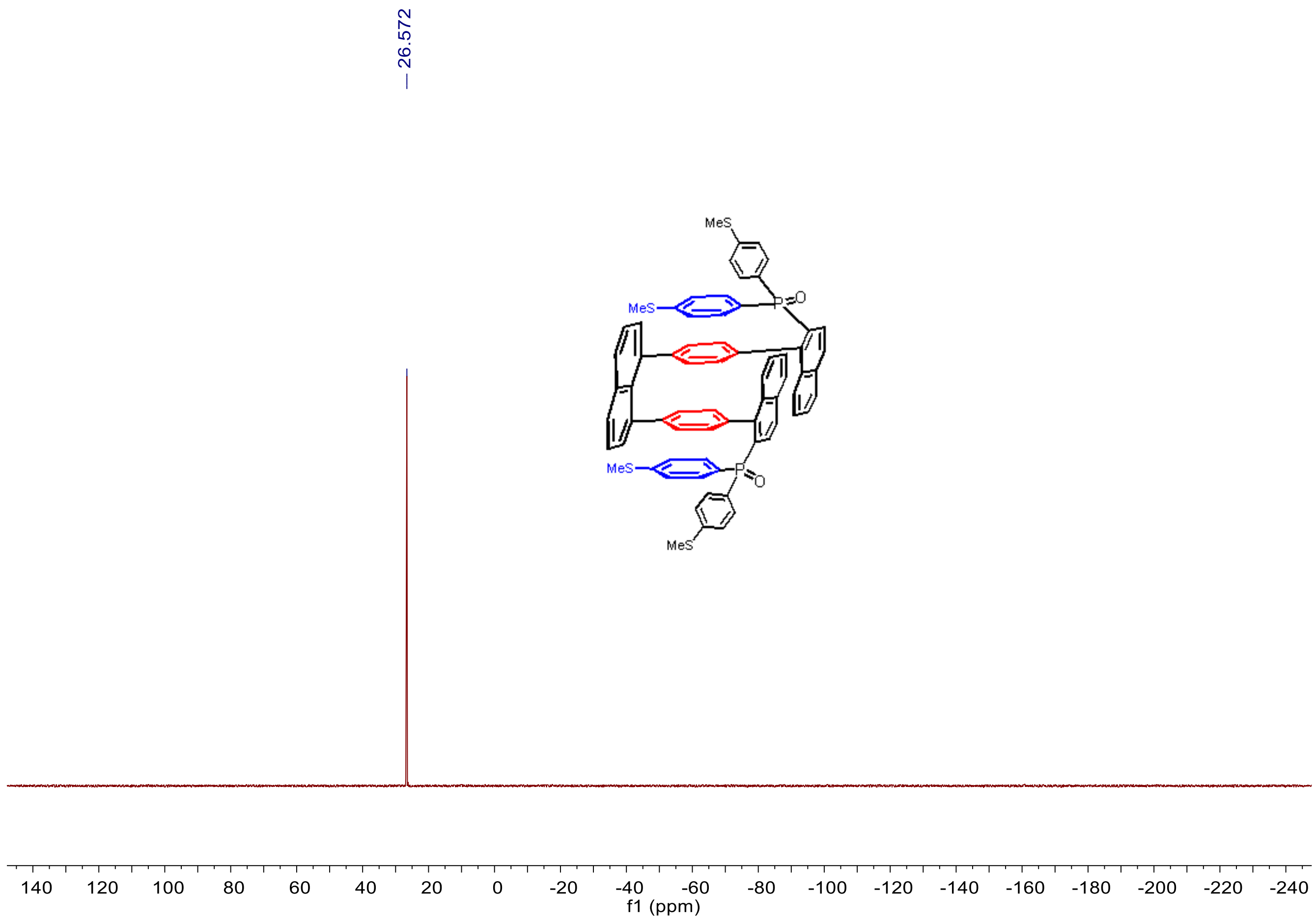

^{31}P NMR Spectrum of Compound 8e

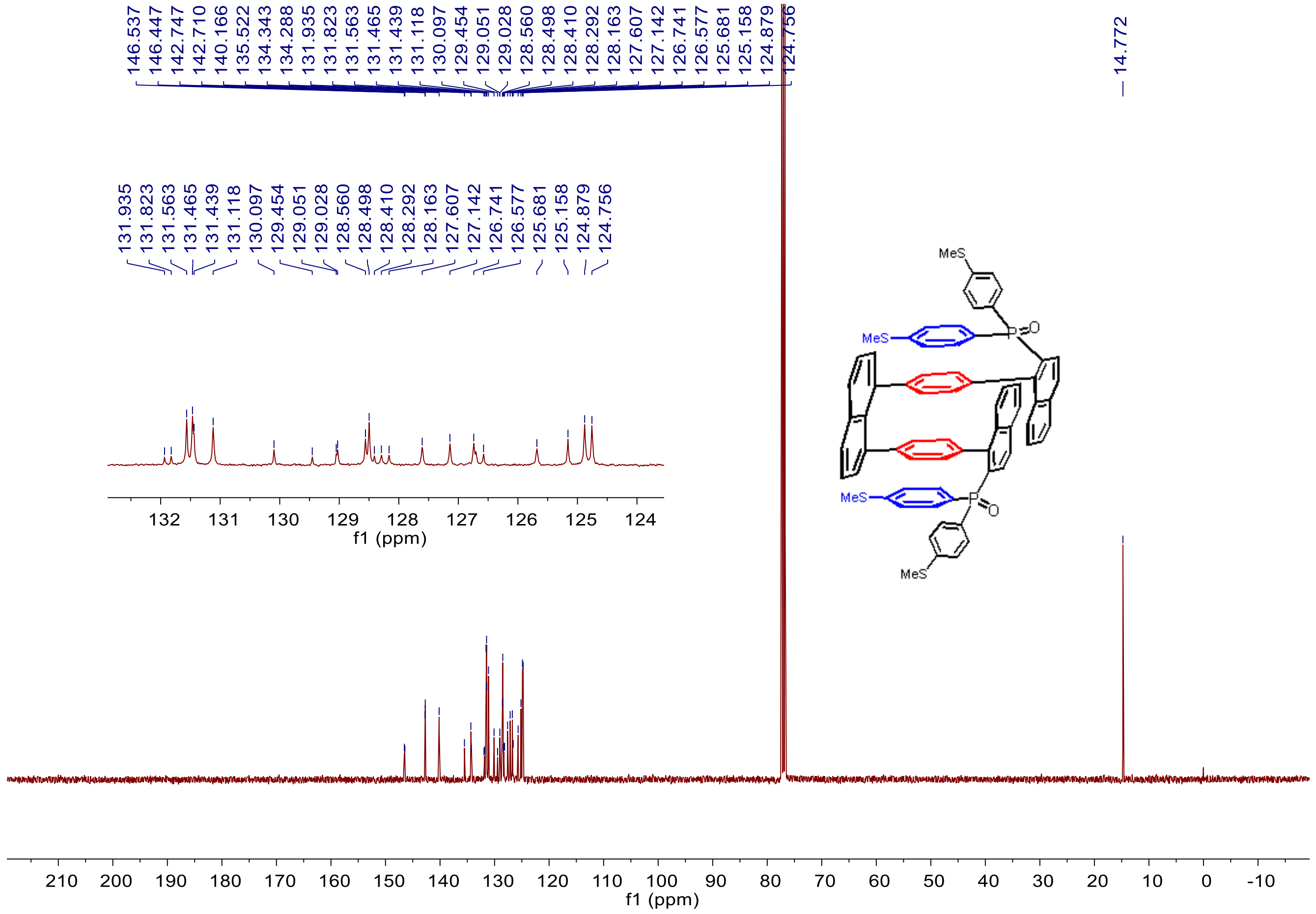

^{13}C NMR Spectrum of Compound 8e

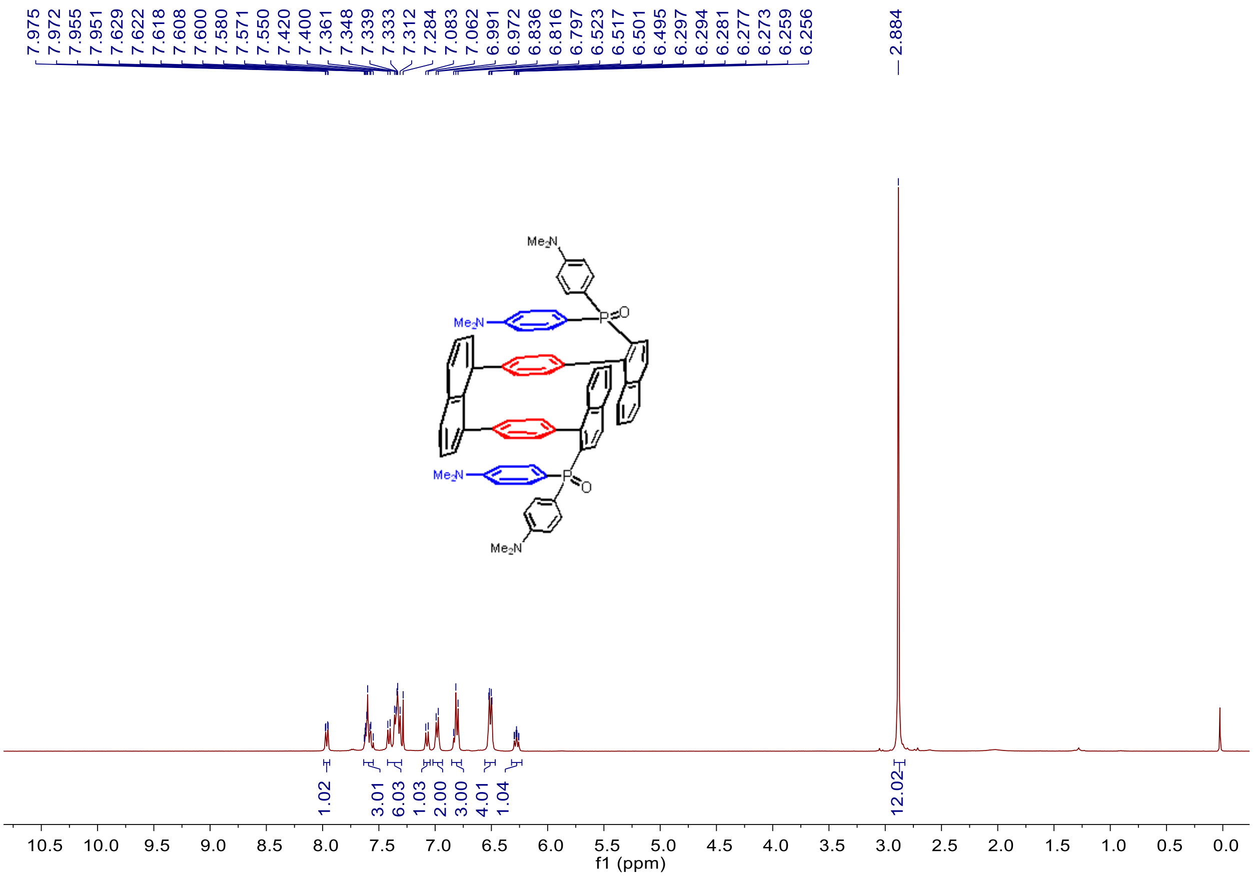

^{1}H NMR Spectrum of Compound 8f

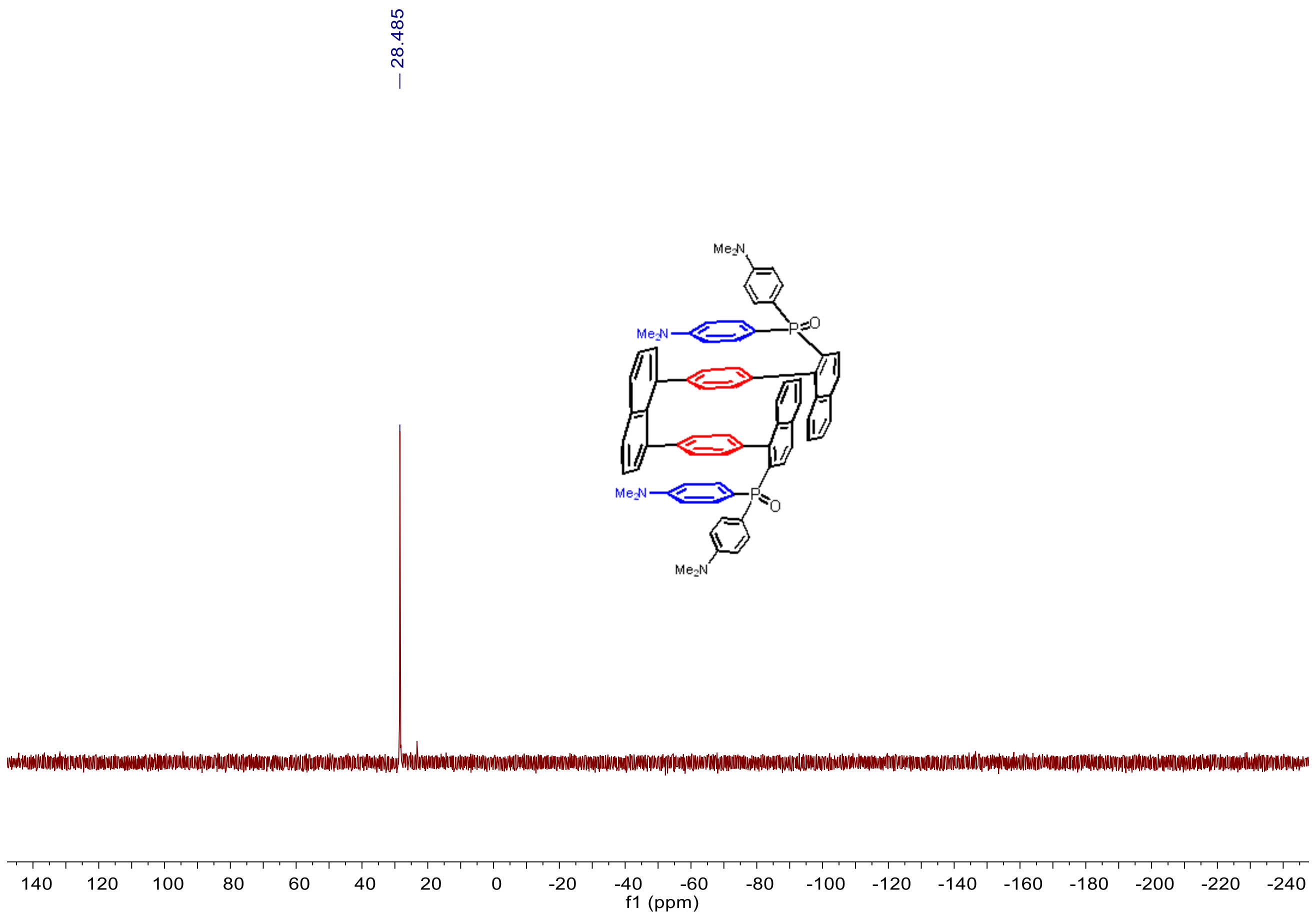

^{31}P NMR Spectrum of Compound 8f

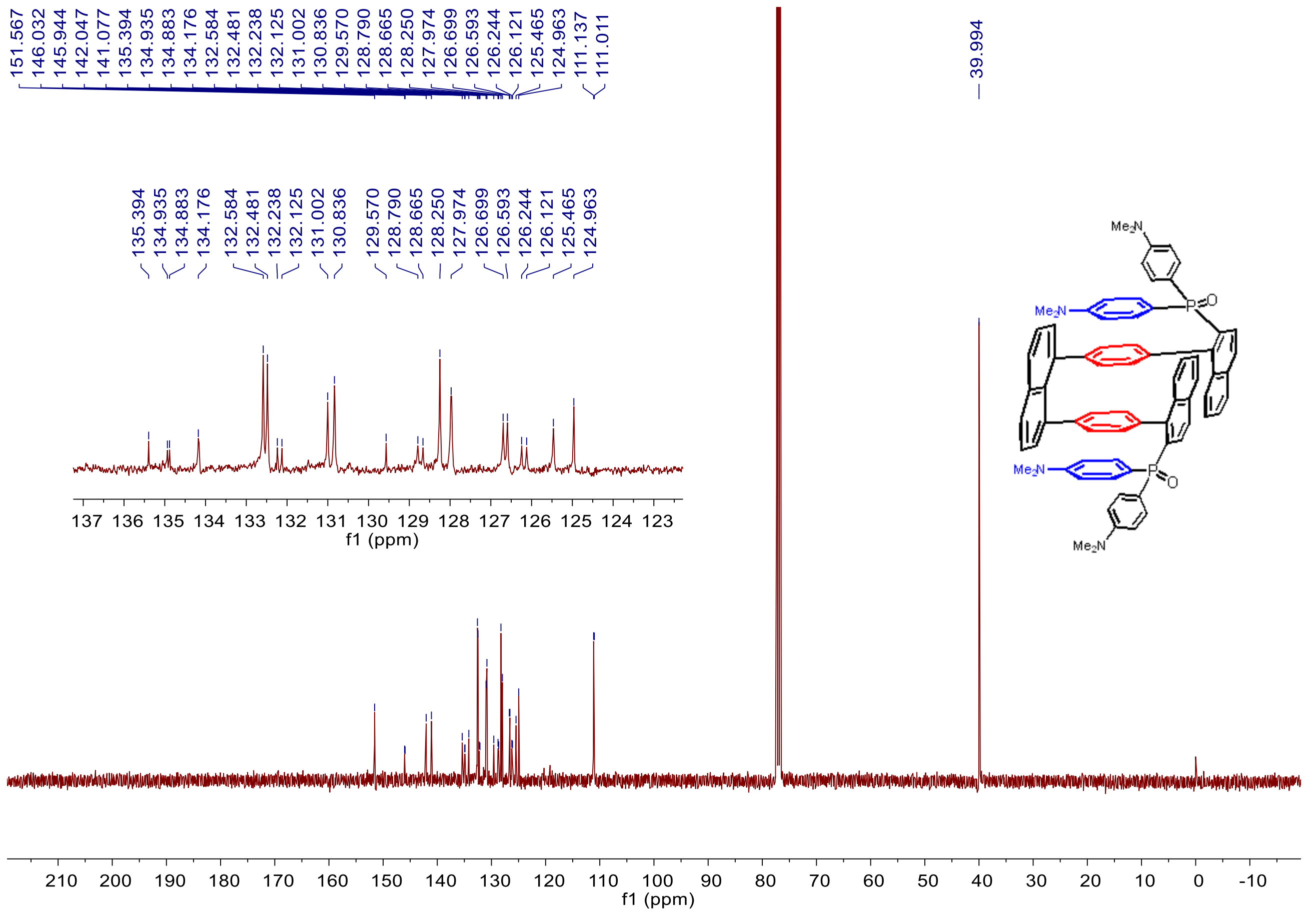

^{13}C NMR Spectrum of Compound 8f

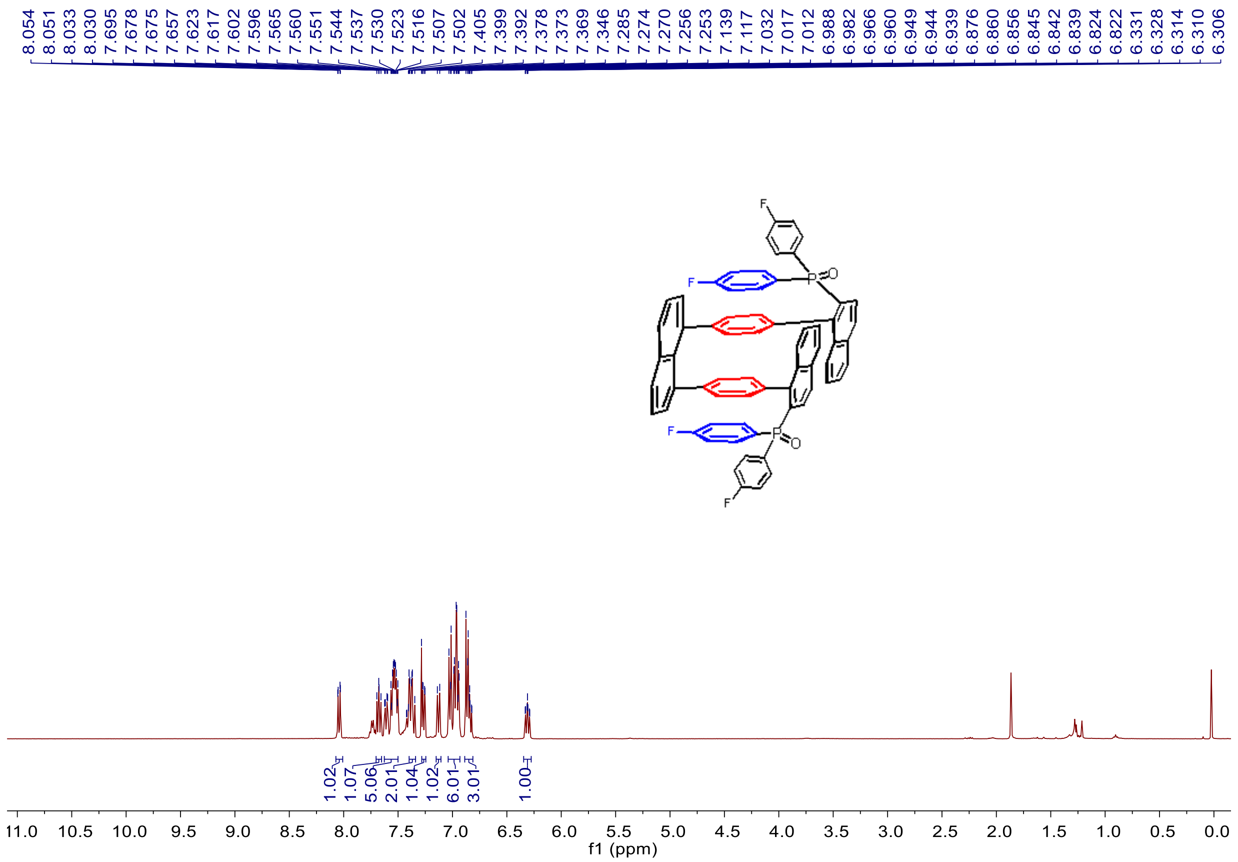

^{1}H NMR Spectrum of Compound 8g

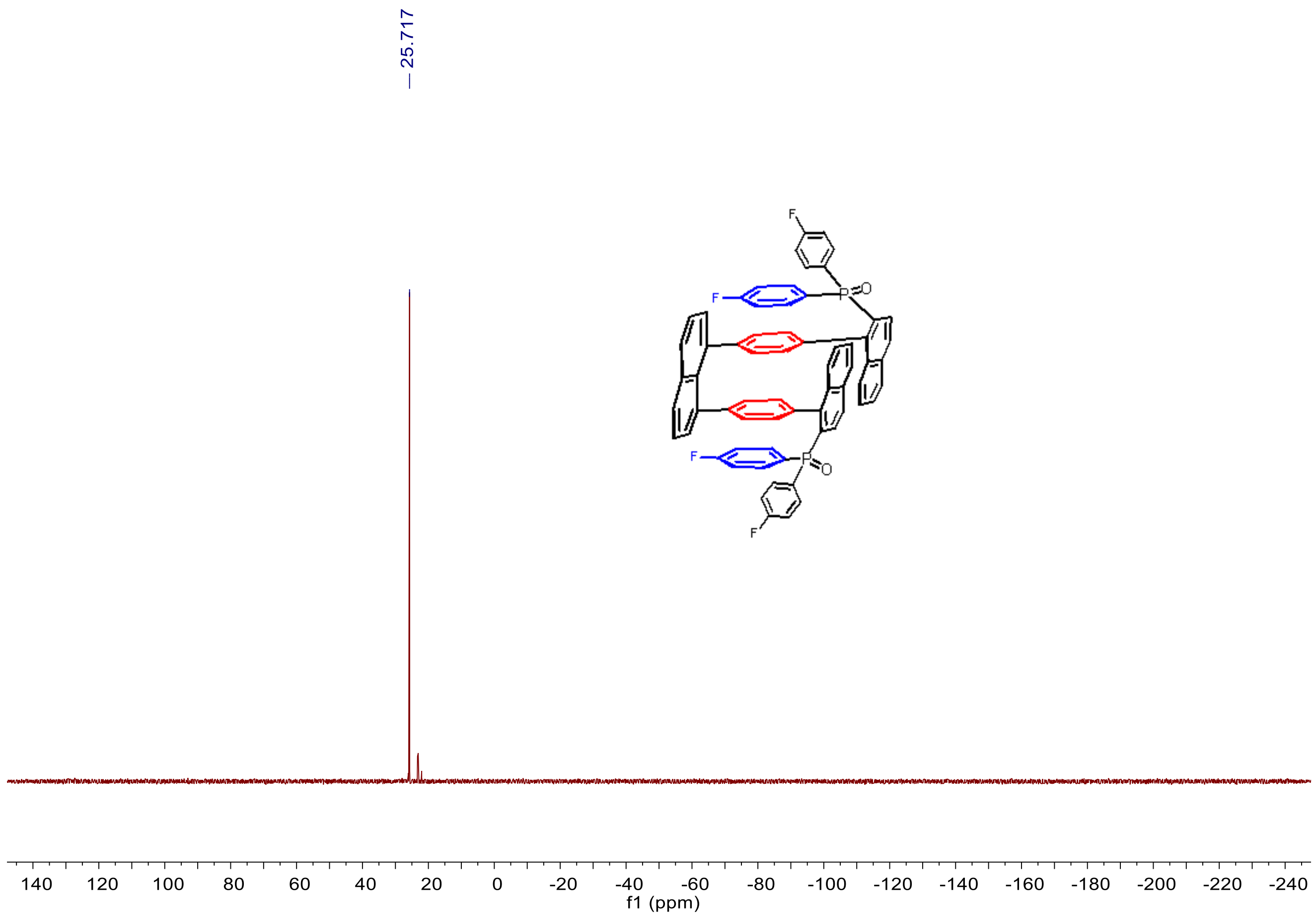

^{31}P NMR Spectrum of Compound 8g

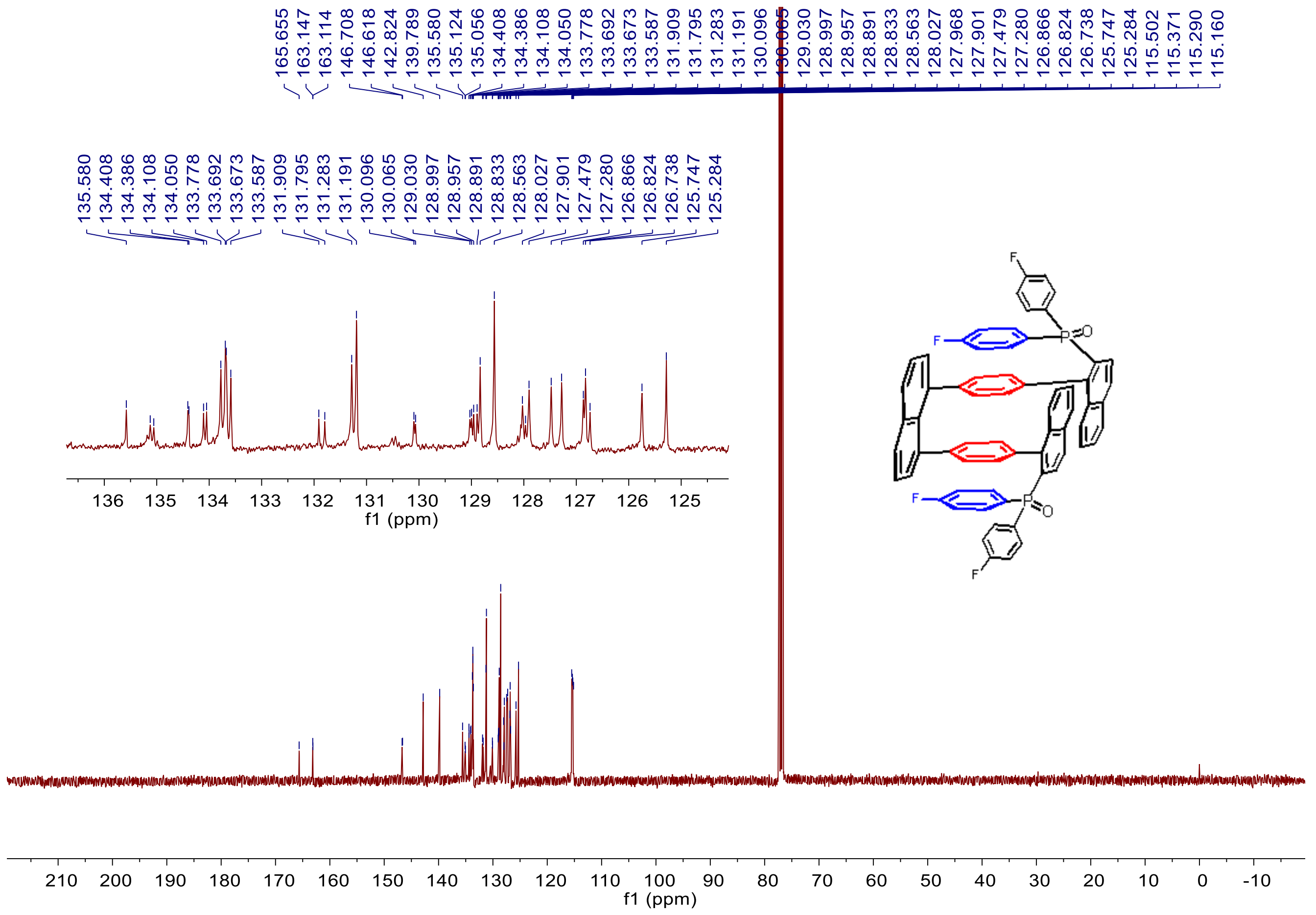

^{13}C NMR Spectrum of Compound 8g

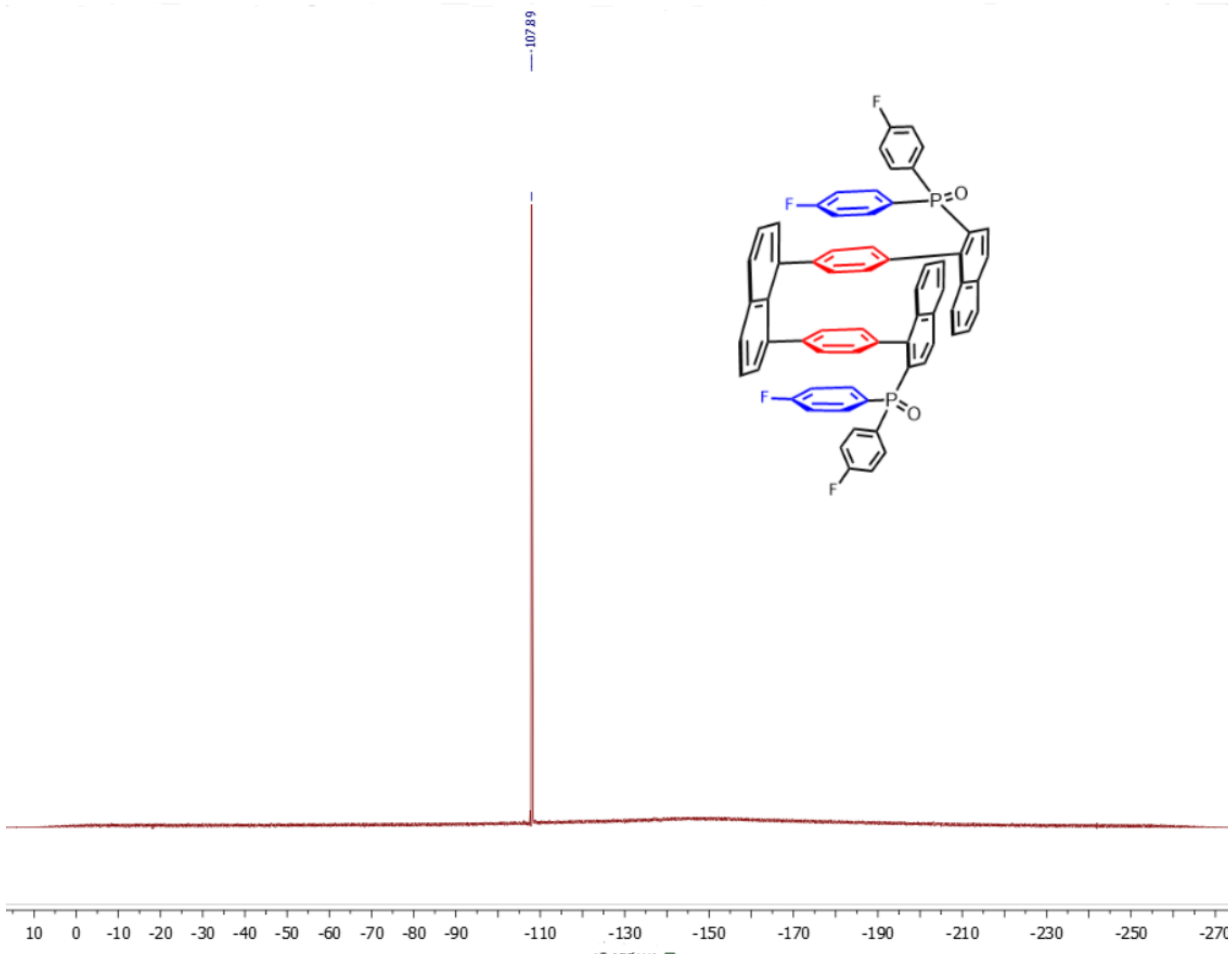

^{19}F NMR Spectrum of Compound 8g

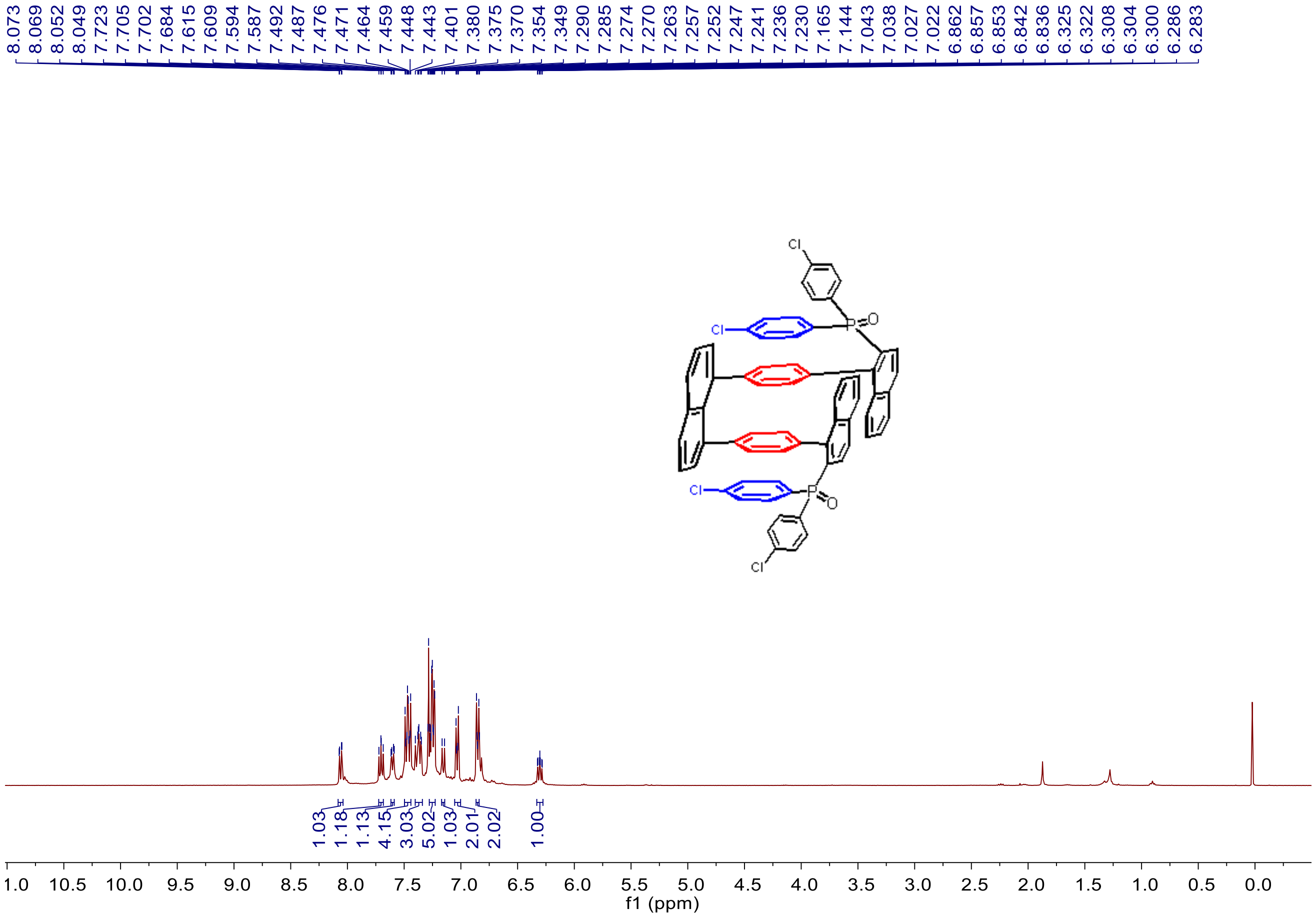

^{1}H NMR Spectrum of Compound 8h

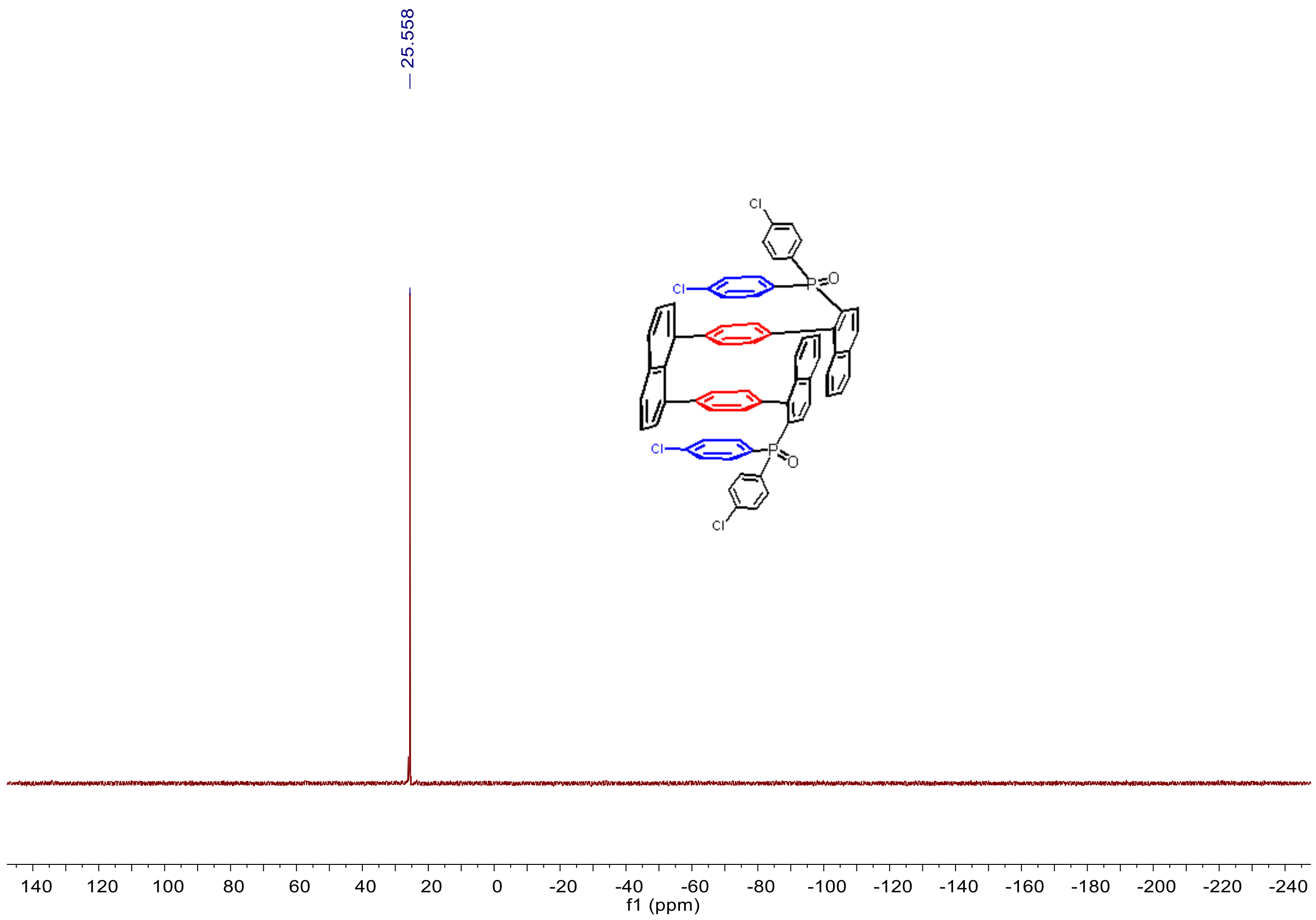

^{31}P NMR Spectrum of Compound 8h

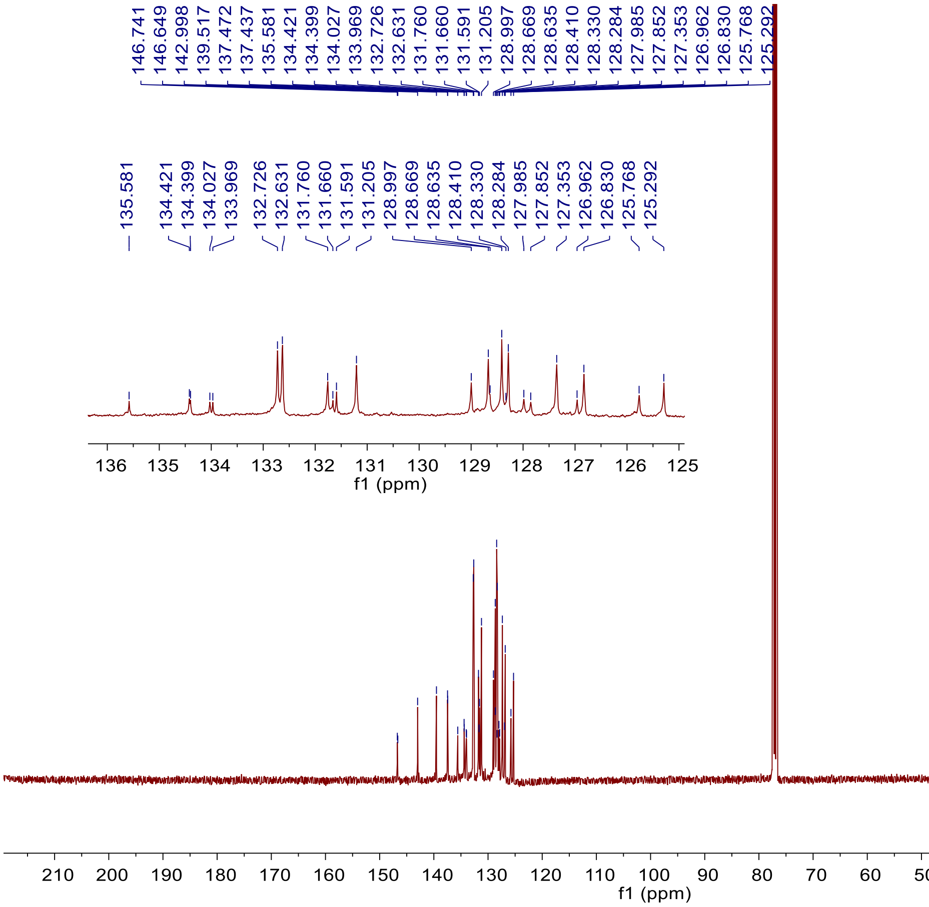

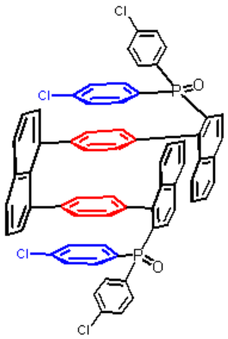

^{13}C NMR Spectrum of Compound 8h

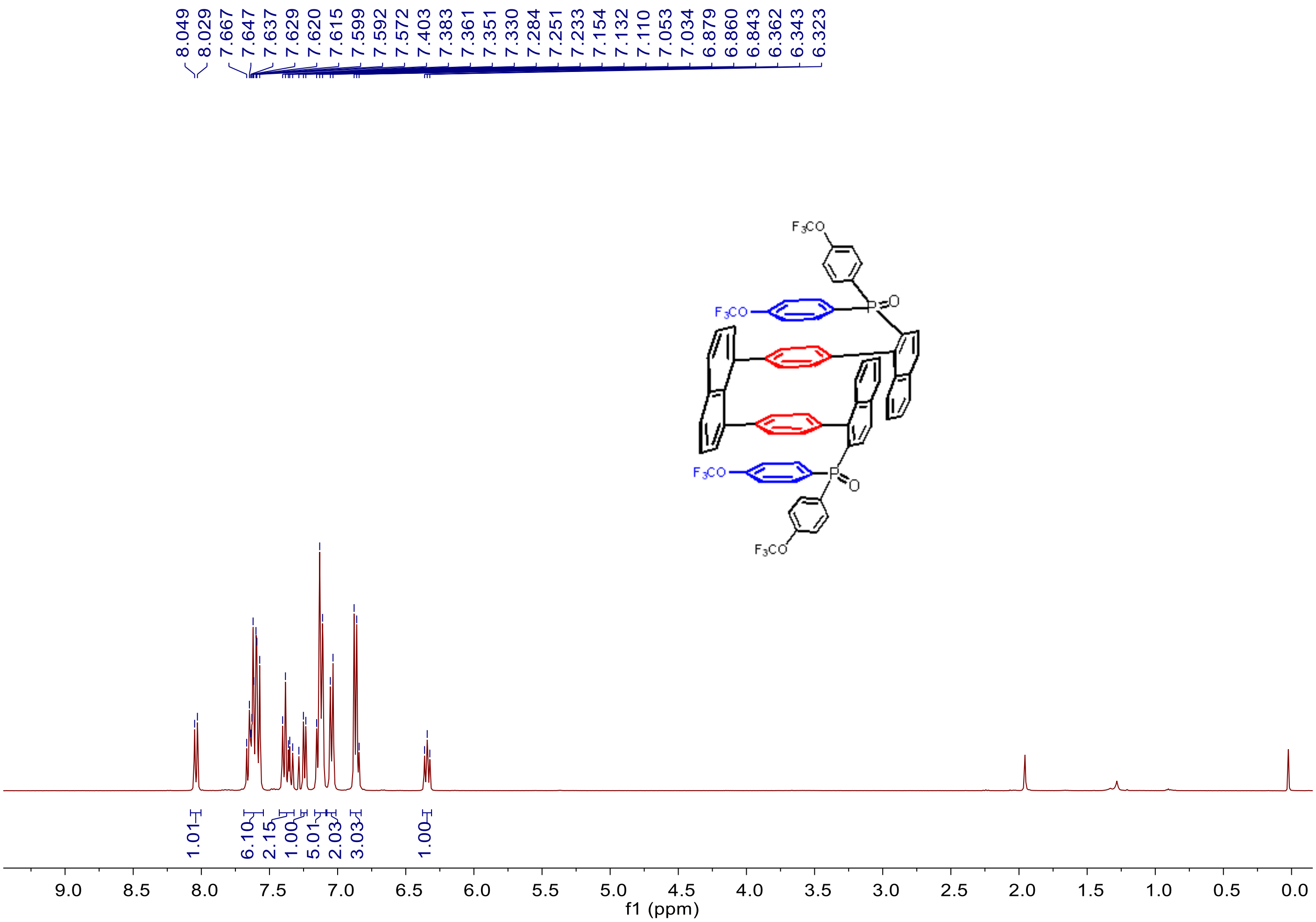

^{1}H NMR Spectrum of Compound 8i

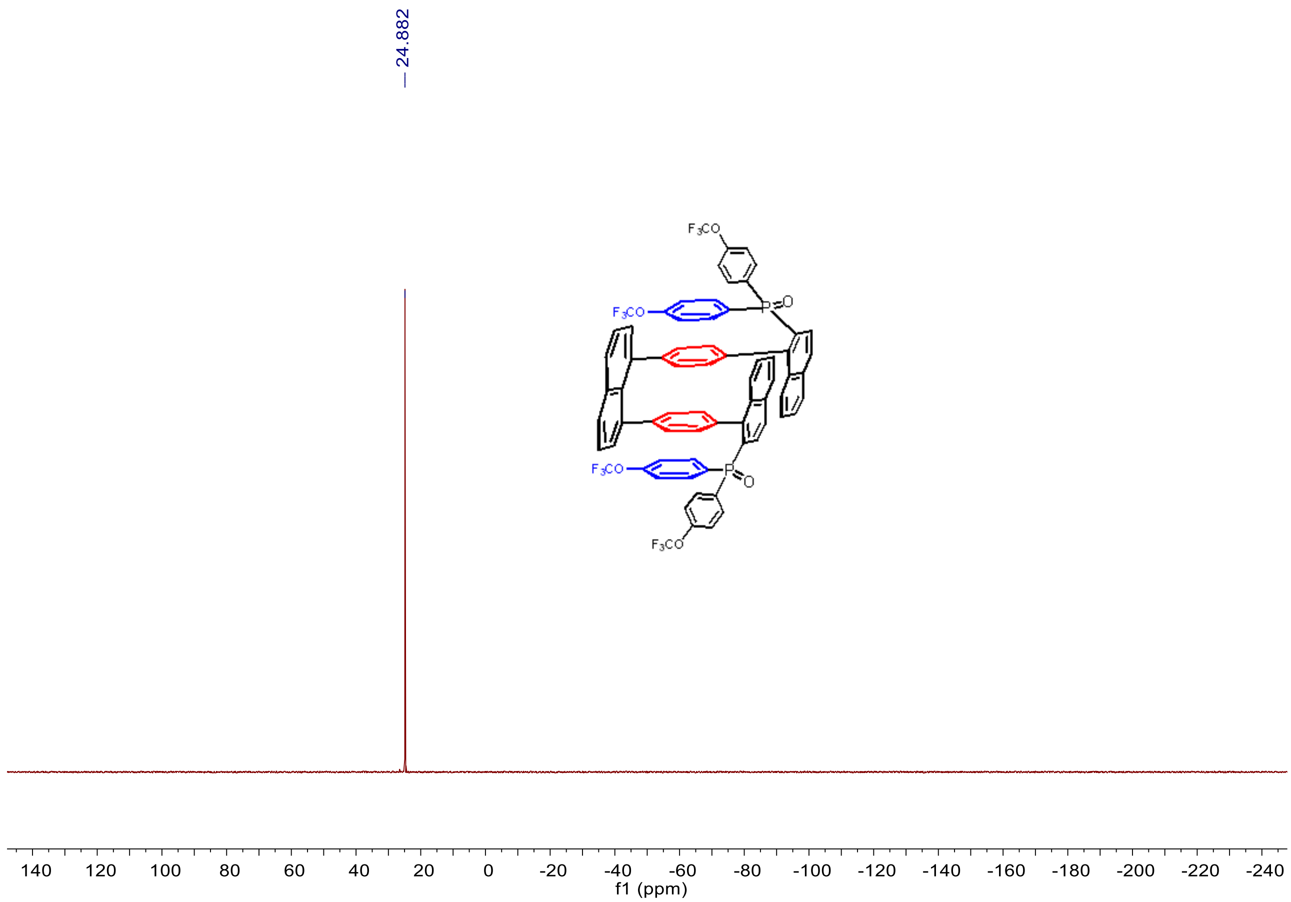

^{31}P NMR Spectrum of Compound 8i

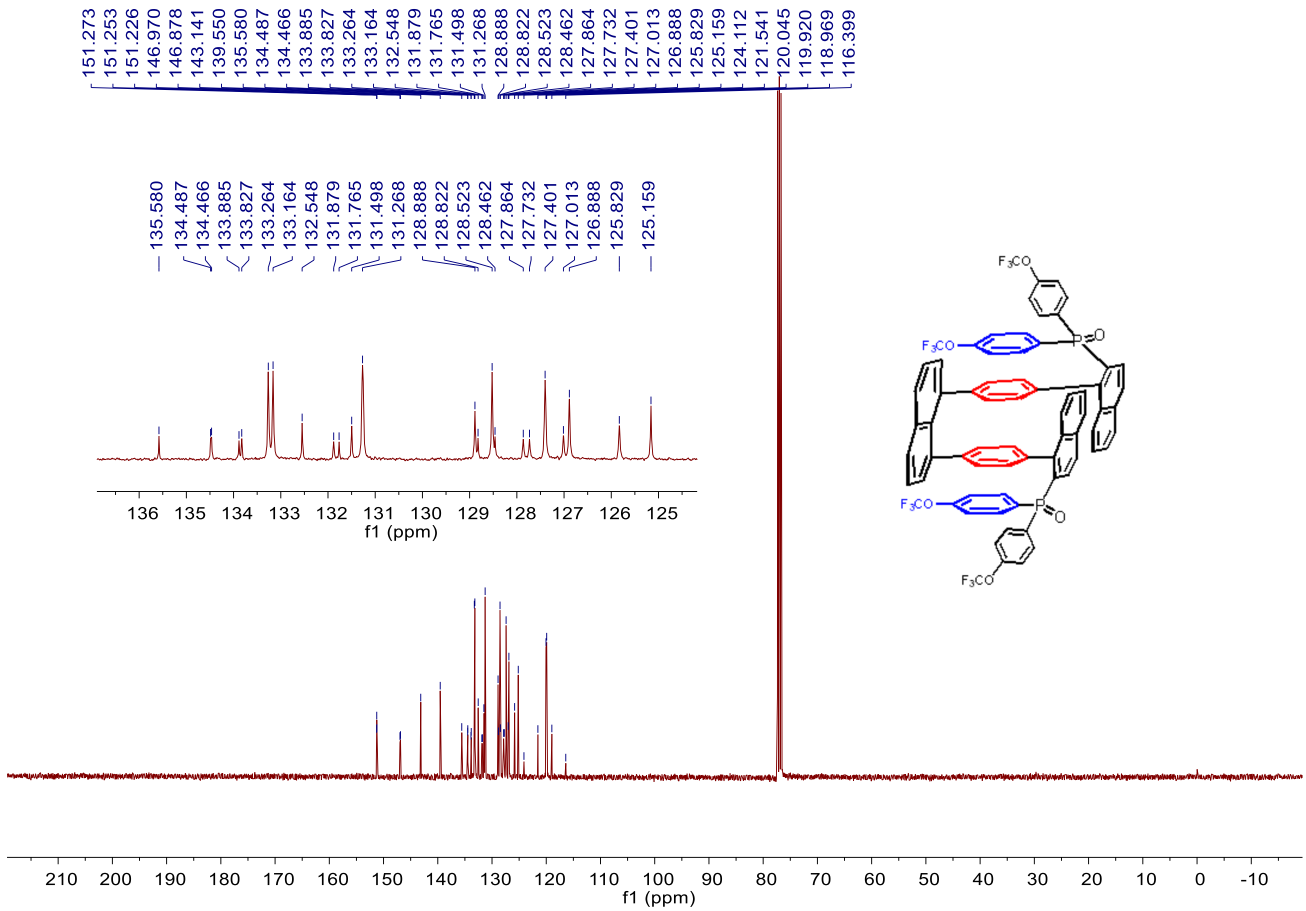

^{13}C NMR Spectrum of Compound 8i

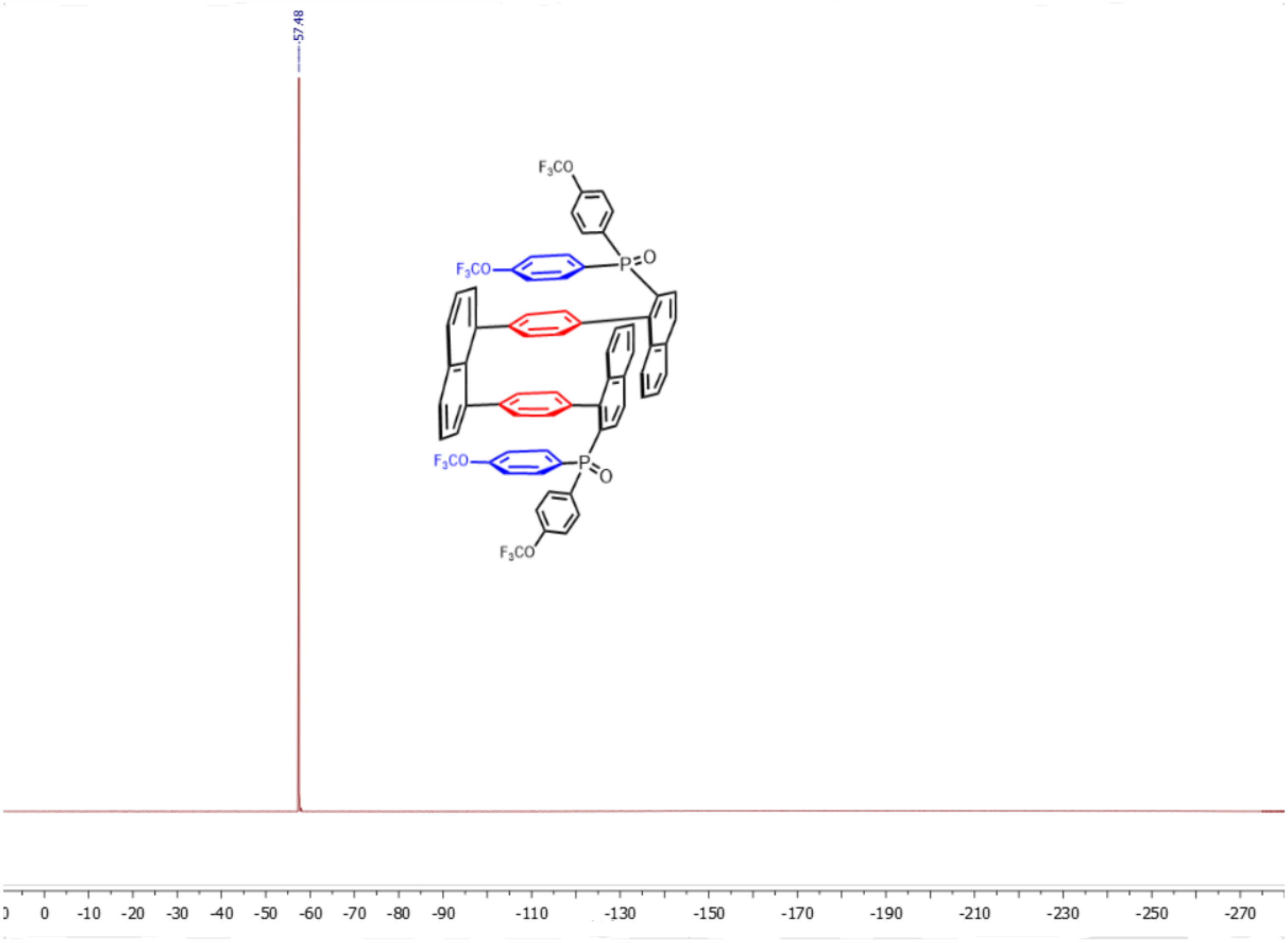

^{19}F NMR Spectrum of Compound 8i

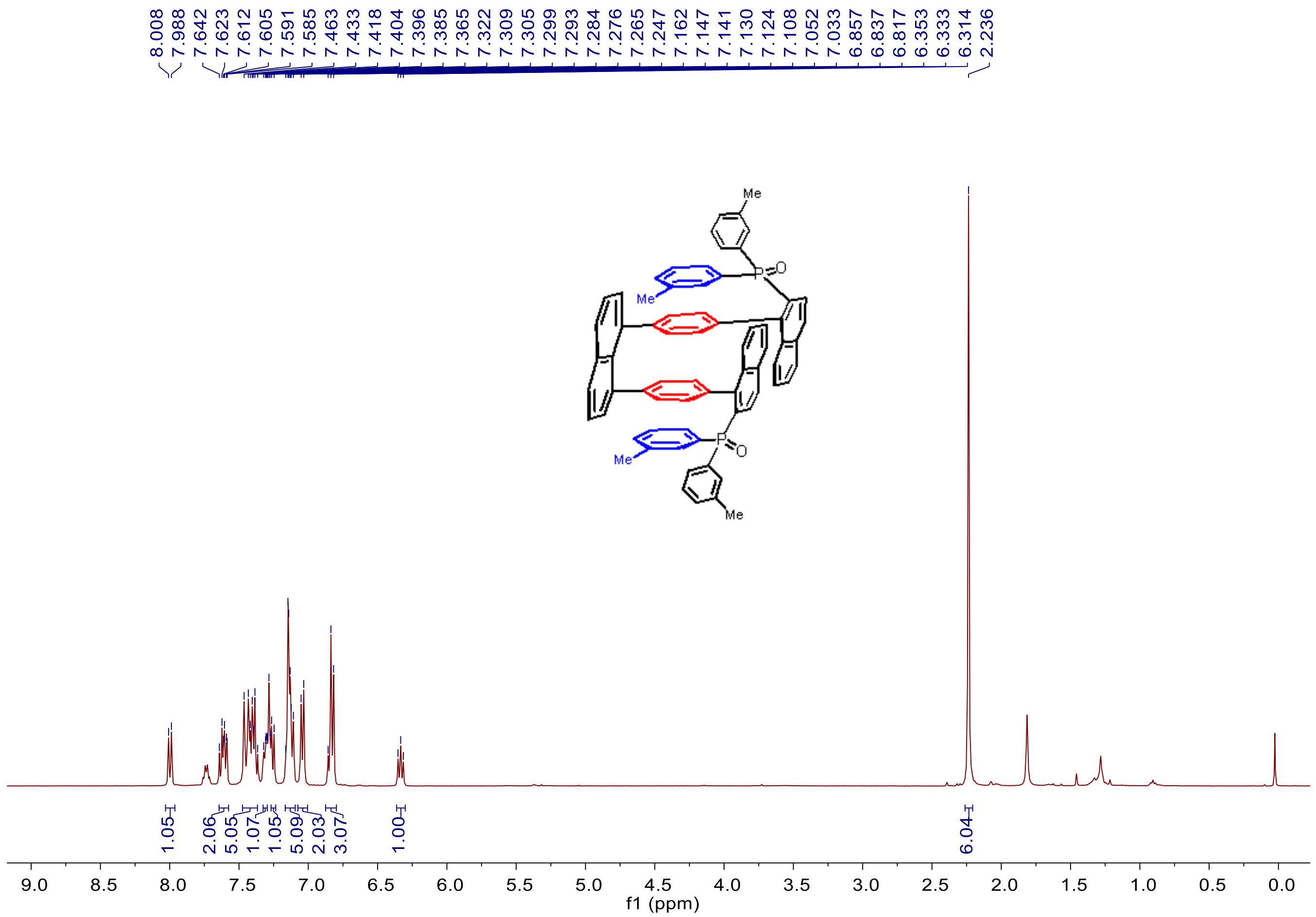

^{1}H NMR Spectrum of Compound 8j

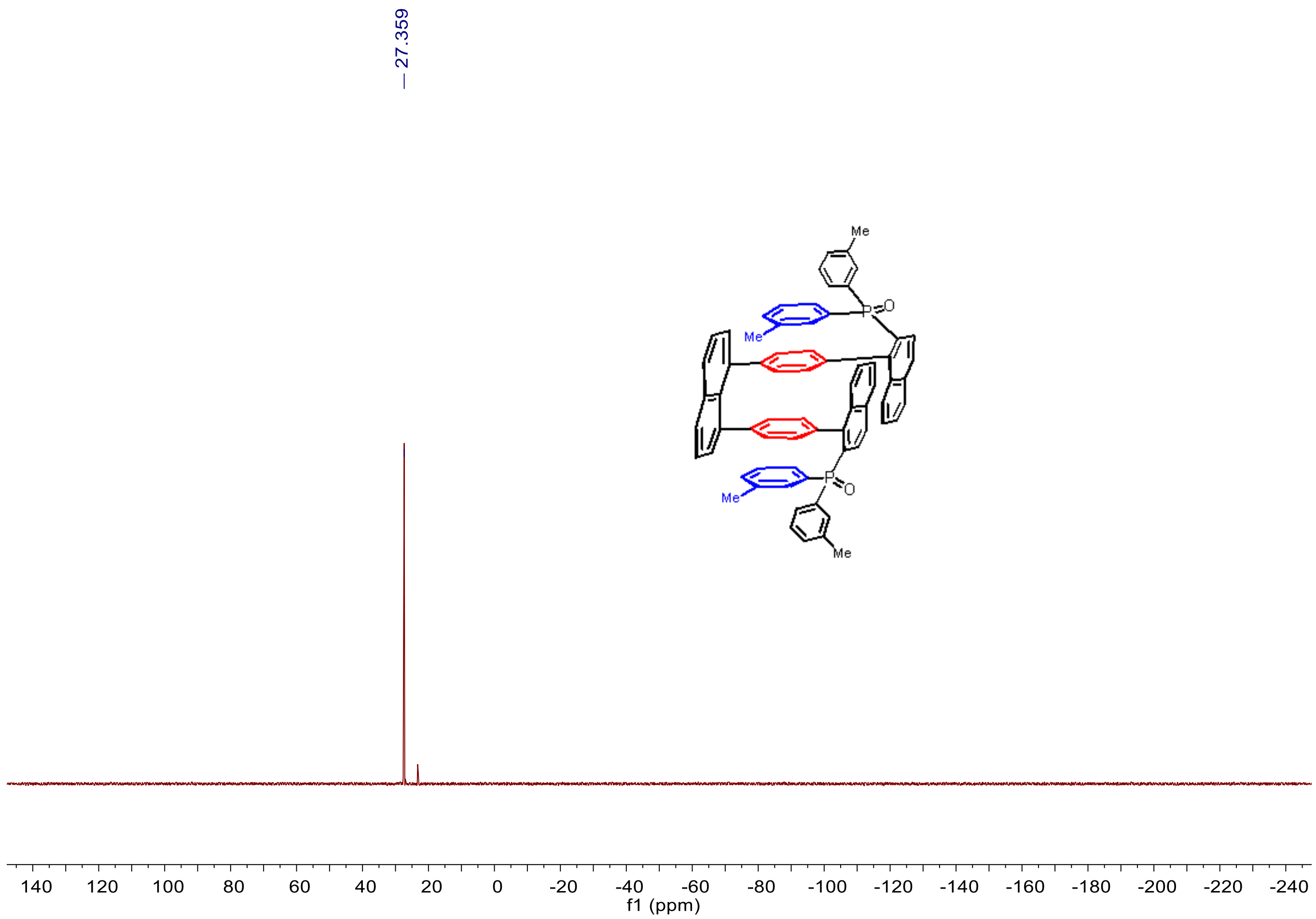

^{31}P NMR Spectrum of Compound 8j

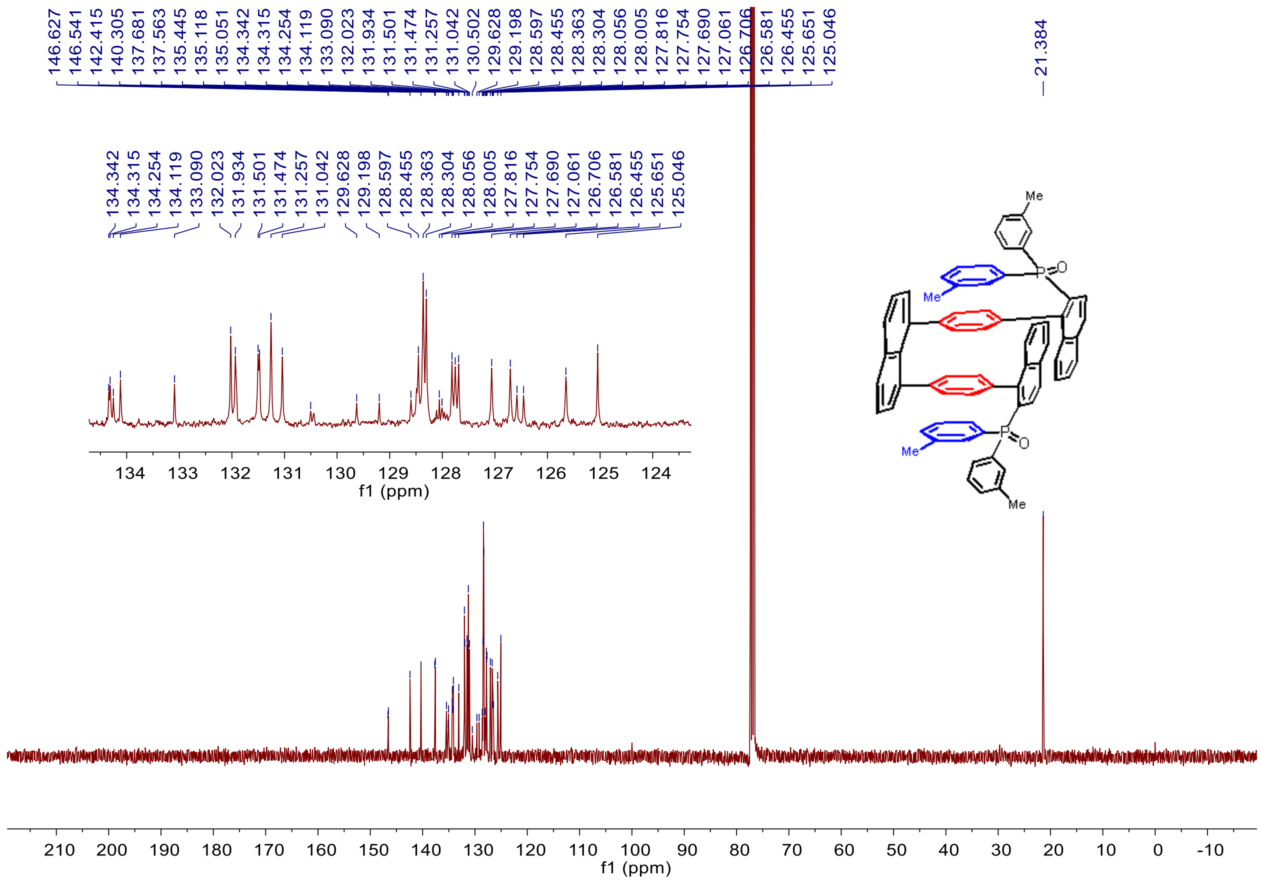

^{13}C NMR Spectrum of Compound 8j

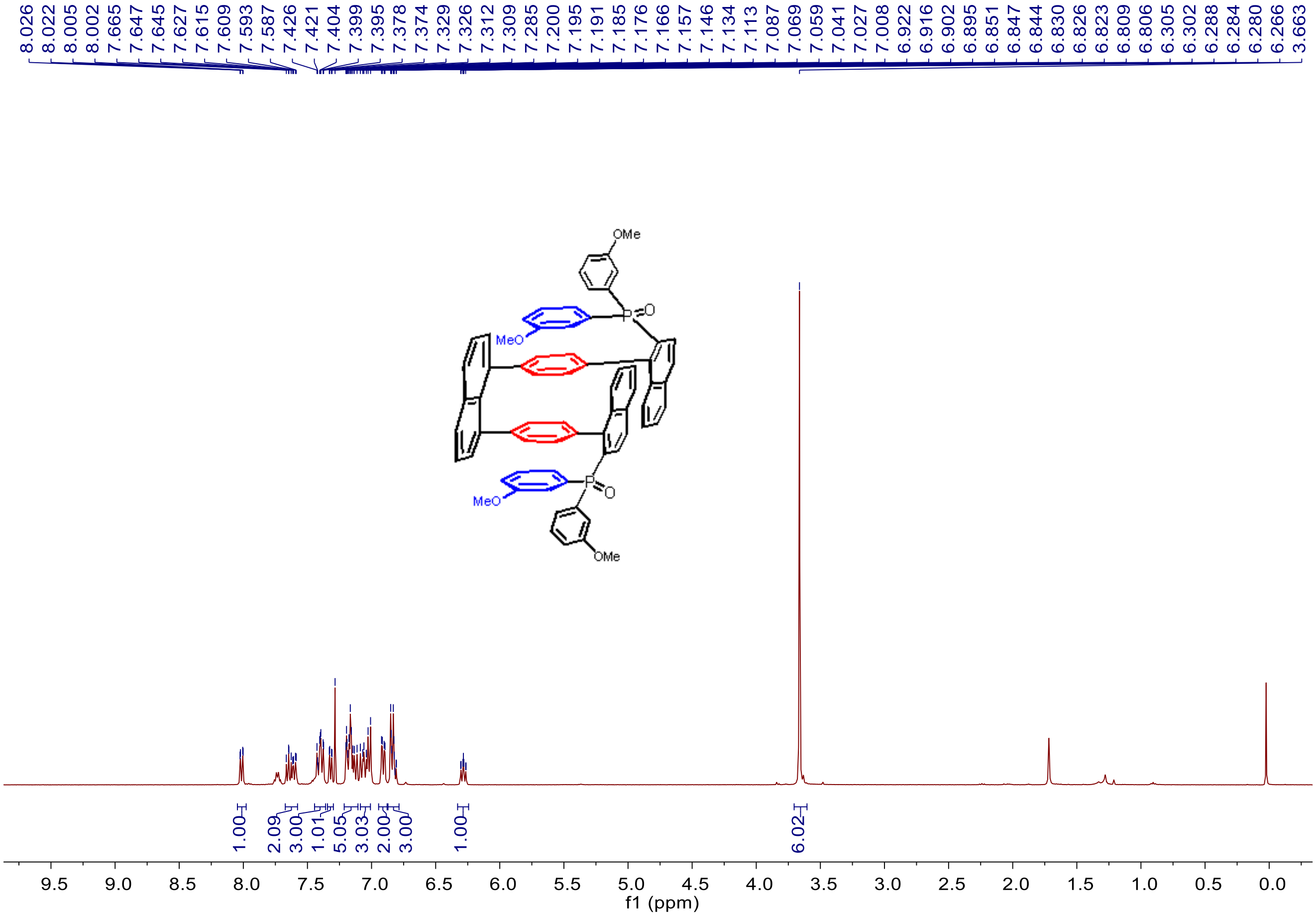

^{1}H NMR Spectrum of Compound 8k

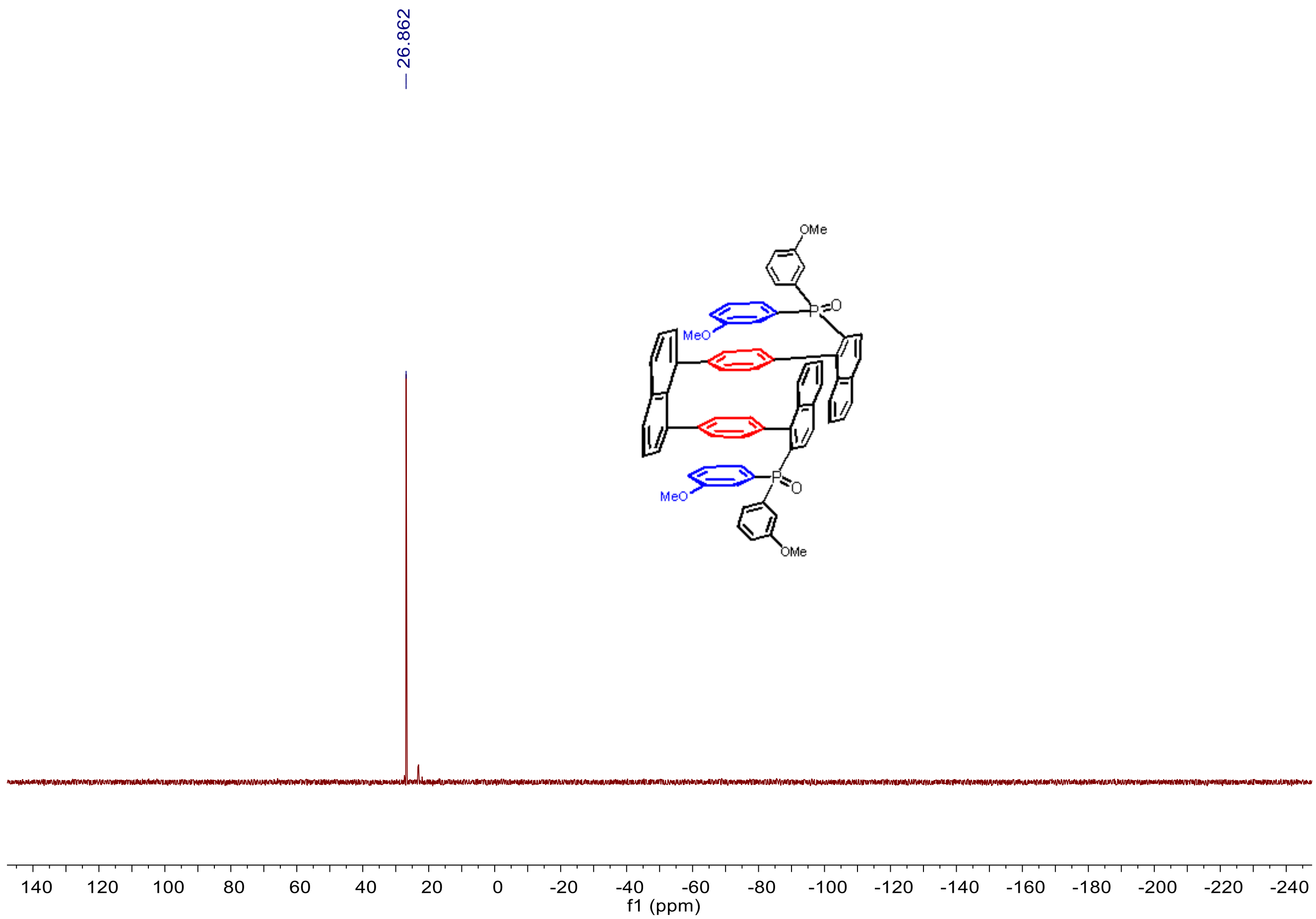

^{31}P NMR Spectrum of Compound 8k

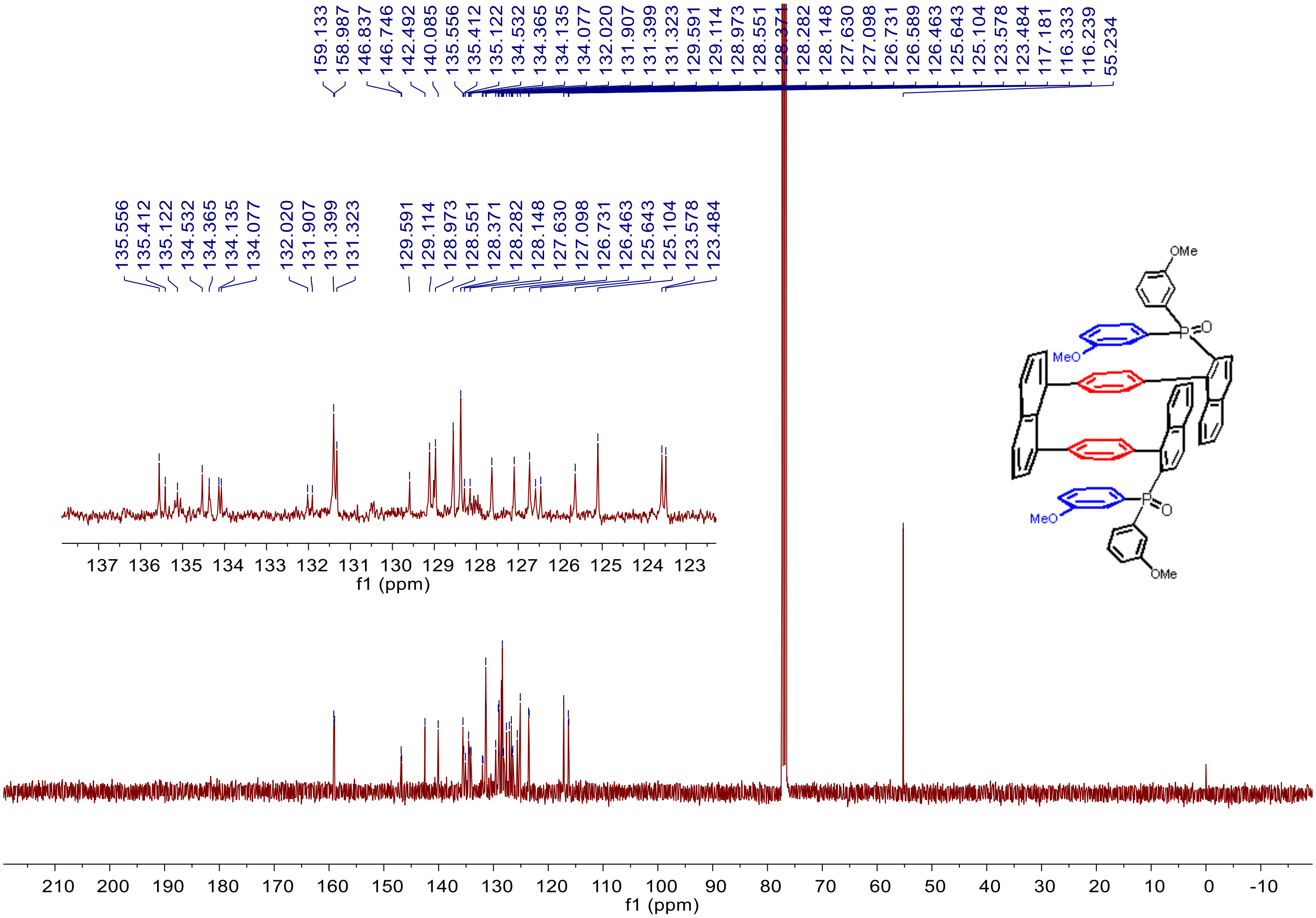

^{13}C NMR Spectrum of Compound 8k

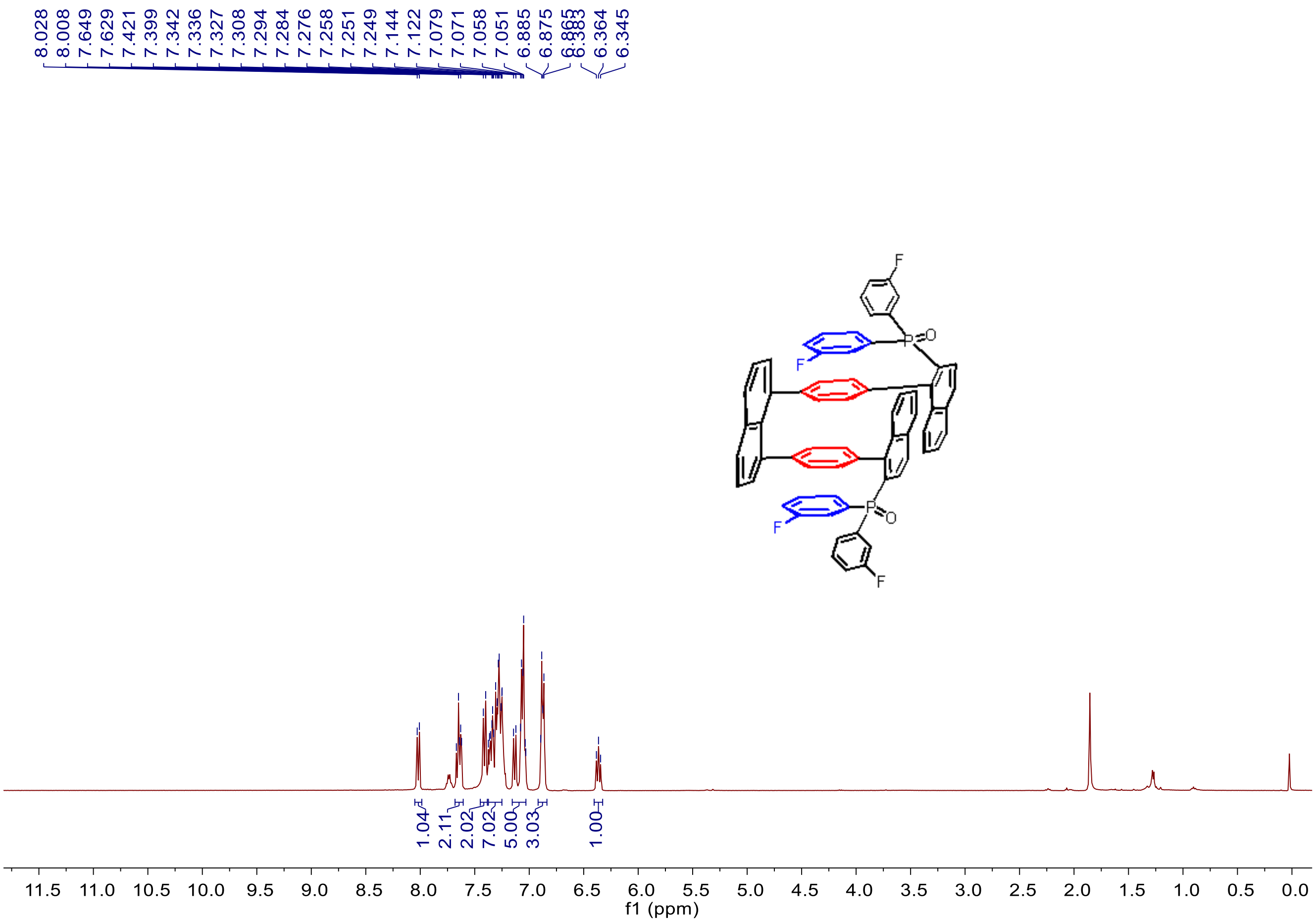

^{1}H NMR Spectrum of Compound 8l

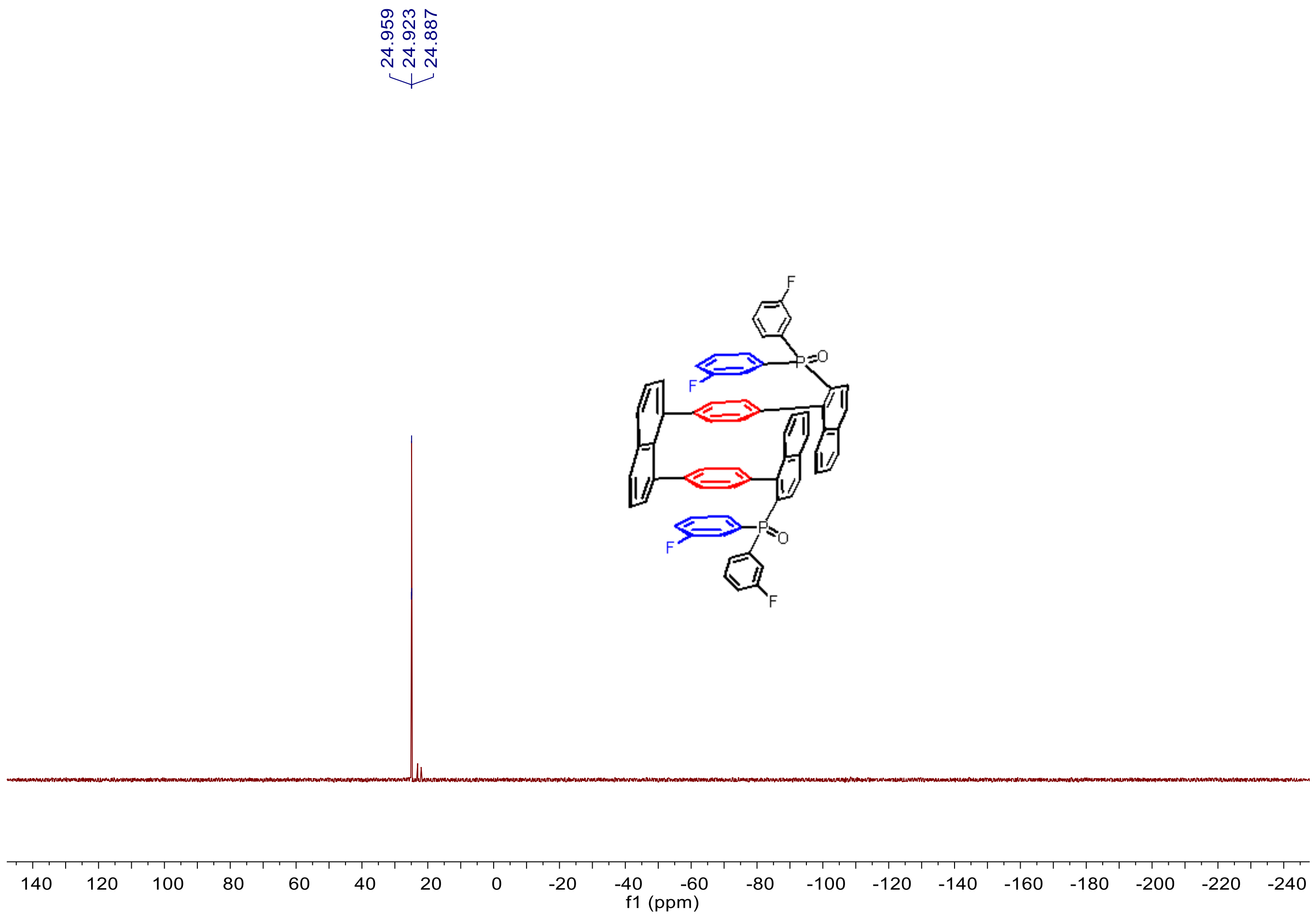

^{31}P NMR Spectrum of Compound 8l

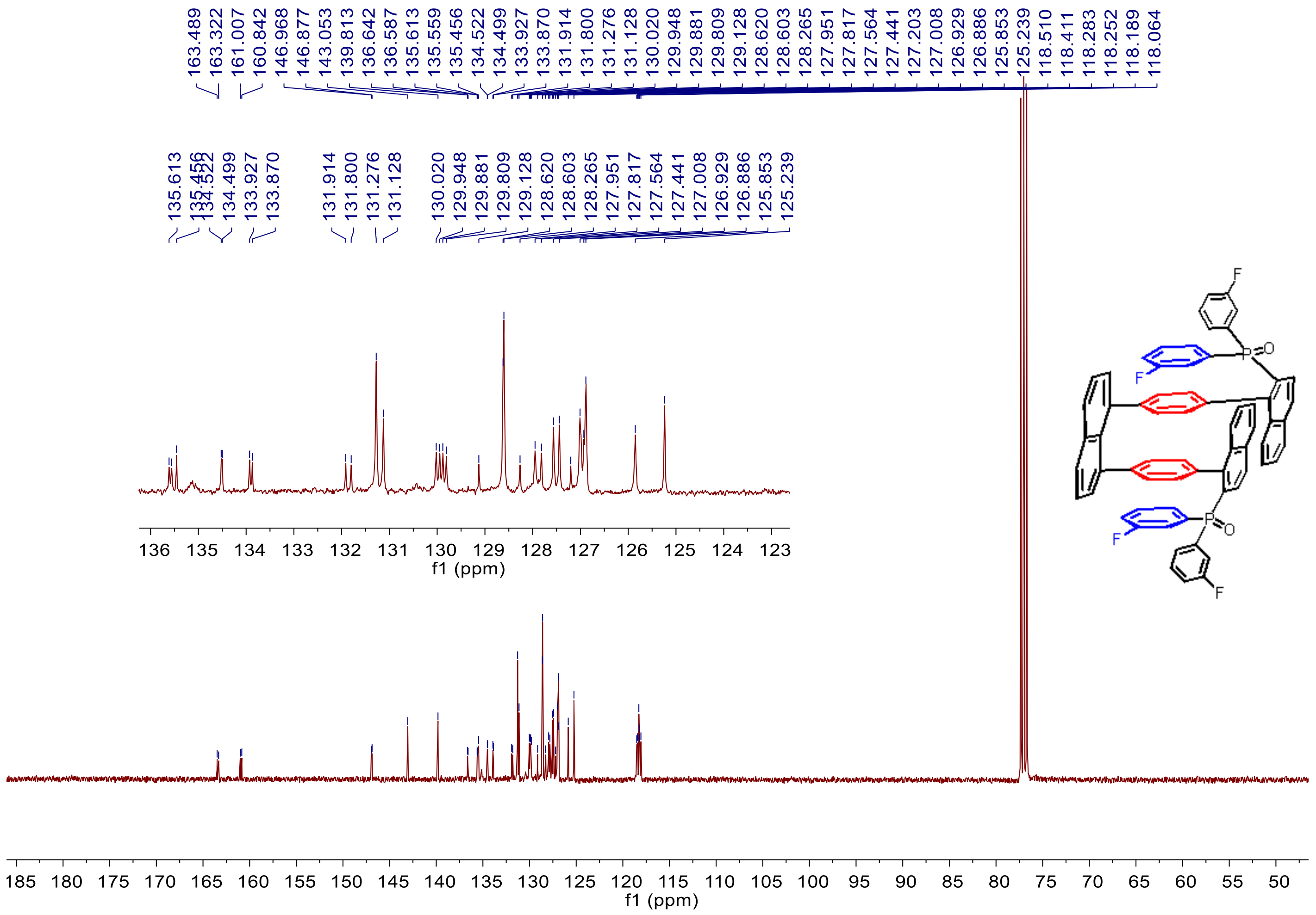

^{13}C NMR Spectrum of Compound 8l

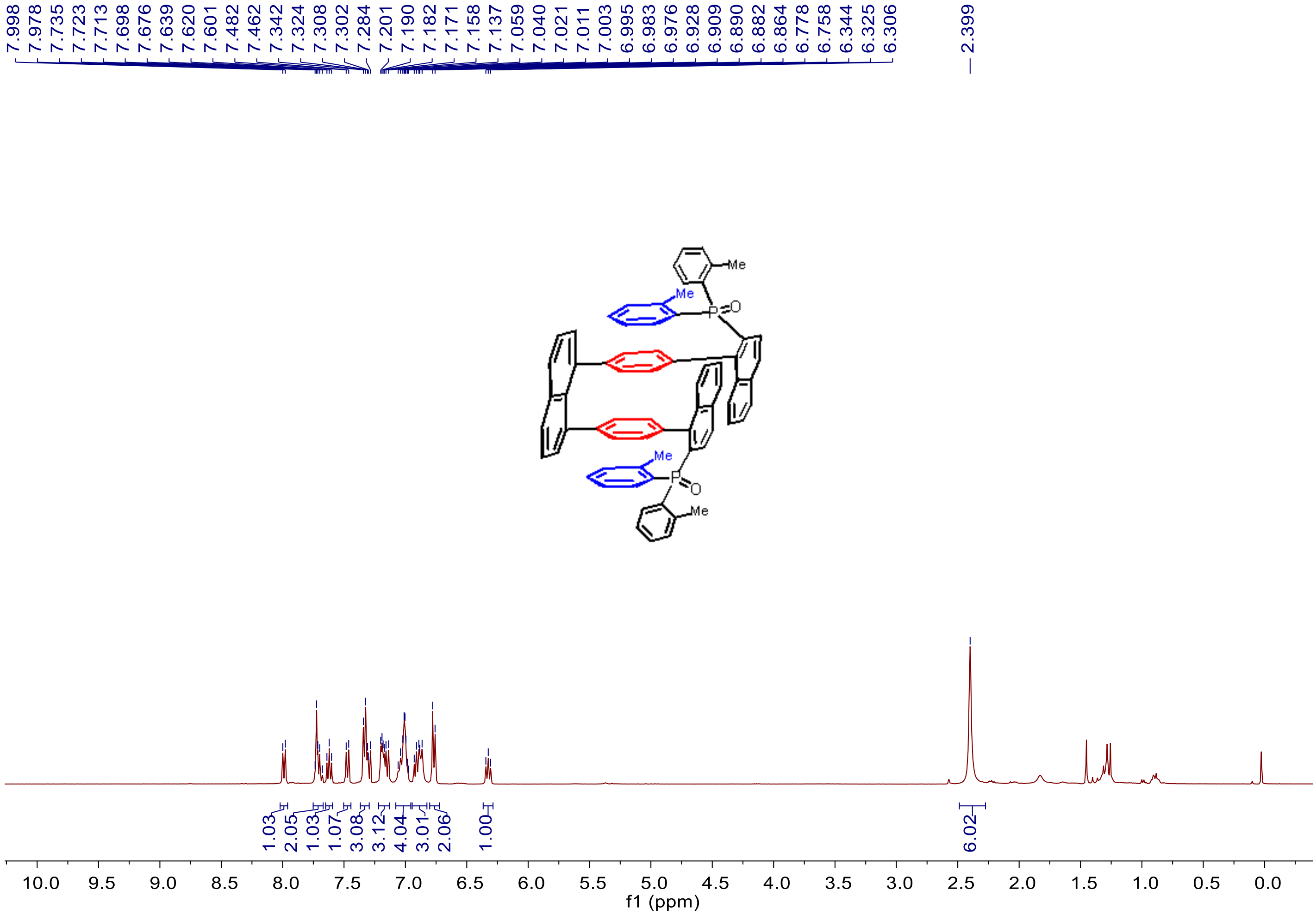

^{1}H NMR Spectrum of Compound 8m

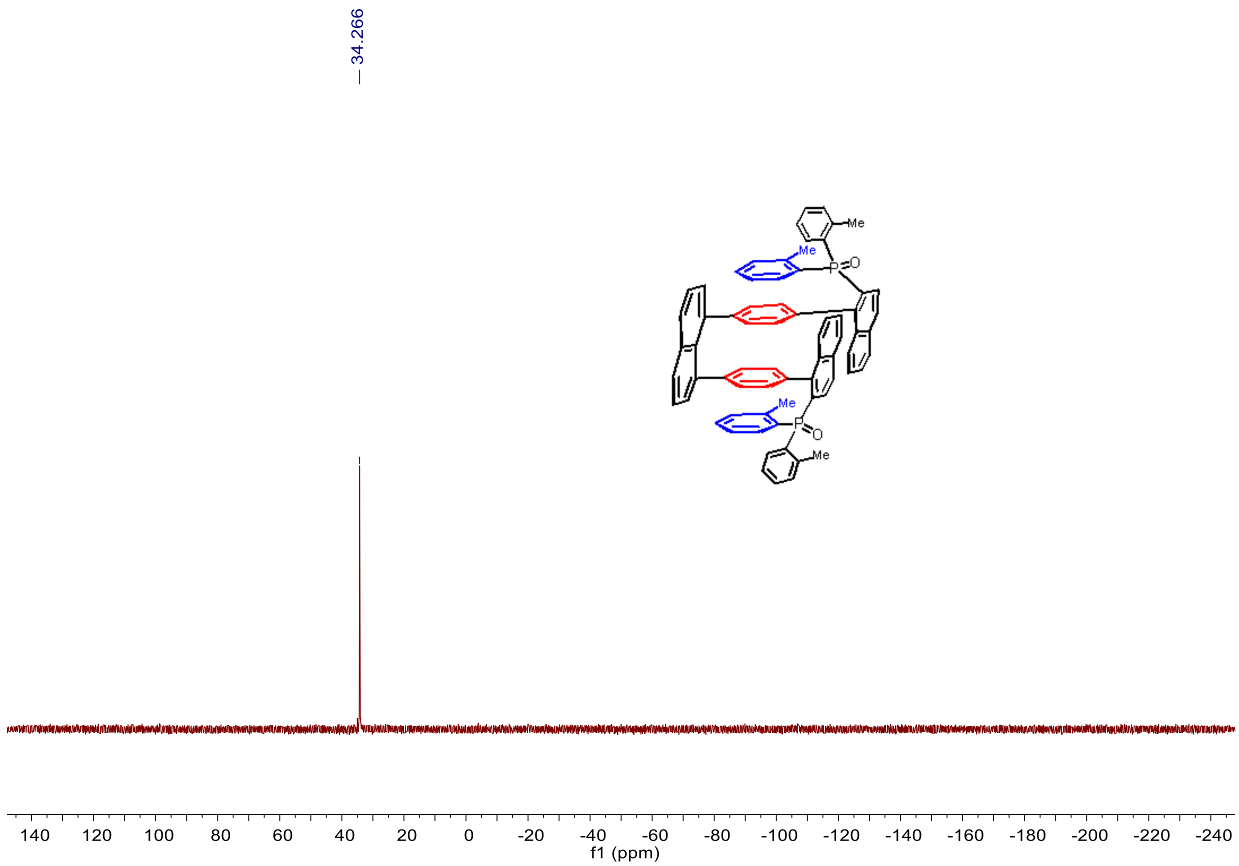

^{31}P NMR Spectrum of Compound 8m

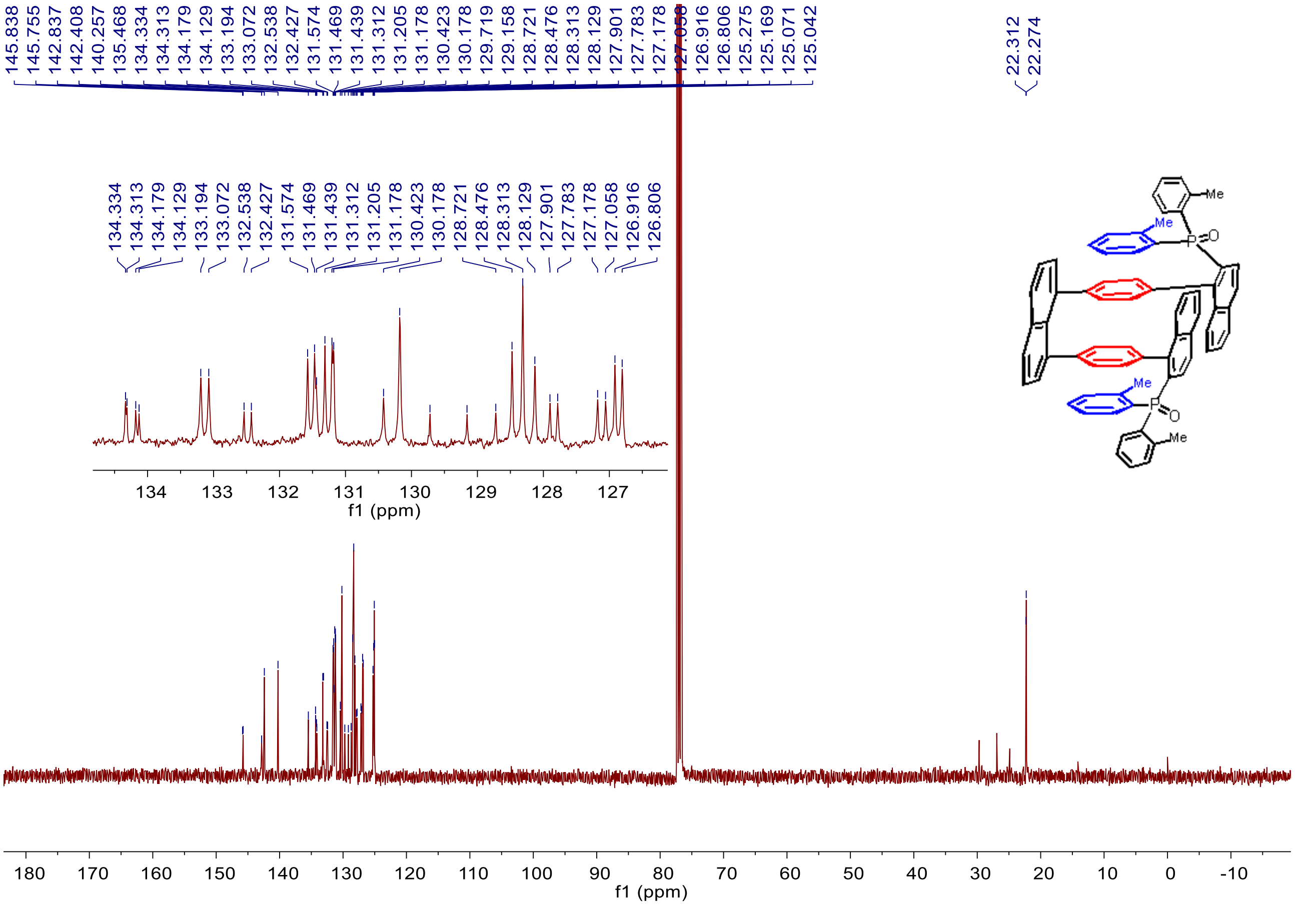

^{13}C NMR Spectrum of Compound 8m

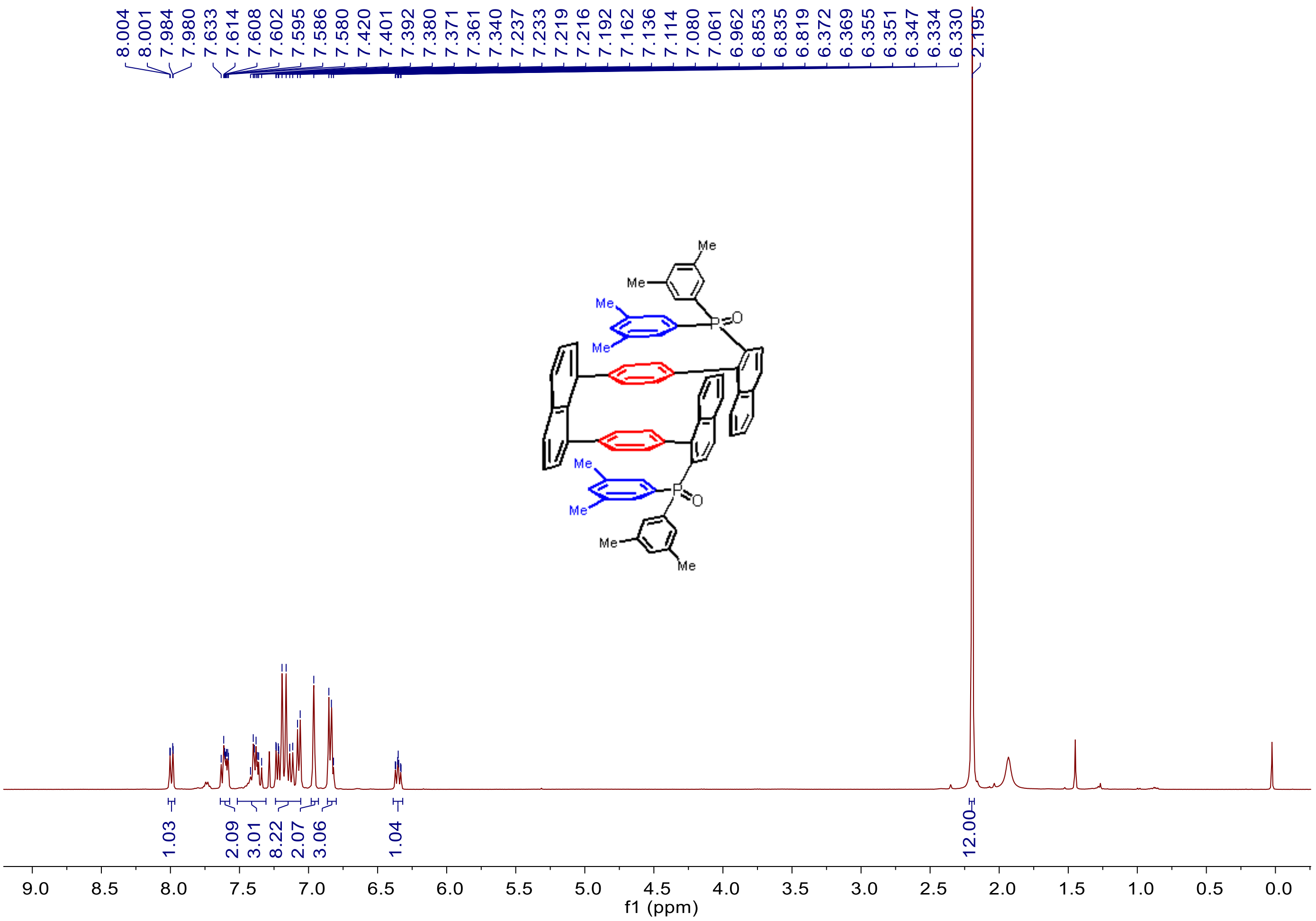

^{1}H NMR Spectrum of Compound 8n

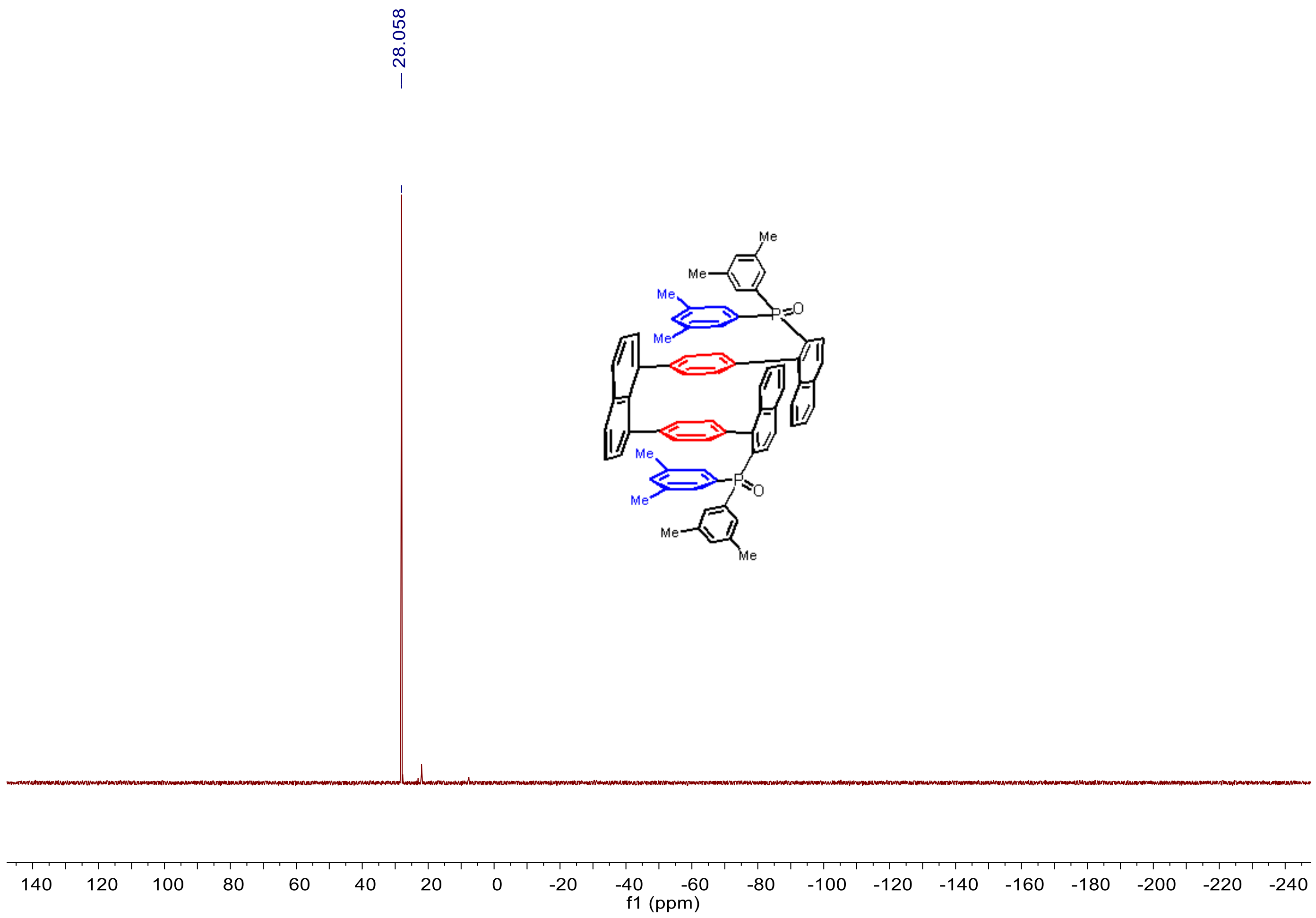

^{31}P NMR Spectrum of Compound 8n

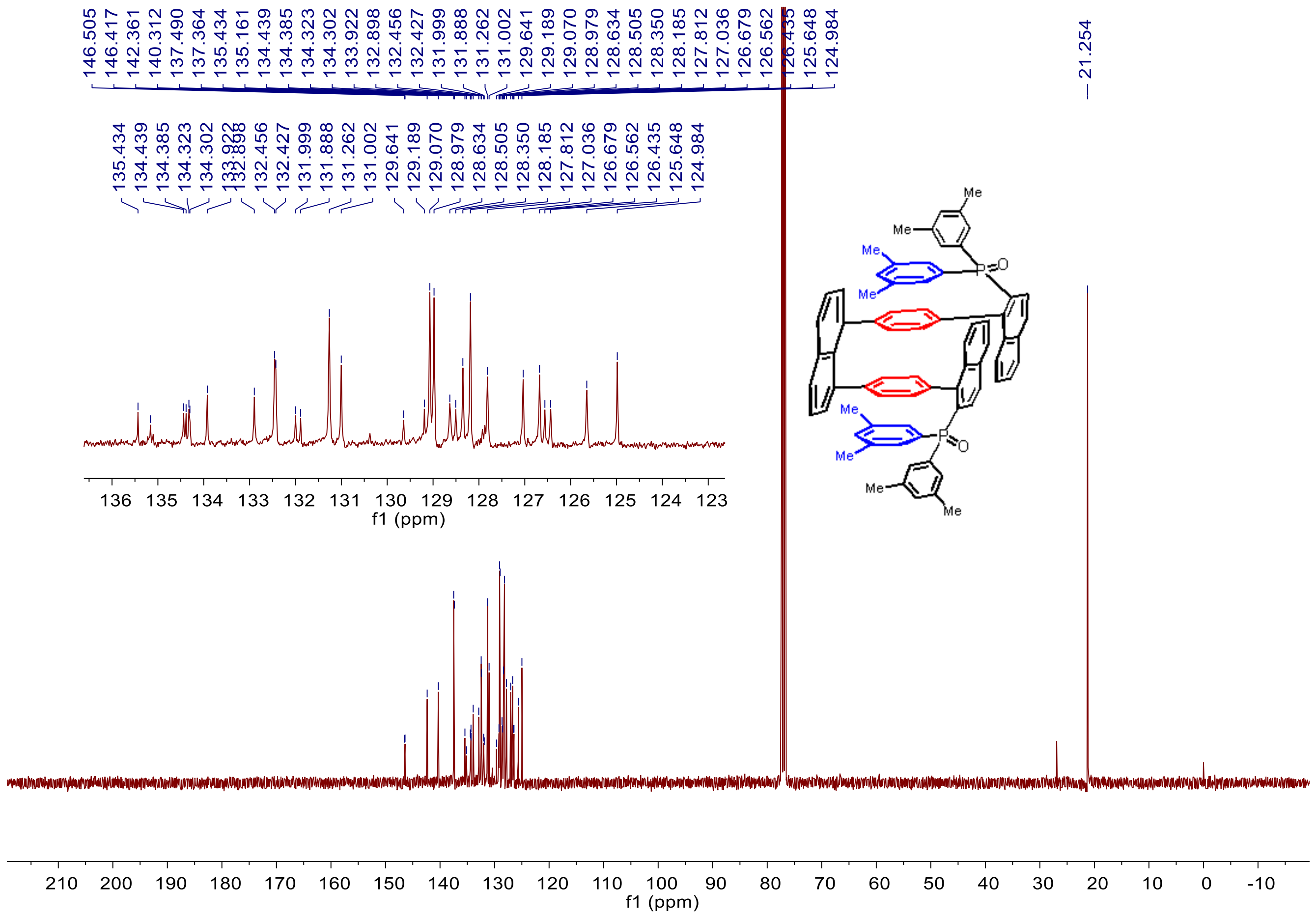

^{13}C NMR Spectrum of Compound 8n

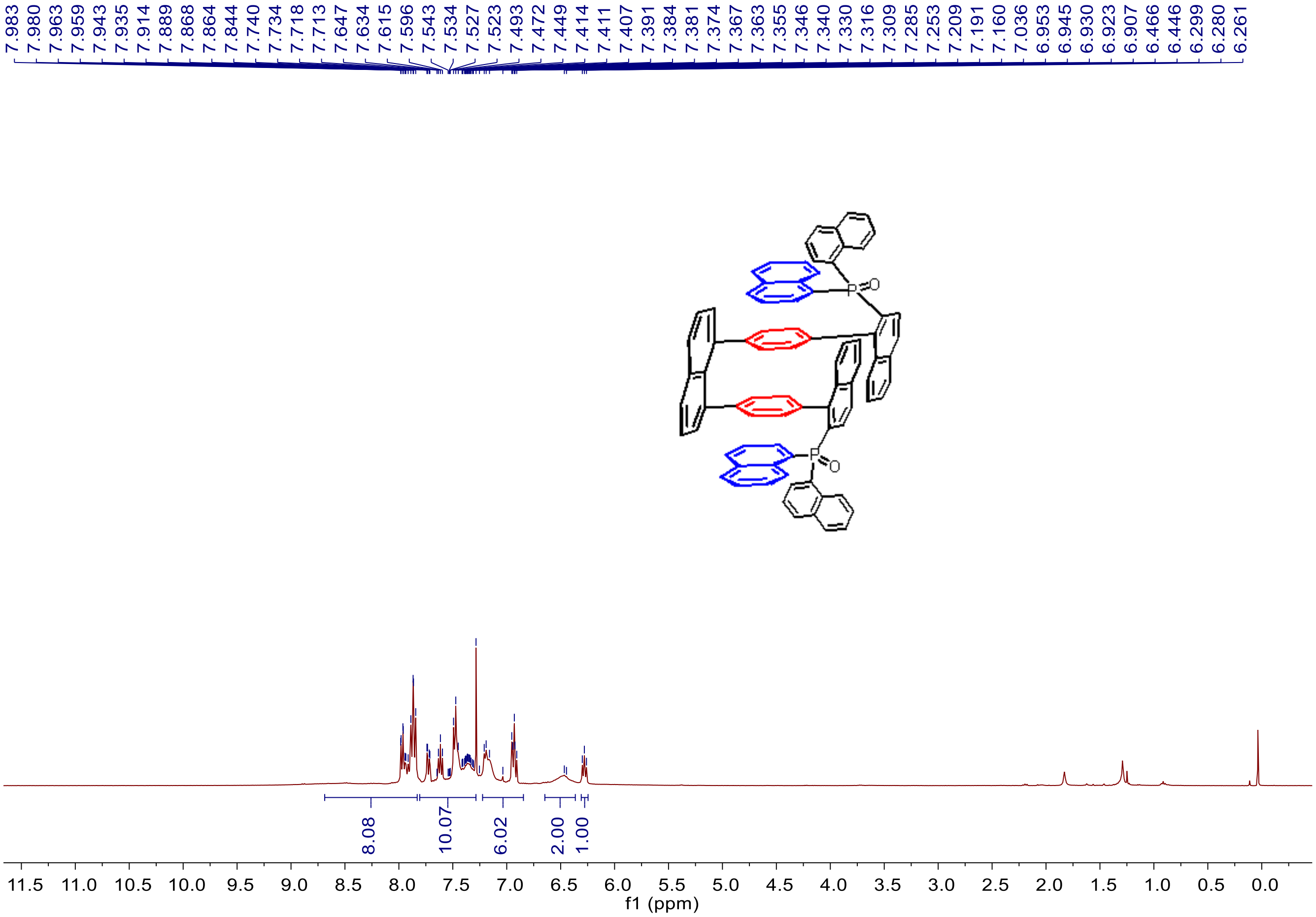

^{1}H NMR Spectrum of Compound 8o

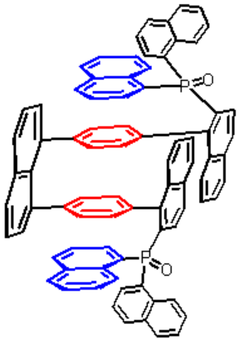

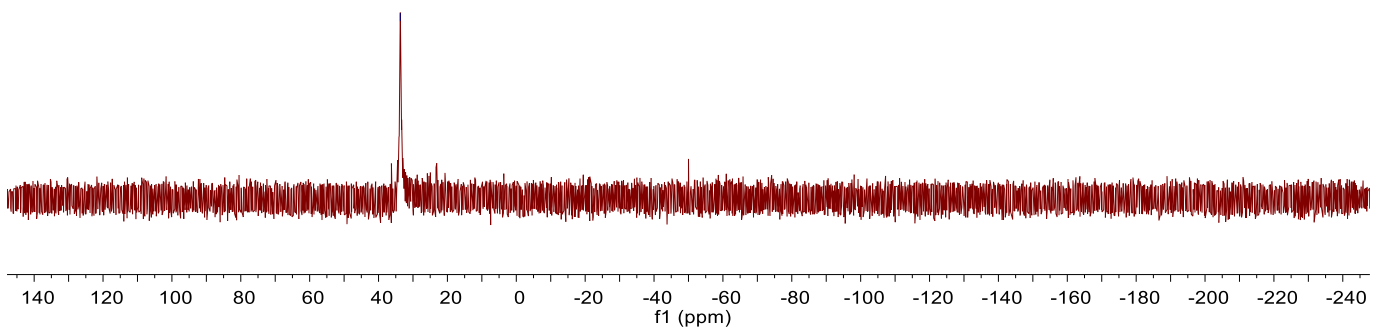

^{31}P NMR Spectrum of Compound 8o

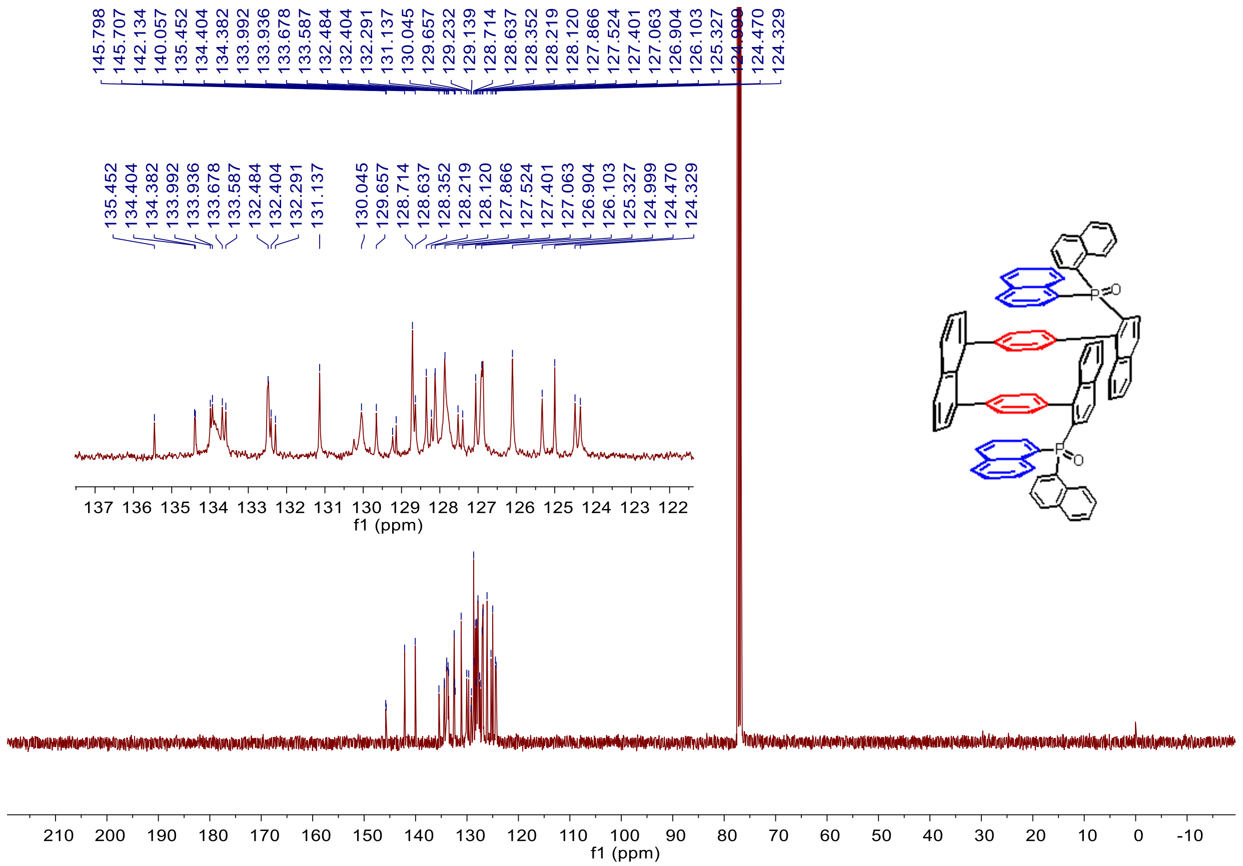

^{13}C NMR Spectrum of Compound 8o

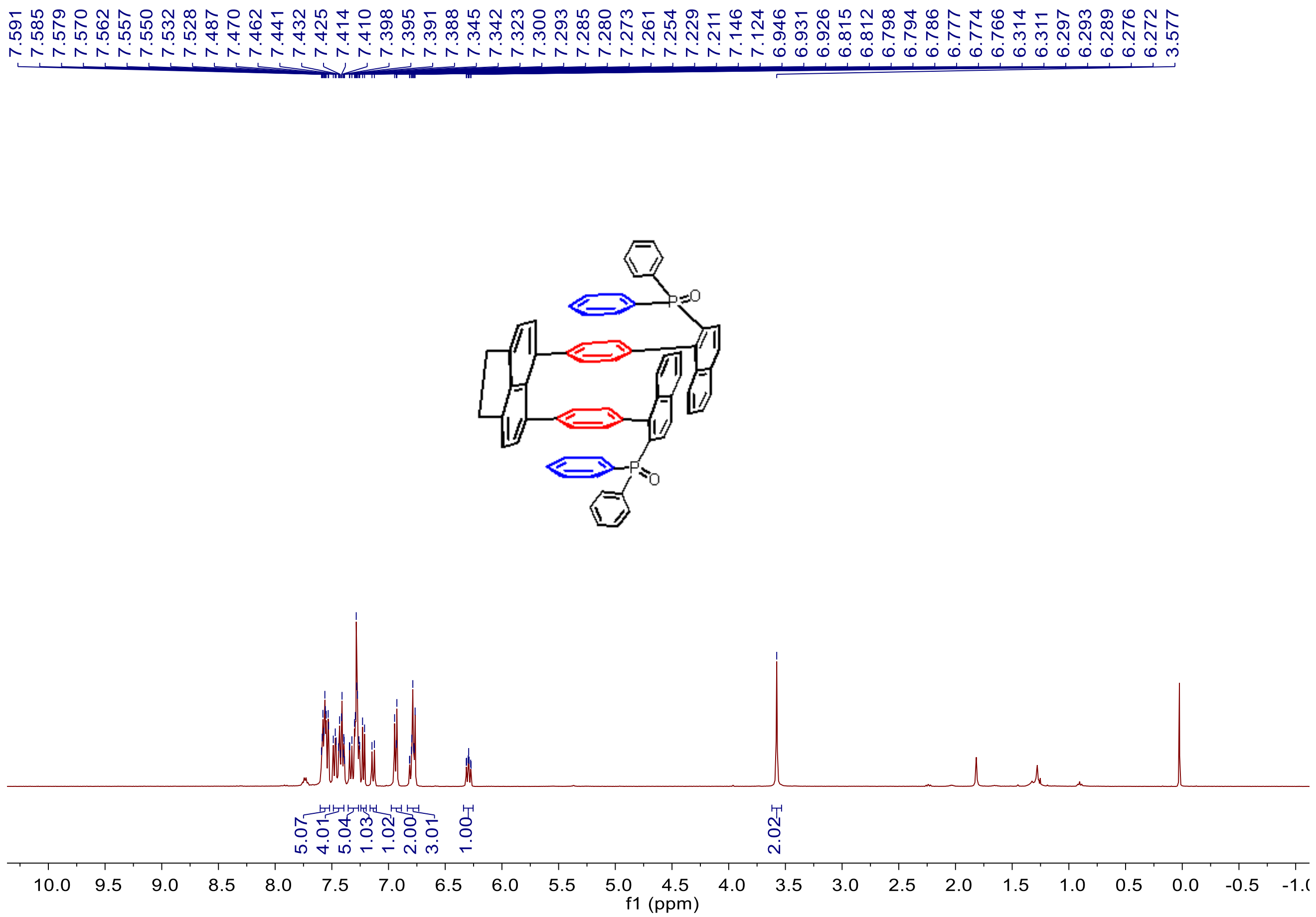

^{1}H NMR Spectrum of Compound 8p

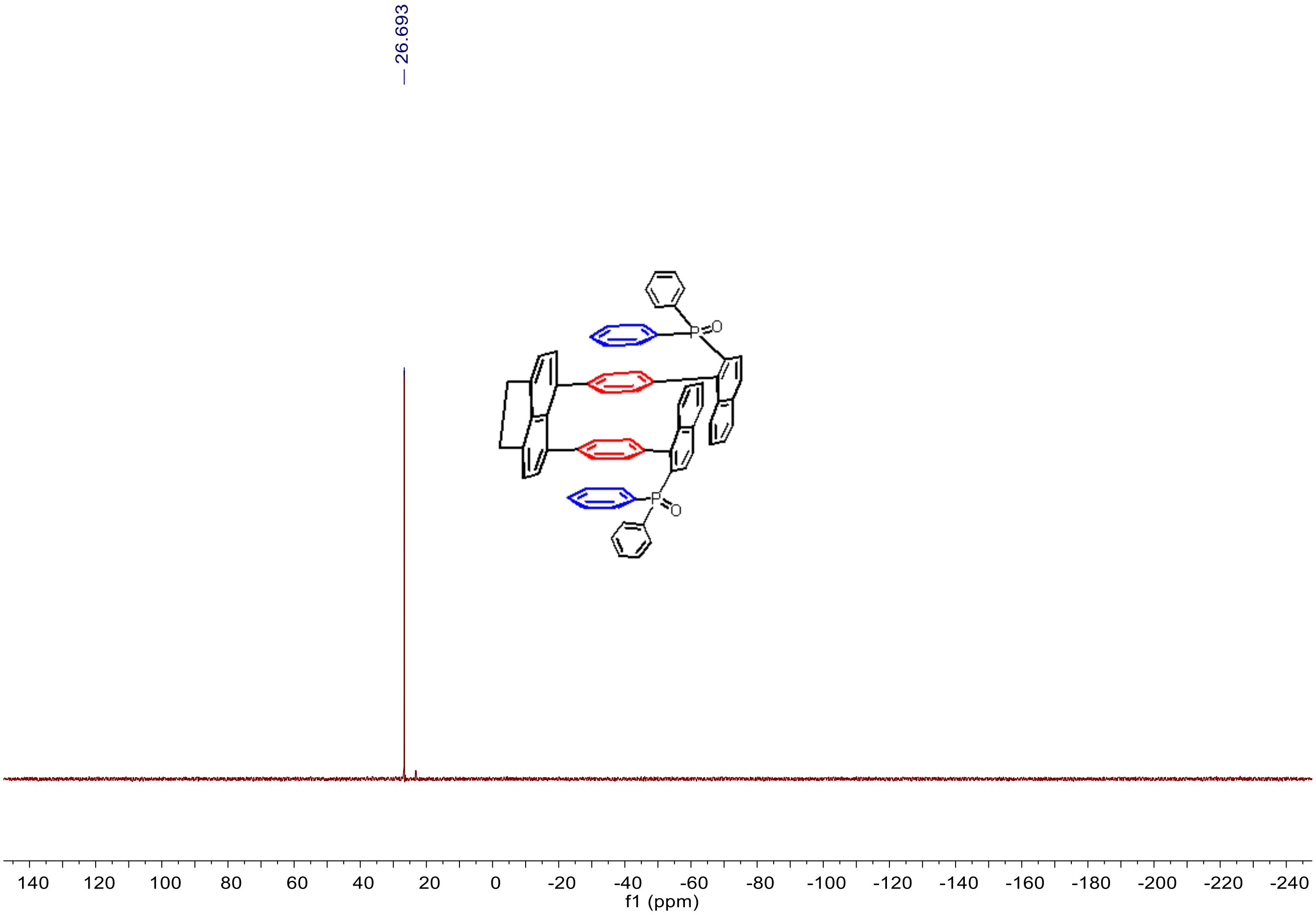

^{31}P NMR Spectrum of Compound 8p

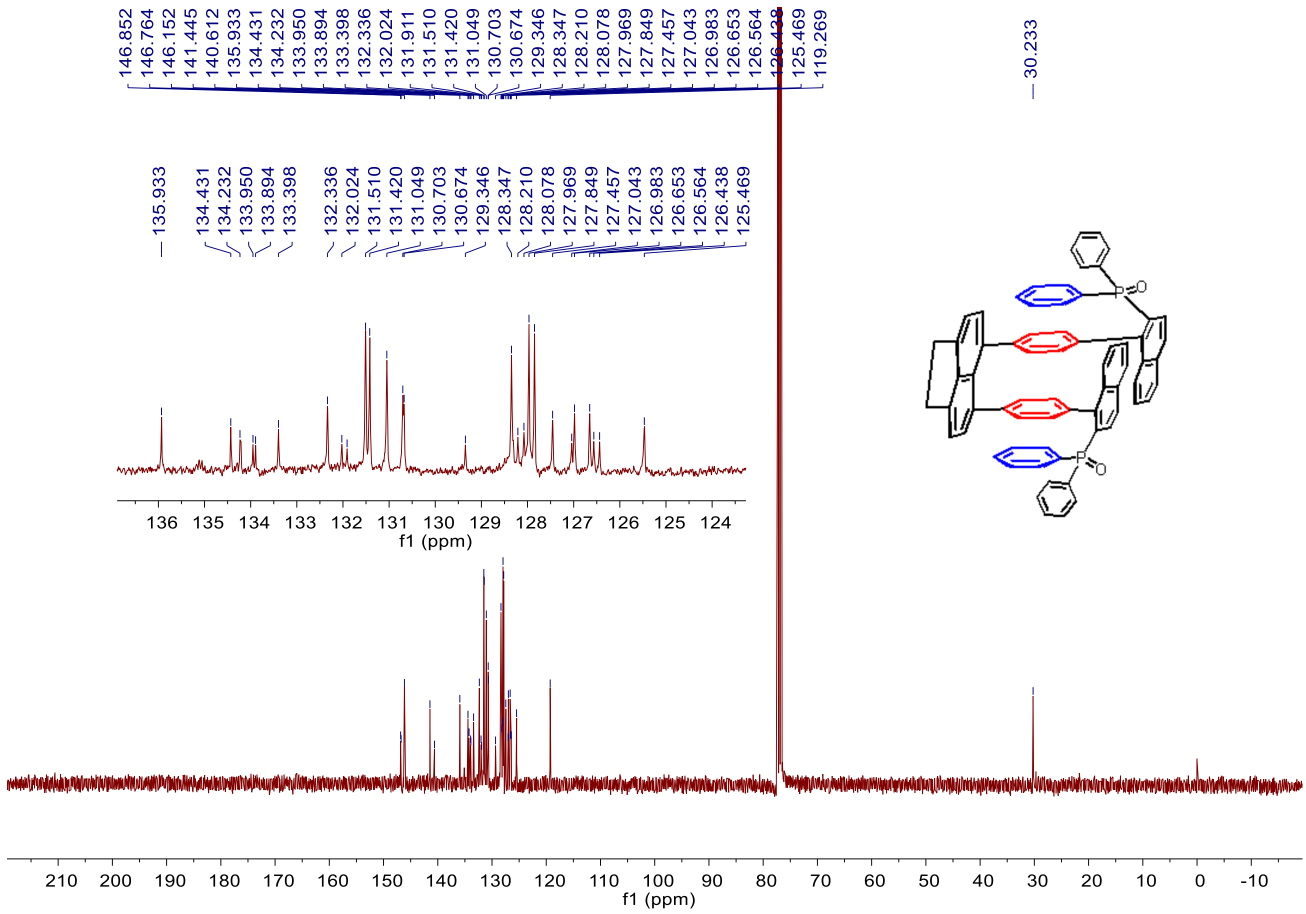

^{13}C NMR Spectrum of Compound 8p
